# Oral Chinese herbal formulas for premature ovarian insufficiency: a systematic review, meta-analysis, and exploratory therapeutic-principle subgroup analysis

**DOI:** 10.3389/fendo.2026.1870487

**Published:** 2026-08-05

**Authors:** Yihong Wu, Sha Li, Guichun Liu, Xurui Zheng, Jin Cheng, Xiao Jiang

**Affiliations:** 1 First Teaching Hospital of Tianjin University of Traditional Chinese Medicine, National Clinical Research Center for Chinese Medicine Acupuncture and Moxibustion, Tianjin, China; 2 Faculty of Health and Physical Education, Xi’an Fanyi University, Xi’an, China; 3 College of Basic Medicine, Shaanxi University of Chinese Medicine, Xi’an, China; 4 The Affiliated Hospital of Xuzhou Medical University, Xuzhou, China

**Keywords:** Chinese herbal formulas, Chinese herbal medicine, meta-analysis, ovarian reserve, premature ovarian failure, premature ovarian insufficiency, systematic review

## Abstract

**Introduction:**

Premature ovarian insufficiency (POI) is characterized by impaired ovarian endocrine function and reduced ovarian reserve.

**Methods:**

We searched eight bibliographic databases, ClinicalTrials.gov, the World Health Organization International Clinical Trials Registry Platform (WHO ICTRP), and the Chinese Clinical Trial Registry (ChiCTR) from inception to May 30, 2026, for parallel-group randomized controlled trials comparing HRT-based regimens with pure oral Chinese herbal formulas within three prespecified therapeutic-principle categories. This systematic review was registered in the International Prospective Register of Systematic Reviews (PROSPERO; CRD420251234457).

**Results:**

Menstrual return or resumption was designated as the patient-important outcome, while follicle-stimulating hormone (FSH) and anti-Müllerian hormone (AMH) were designated as primary surrogate biomarkers. Forty-one HRT-controlled trials met the prespecified therapeutic-principle criteria. No clear between-group difference was detected for menstrual return or resumption (4 studies, 266 participants; odds ratio [OR] 1.523, 95% confidence interval [CI] 0.315 to 7.371; I^2^ = 86.16%). Point estimates favored oral herbal formulas for FSH (37 studies; mean difference [MD] −5.563, 95% CI −8.230 to −2.896; I^2^ = 99.13%) and AMH (12 studies; MD 0.167, 95% CI 0.035 to 0.299; I^2^ = 98.17%), but both were surrogate outcomes and their prediction intervals crossed the null. Directionally favorable estimates were also observed for luteinizing hormone (LH), estradiol (E2), antral follicle count (AFC), and peak systolic velocity (PSV), although these were surrogate or exploratory outcomes with substantial uncertainty. Fewer adverse events were reported with oral herbal formulas, but safety remained unestablished because ascertainment and reporting were incomplete. Therapeutic-principle analyses yielded an exploratory subgroup signal only for FSH and did not establish comparative superiority among kidney-tonifying alone (PK), kidney-tonifying and liver-soothing (KL), and kidney-tonifying and blood-activating (KB) categories. All assessed outcomes were rated as very low-certainty evidence.

**Discussion:**

Current evidence does not support the routine use of any specific Chinese herbal formula or therapeutic-principle category. Guideline-based management, including HRT when clinically appropriate, should remain standard care, and rigorously designed multicenter trials using patient-important outcomes and standardized safety reporting are needed.

**Systematic review registration:**

https://www.crd.york.ac.uk/prospero/, identifier CRD420251234457.

## Introduction

Premature ovarian insufficiency (POI) is characterized by impaired ovarian function before the age of 40 years, with menstrual disturbance, elevated gonadotropin levels, and diminished ovarian reserve (1). The condition was initially known as premature ovarian failure (POF); however, the current preferred term is POI because it more accurately reflects the variable and sometimes intermittent nature of ovarian function in affected women (2). POI has traditionally been estimated to affect about 1% of women under 40 years, although more recent guideline updates suggest the prevalence may be higher (3), and is associated with infertility, vasomotor symptoms, osteoporosis, cardiovascular disease, and adverse psychosocial outcomes (4).

POI has a multifactorial pathophysiology, with follicular depletion and impaired ovarian endocrine function representing two major pathological features (5). These changes disrupt feedback within the hypothalamic–pituitary–ovarian (HPO) axis, leading to hormonal imbalance and diminished ovarian reserve, as reflected by altered gonadotropin and estradiol levels and changes in ovarian reserve-related indicators. Among the proposed mechanisms, oxidative stress and dysregulation of the HPO axis have been implicated as important contributors to ovarian injury and hormonal disturbance (6).

Hormone replacement therapy (HRT) is the standard treatment for women with POI and helps relieve hypoestrogenic symptoms while reducing long-term risks such as osteoporosis and cardiovascular disease (3). However, HRT is not primarily intended to restore ovarian function or fertility, which has led to interest in non-hormonal therapeutic approaches. In East Asia, oral Chinese herbal formulas are used in clinical practice for POI as traditional non-hormonal therapeutic approaches, including formulas described by therapeutic principles such as kidney tonification (Bu Shen), liver soothing (Shu Gan), and blood activation (Huo Xue). In this review, these labels are used as trial-level therapeutic descriptors for subgrouping rather than as validated biomedical categories. Although such formulas are widely used in practice, their role is still not well defined in international evidence-based recommendations (7).

Published trials and previous evidence syntheses have reported changes in selected endocrine and ovarian reserve-related markers among women with POI, but these surrogate findings do not establish patient-important clinical benefit (8–10). Even so, the pooled evidence remains limited by trial quality, inconsistent outcome definitions, and substantial heterogeneity. A more structured look at intervention heterogeneity may therefore help make the current evidence base easier to interpret.

Earlier meta-analyses generally pooled oral Chinese herbal formulas as a single intervention, which may have obscured clinically relevant differences in therapeutic rationale and formula composition (8–10). In the present review, therapeutic-principle classification was applied as a structured, rule-based exploratory framework to organize intervention heterogeneity and generate hypotheses. We therefore synthesized randomized evidence for pure oral Chinese herbal formulas versus HRT-based comparators, evaluated a strict menstrual return or resumption outcome alongside surrogate biomarkers, and conducted exploratory therapeutic-principle analyses. A supplementary network pharmacology analysis was performed only as hypothesis-generating context.

## Methods

### Protocol registration and reporting standards

We registered this systematic review and meta-analysis in the International Prospective Register of Systematic Reviews (PROSPERO; CRD420251234457) and reported the review according to the Preferred Reporting Items for Systematic Reviews and Meta-Analyses (PRISMA) 2020 statement (11). The protocol can be accessed through the corresponding PROSPERO registration record. After registration, methodological details were refined to clarify the HRT-controlled eligibility restriction, the outcome hierarchy, therapeutic-principle classification certainty, REML modeling, prediction intervals, sensitivity analyses, and GRADE assessment; these amendments did not change the core review question. The intervention scope was prespecified in the registered protocol and was restricted to pure oral Chinese herbal formulas that could be classified into one of three therapeutic-principle categories: kidney-tonifying alone, kidney-tonifying plus liver-soothing, or kidney-tonifying plus blood-activating. This eligibility restriction defined the scope of the review and was distinct from the subsequent exploratory comparisons among the three categories. The PK/KL/KB therapeutic-principle subgroup framework was prespecified in the protocol, whereas the Level 1–3 classification-certainty scheme was added after registration to improve transparency and support sensitivity analysis.

The review question was framed according to the PICOS framework. The population was women diagnosed with premature ovarian insufficiency (POI), and the intervention was a pure orally administered Chinese herbal formula falling within one of the three prespecified therapeutic-principle categories: kidney-tonifying alone, kidney-tonifying plus liver-soothing, or kidney-tonifying plus blood-activating. Formulas outside these three prespecified categories, or formulas for which the therapeutic principle could not be reliably determined from the reported therapeutic description, TCM pattern, or composition, were outside the predefined intervention scope. Eligible comparators for the primary synthesis were HRT-based regimens without clomiphene-containing active endocrine comparators. The outcomes comprised strict menstrual return or resumption, endocrine and ovarian-reserve surrogate markers, an exploratory hemodynamic outcome, adverse events, and a supplementary variably defined composite outcome; parallel-group RCTs were eligible.

Therapeutic-principle subgroup analyses and network pharmacology analyses were defined as exploratory and hypothesis-generating components rather than confirmatory assessments of comparative efficacy or causal mechanisms.

### Search strategy

We conducted a comprehensive literature search across eight electronic databases, including PubMed, Embase, Web of Science, the Cochrane Library, China National Knowledge Infrastructure (CNKI), Wanfang Data, VIP Database, and China Biology Medicine (CBM), from the inception of the database to May 30, 2026, without language restrictions. The search strategy combined the use of controlled vocabulary terms (e.g., MeSH or Emtree terms, where applicable) and free-text terms related to premature ovarian insufficiency (POI), premature ovarian failure (POF), primary ovarian insufficiency, traditional Chinese medicine, Chinese herbal medicine, and randomized controlled trials.

To identify ongoing, unpublished, or additionally registered studies, we also searched ClinicalTrials.gov, the World Health Organization International Clinical Trials Registry Platform (WHO ICTRP), and the Chinese Clinical Trial Registry (ChiCTR). The reference lists of relevant reviews and included studies were also screened manually to identify additional eligible studies. All retrieved records were imported into EndNote 2025 for managing records and removing duplicates. The complete search strategies for all databases and trial registries are provided in Additional file 1: **Supplementary Table S2**.

### Eligibility criteria

#### Types of studies

In this review, only parallel-group randomized controlled trials (RCTs) were included. Non-randomized studies, observational studies, reviews, case reports, animal experiments, conference abstracts without sufficient extractable data, and duplicate or potentially overlapping publications were excluded from the primary quantitative synthesis. When potentially overlapping publications were identified, only the most complete and extractable report was retained in the primary quantitative synthesis to avoid double-counting participants.

#### Participants

Reports using the historical term POF were included only when participants were younger than 40 years and the reported diagnostic features were consistent with ovarian insufficiency. POI is used as the preferred contemporary term throughout the manuscript, whereas POF is retained only when reproducing the terminology used in individual historical trial reports or reference titles (12).

#### Interventions and comparators

The intervention of interest was a pure orally administered Chinese herbal formula, including decoctions, granules, capsules, pills, or Chinese patent medicines with clearly reported herbal composition. Eligibility additionally required sufficient information to classify the formula within one of three prespecified therapeutic-principle categories: kidney-tonifying alone (PK), kidney-tonifying and liver-soothing (KL), or kidney-tonifying and blood-activating (KB). Formulas based on other therapeutic principles, or those lacking sufficient information for reliable classification within this framework, were excluded at the eligibility stage. This eligibility restriction was distinct from the subsequent exploratory subgroup analyses. Eligible comparisons were pure oral Chinese herbal formulas versus HRT-based conventional treatment.

Studies involving acupuncture, moxibustion, external therapy, or other non-oral traditional Chinese medicine interventions were excluded, as were trials in which the experimental arm combined Chinese herbal formulas with HRT or other active non-herbal treatments. Trials with placebo comparators, clomiphene-containing active endocrine comparators, add-on herbal-plus-HRT interventions, complex or unbalanced co-interventions, unclear herbal composition, or potential participant overlap were excluded from the primary synthesis. Among eligible studies, the three prespecified categories were also used for exploratory therapeutic-principle subgroup analyses. Definitions of the synthesis scope, comparator restrictions, excluded records, and therapeutic-principle classification are provided in Additional file 1: **Supplementary Tables S3–S6**.

#### Treatment duration

Studies with a treatment duration of less than three menstrual cycles or three months were excluded.

Treatment duration was extracted and categorized as ≤3 treatment cycles/months or >3 treatment cycles/months for exploratory subgroup and univariable meta-regression analyses.

#### Outcome measures

Menstrual return or resumption was designated as the patient-important menstrual outcome. FSH and AMH were designated primary surrogate biomarkers; LH and E2 were secondary endocrine surrogates; AFC was a secondary ovarian reserve-related surrogate; PSV was an exploratory ovarian hemodynamic outcome; adverse events were assessed as a safety outcome; and overall clinical effectiveness was retained only as a supplementary, variably defined composite outcome. PSV referred to ovarian arterial peak systolic velocity as reported in the original studies; laterality and Doppler measurement methods were not consistently specified.

Menstrual return or resumption was restricted to directly reported return or resumption of menstruation under the prespecified strict definition; broader study-defined menstrual-improvement composites were not pooled as the main menstrual outcome. Overall clinical effectiveness was retained only as a supplementary, variably defined composite outcome and was not treated as menstrual return, ovarian recovery, or robust clinical-efficacy evidence. Because trial-specific definitions commonly combined symptoms, menstrual changes, and laboratory indicators, this outcome was interpreted cautiously and downgraded for indirectness. Studies entered an outcome-specific quantitative synthesis only when extractable data were available for the corresponding strict menstrual, surrogate, exploratory, composite, or safety outcome. Studies that did not report a prespecified outcome of interest or provided data in a non-extractable format were excluded from the corresponding quantitative synthesis. Outcome availability is summarized in **Table 1**.

**Table 1:** Characteristics of the 41 included randomized controlled trials.

Study	Sample size (T/C)	Intervention	Comparator	Duration	Outcome measures
Hua et al. (2012)	60 (30/30)	Bu-Shui-Rou-Mu Formula	estrogen tablets + medroxyprogesterone acetate tablets	12 weeks	(1)\(3)\(4)
Xu et al. (2012)	64 (33/31)	Modified Yi-Jing Decoction	estradiol valerate tablets + medroxyprogesterone acetate	16 weeks	(1)\(2)\(3)\(4)\(9)
Jin (2013)	174 (86/88)	Kidney-tonifying and Blood-activating Formula	estrogen tablets + medroxyprogesterone acetate tablets	24 weeks	(1)\(2)\(3)\(4)\(6)\(8)\(9)
Xu (2013)	48 (24/24)	Kidney-tonifying Menstruation-regulating Formula	estrogen tablets + medroxyprogesterone acetate tablets	12 weeks	(1)\(2)\(3)\(4)\(8)
Xu et al. (2014)	64 (32/32)	Kidney-tonifying Menstruation-regulating Formula	estrogen tablets + medroxyprogesterone acetate tablets	24 weeks	(1)\(2)\(3)\(4)\(8)
Wen (2015)	50 (25/25)	Kidney-tonifying Menstruation-restoring Formula	medroxyprogesterone acetate + estradiol valerate tablets	24 weeks	(1)\(2)\(3)\(4)\(8)\(9)
Cheng (2016)	52 (26/26)	Modified Yu-Yin-Ling Formula	estrogen tablets + medroxyprogesterone acetate	12 weeks	(1)\(2)\(3)\(4)\(8)\(9)
Zeng (2017)	80 (40/40)	Modified Er-Xian Decoction	estrogen tablets + medroxyprogesterone acetate tablets	24 weeks	(1)\(3)\(4)
Li LL (2018)	78 (39/39)	Kidney-tonifying and Liver-soothing Formula	estradiol valerate tablets + progesterone capsules	12 weeks	(1)\(2)\(3)\(4)\(6)\(8)\(9)
Yu et al. (2018)	83 (42/41)	Zuo-Gui Pill	estradiol valerate tablets + progesterone capsules	24 weeks	(1)\(3)\(4)
Chen et al. (2019)	120 (60/60)	Kidney-tonifying Menstruation-regulating Formula	estradiol valerate tablets + progesterone capsules	24 weeks	(1)\(2)\(3)\(4)\(5)\(8)\(9)
Li X (2018)	80 (40/40)	Modified Er-Xian Decoction	estradiol valerate tablets + progesterone capsules	12 weeks	(1)\(2)\(3)\(4)\(8)
Lu (2019)	80 (40/40)	Kidney-tonifying Menstruation-regulating Formula	estrogen tablets + medroxyprogesterone acetate tablets	12 weeks	(1)\(2)\(3)\(4)\(9)
Yuan (2019)	90 (45/45)	Er-Xian Decoction	estradiol tablets + dydrogesterone tablets	12 weeks	(2)
Zeng et al. (2019)	63 (32/31)	Kidney-tonifying and Blood-activating Formula	estradiol–cyproterone acetate tablets	12 weeks	(1)\(3)\(4)\(5)
Dong (2020)	82 (41/41)	Kidney-tonifying and Blood-activating Formula	estradiol valerate tablets + medroxyprogesterone acetate tablets	24 weeks	(1)\(3)\(4)\(5)\(7)
Li (2020)	54 (28/26)	Modified Gui-Xian Decoction	estradiol tablets + dydrogesterone tablets	12 weeks	(1)\(2)\(3)\(4)\(8)\(9)
Luo (2020)	60 (30/30)	Yi-Jing Decoction	estradiol tablets + dydrogesterone tablets	12 weeks	(1)\(2)\(3)\(4)\(5)\(6)\(8)
Sui (2021)	122 (60/62)	Menstruation-regulating Formula	estradiol tablets + dydrogesterone tablets	24 weeks	(2)\(5)\(7)\(9)
Ling (2021)	80 (40/40)	Kidney-tonifying and Blood-activating Formula	estradiol valerate tablets + dydrogesterone tablets	24 weeks	(1)\(2)\(3)\(8)\(9)
Tang (2021)	80 (40/40)	Kidney-tonifying and Blood-activating Formula	estradiol tablets + dydrogesterone tablets	12 weeks	(1)\(2)\(3)\(4)\(5)\(6)\(8)\(9)
Wang XW (2021)	50 (25/25)	Kidney-tonifying and Liver-soothing Formula	estradiol tablets + dydrogesterone tablets	12 weeks	(1)\(2)\(3)\(4)
Wang (2022)	86 (43/43)	Modified Er-Xian Decoction	estradiol valerate tablets + dydrogesterone tablets	12 weeks	(1)\(2)\(3)\(4)\(8)
Huang et al. (2023)	92 (46/46)	Kidney-nourishing Fertility-supporting Formula (combination)	estradiol valerate tablets + progesterone capsules	12 weeks	(1)\(2)\(3)\(4)\(5)\(6)\(8)\(9)
Xia (2023)	120 (60/60)	Menstruation-regulating Formula	estradiol tablets + dydrogesterone tablets	24 weeks	(1)\(2)\(3)\(4)\(5)\(7)
Dong (2024)	60 (30/30)	Kidney-tonifying and Blood-activating Formula	estradiol tablets + dydrogesterone tablets	12 weeks	(1)\(2)\(3)\(4)\(6)\(8)\(9)
Ye (2024)	200 (100/100)	Zuo-Gui Pill combined with adjunct formula	estradiol valerate tablets + dydrogesterone	24 weeks	(1)\(2)\(3)\(4)\(5)\(9)
Ren (2025)	90 (45/45)	Kidney-tonifying and Liver-soothing Formula	estradiol tablets + dydrogesterone tablets	12 weeks	(1)\(2)\(3)\(4)\(8)\(9)
Xie (2025)	58 (29/29)	Kidney-tonifying and Menstruation-regulating Decoction	estradiol tablets + dydrogesterone tablets	12 weeks	(1)\(2)\(3)\(4)\(6)\(9)
Chen (2017)	50 (25/25)	Kidney-tonifying Menstruation-regulating Ointment Prescription	estrogen tablets + medroxyprogesterone acetate tablets	12 weeks	(1)\(3)\(4)
Feng (2024)	86 (43/43)	Wen-Jing Decoction	estradiol valerate tablets + progesterone	12 weeks	(1)\(2)\(3)\(4)\(5)\(6)\(7)\(9)
Jiang (2016)	50 (25/25)	Kidney-tonifying Cycle-regulating Therapy	estradiol valerate tablets + medroxyprogesterone acetate	12 weeks	(1)\(2)\(3)\(4)
Li (2012)	60 (33/27)	Yi-Jing Decoction	estradiol valerate tablets + medroxyprogesterone acetate	12 weeks	(1)\(2)\(3)\(9)
Song (2021)	73 (35/38)	Zhu’s Kidney-tonifying and Blood-activating Formula	estradiol tablets + dydrogesterone tablets	12 weeks	(1)\(3)\(4)\(5)\(7)\(8)
Wang HL (2021)	100 (50/50)	Kun-Tai Capsule	estradiol valerate tablets + progesterone	12 weeks	(1)\(2)\(4)\(5)\(9)
Wang (2019)	100 (50/50)	Kidney-tonifying Ovulation-promoting Decoction	estradiol valerate tablets + medroxyprogesterone acetate	12 weeks	(1)\(2)\(4)\(8)
Wang (2024)	123 (61/62)	Kidney-tonifying and Blood-activating Formula	estradiol tablets + dydrogesterone tablets	24 weeks	(2)\(8)
Xu et al. (2017)	46 (23/23)	Kidney-tonifying Menstruation-regulating Ointment	estrogen + medroxyprogesterone acetate	12 weeks	(1)\(2)\(3)\(4)\(8)\(9)
Zheng (2009)	60 (30/30)	Kidney-tonifying, Liver-regulating and Blood-activating Formula	diethylstilbestrol + medroxyprogesterone acetate	12 weeks	(1)\(2)\(3)\(4)\(9)
Zhou et al. (2025)	70 (35/35)	Kidney-nourishing and Meridian-soothing Decoction	estrogen tablets + medroxyprogesterone acetate tablets	12 weeks	(2)\(8)
Xue (2026)	60 (30/30)	Kidney-tonifying and Blood-nourishing Formula	estradiol valerate tablets + progesterone	12 weeks	(1)\(2)\(3)\(4)\(8)\(9)

For multi-arm studies, only the eligible pure oral Chinese herbal formula arm and the corresponding HRT control arm were included in the quantitative synthesis; add-on arms combining herbal formulas with HRT were excluded from the pooled analysis.

T, treatment group; C, control group; POI, premature ovarian insufficiency; POF, premature ovarian failure; NA, not available. Values are presented as mean ± SD unless otherwise specified. Outcome codes: (1) follicle-stimulating hormone (FSH); (2) overall clinical effectiveness; (3) estradiol (E2); (4) luteinizing hormone (LH); (5) anti-Müllerian hormone (AMH); (6) antral follicle count (AFC); (7) peak systolic velocity (PSV); (8) adverse events; (9) menstrual return or resumption.

### Study selection and data extraction

Two reviewers independently screened titles and abstracts, and then assessed the full text according to the predefined eligibility criteria. Disagreements between them were resolved through discussion, and unresolved discrepancies were decided by a third reviewer.

Two reviewers independently extracted data using a standardized form. Extracted data included study and participant characteristics, intervention and HRT comparator details, arm-level means, SDs and sample sizes, dichotomous event counts and denominators, unit-conversion flags, SD-source information, baseline and endpoint availability, excluded arms, and risk-of-bias data.

Potentially overlapping reports and comparator mismatches were audited using author names, formulas, sample characteristics, baseline data, outcomes, and comparator regimens, with final decisions documented in **Additional File 1**: **Supplementary Table S4** and **Additional File 2**. For multi-arm trials, only the eligible pure oral herbal-formula arm and its corresponding HRT control arm were extracted to avoid double-counting participants. Missing study-level covariates, such as mean age, were not imputed for meta-regression analyses. The complete cleaned arm-level dataset, source audits, final analysis results, and Stata command and QC logs are provided in **Additional File 2**.

### Therapeutic-principle classification

The three-category therapeutic-principle framework served two distinct purposes. First, it defined the prespecified intervention scope of the review: eligible formulas had to be classifiable as kidney-tonifying alone (PK), kidney-tonifying plus liver-soothing (KL), or kidney-tonifying plus blood-activating (KB). Second, among eligible studies, the same framework was used for exploratory subgroup analyses. The 41 included studies were classified using a hierarchical, rule-based approach. Classification certainty was graded as Level 1 when an explicit therapeutic principle was reported, Level 2 when the treated TCM pattern was clearly described, and Level 3 when classification relied on the predominant actions of the core herbs. The Level 1–3 certainty scheme was introduced to improve classification transparency and support sensitivity analyses; it did not alter the prespecified three-category intervention scope. Two reviewers independently performed the classification, and disagreements were resolved through discussion or consultation with a third reviewer. Final classifications were reached by consensus, and the study-level rationale and certainty for each assignment are reported in Additional file 1: **Supplementary Table S6**. Sensitivity analyses excluding Level 3 assignments are reported in **Supplementary Table S16**. This framework was intended to structure intervention heterogeneity and should not be interpreted as a universally accepted TCM taxonomy, a biomedical classification, or evidence of therapeutic superiority. Herb frequencies and the six high-frequency core herbs selected within each subgroup are reported in Additional file 1: **Supplementary Tables S7** and **S21**.

### Risk of bias assessment

Two reviewers independently assessed the risk of bias using the original Cochrane Risk of Bias tool (RoB 1.0), covering all required procedures, including random sequence generation, allocation concealment, blinding of participants and personnel, blinding of outcome assessment, management of incomplete outcome data, selective reporting, and other bias (13). Disagreements between the reviewers were resolved through discussion or consultation with a third reviewer. Risk-of-bias summary figures were generated using RevMan 5.4.1. RoB 1 was retained because many older trial reports lacked the information required for a more granular assessment; this choice and the predominance of unclear judgments were treated as limitations. Domain-level counts and percentages of low, unclear, and high risk judgments were tabulated for all 41 studies in Additional file 1: **Supplementary Tables S8** and **S9**. Particular attention was paid to allocation concealment and blinding, and these domains were considered when downgrading the certainty of evidence using GRADE. For subjective or composite outcomes, particularly menstrual reporting and overall clinical effectiveness, unclear blinding and selective reporting were considered especially relevant to risk-of-bias and GRADE judgments.

### Statistical analysis

Meta-analyses were performed for outcomes with sufficient extractable data. Continuous outcomes were analyzed using mean differences (MDs) with 95% confidence intervals (CIs). MDs were used for FSH, LH, E2, AMH, AFC, and PSV after unit confirmation or standardization. The AMH and E2 unit-conversion audit and the unit-confirmed sensitivity analyses are reported in Additional file 1: **Supplementary Tables S12** and **S13**. Menstrual return or resumption and overall clinical effectiveness were pooled as ORs, adverse events were pooled primarily as ORs, and a supplementary RD analysis was used to include double-zero adverse-event studies.

End-of-treatment values were used for continuous outcomes because endpoint means and SDs were reported more consistently than paired change SDs or adjusted between-group effects. No change-score SD was derived and no independence assumption was applied because baseline–endpoint correlations, paired change SDs, and adjusted effects were unavailable (14). All endpoint SDs and their source status were documented in Additional file 2. For OR analyses, a continuity correction of 0.5 was applied only to studies with a zero cell. For adverse events, double-zero studies were excluded from the primary OR analysis but included in the supplementary RD analysis; studies without analyzable arm-level event counts were not recoded as zero.

All overall meta-analyses, subgroup analyses, meta-regression analyses, prediction intervals, and forest plots were based on the final cleaned dataset and used a restricted maximum likelihood (REML) random-effects model. Statistical heterogeneity was assessed using Cochran’s Q-test, I^2^, and τ^2^ (15). A 95% prediction interval was calculated for each overall analysis and for subgroup analyses when estimable, to describe the expected range of effects in a future comparable study.

Exploratory therapeutic-principle subgroup analyses were conducted for FSH, LH, and the unit-confirmed E2 dataset. Treatment duration was also evaluated as an exploratory heterogeneity factor. Formal tests for subgroup differences were performed using subgroup Q tests and univariable meta-regression when estimable. Exploratory univariable meta-regression assessed therapeutic principle, treatment duration, mean age, and total sample size; AMH models were supplementary because of the limited study count. All meta-regression analyses used study-level covariates and were interpreted as hypothesis-generating rather than causal or comparative evidence. Missing study-level covariates, such as mean age, were not imputed.

Small-study effects were explored using contour-enhanced funnel plots, Egger regression, and trim-and-fill analyses for outcomes with at least 10 studies, including the final 14-study adverse-event OR dataset (16, 17). These analyses were interpreted as exploratory assessments of small-study effects and were not used to exclude the possibility of publication bias. All meta-analyses were conducted using StataNow/MP 19.5 (StataCorp LLC, College Station, TX, USA).

### Certainty of evidence (GRADE)

The Grading of Recommendations Assessment, Development and Evaluation (GRADE) framework was used to assess certainty across five domains: risk of bias, inconsistency, indirectness, imprecision, and publication bias (18). GRADE assessments covered the patient-important menstrual return or resumption outcome; the surrogate biomarkers FSH and AMH; the secondary outcomes LH, E2, and AFC; the exploratory PSV outcome; adverse events; and overall clinical effectiveness. Overall clinical effectiveness was graded for completeness but remained a supplementary, variably defined composite outcome and was not used as primary clinical-efficacy evidence.

Downgrading decisions were prespecified as follows: risk of bias, when most contributing studies had unclear or high risk in key domains; inconsistency, when the data had substantial heterogeneity (e.g., I^2^ > 50%) and remained unexplained after subgroup or meta-regression analyses; indirectness, when the population, intervention, comparator, or outcomes differed from the review question; imprecision, when CIs were wide or sample sizes were insufficient for precise estimation; publication bias, when funnel plot asymmetry or Egger’s test suggested that small-study effects might exist for outcomes with at least 10 studies. Two reviewers independently rated certainty, and disagreements between them were resolved through discussion or consultation with a third reviewer.

## Results

### Study selection

**Figure 1:**
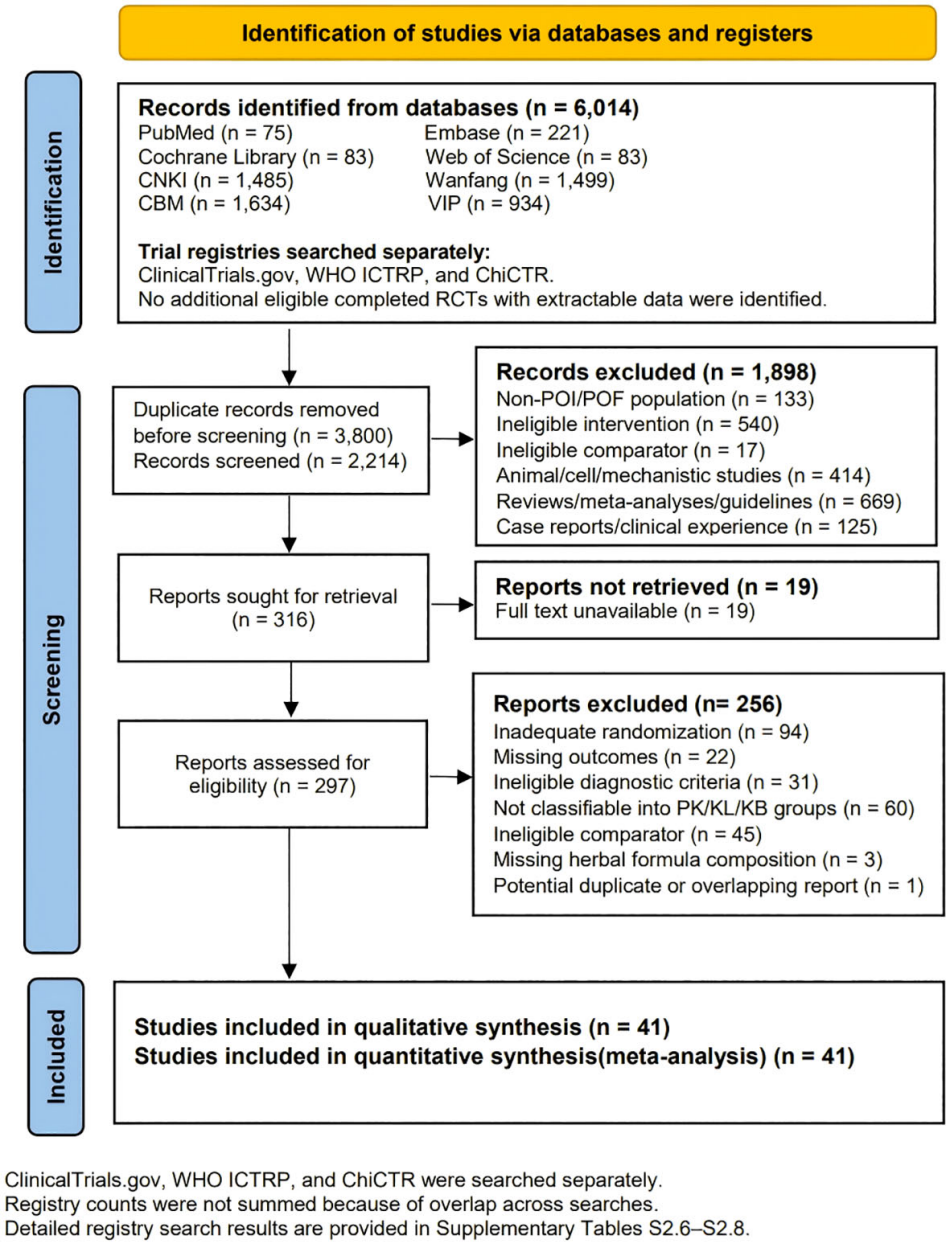
Literature screening process and results (PRISMA 2020 flow diagram). PRISMA 2020 flow diagram showing identification, screening, full-text assessment, exclusion of 256 reports, and inclusion of 41 HRT-controlled RCTs in the qualitative and quantitative syntheses.

A total of 6,014 records were identified from bibliographic databases. Trial registries were searched separately and did not identify additional completed RCTs with publicly extractable outcome data. After removal of 3,800 duplicate records, 2,214 records remained for title and abstract screening. Of these, 1,898 records were excluded, and 316 reports were sought for retrieval. Nineteen reports were not retrieved, leaving 297 reports for full-text eligibility assessment. Of these, 256 were excluded, including 60 because the herbal formula was outside, or could not be reliably classified within, the prespecified PK/KL/KB intervention scope. The remaining 41 HRT-controlled RCTs were included in both the qualitative and quantitative syntheses (19–59). Potentially overlapping reports were handled according to the predefined data-handling rules to avoid double-counting participants in the primary quantitative synthesis. The study selection process is shown in **Figure 1**.

### Study characteristics

The included studies enrolled women diagnosed with POI before the age of 40 years. All eligible interventions were pure orally administered Chinese herbal formulas, including decoctions, granules, capsules, pills, or Chinese patent medicines with clearly reported herbal composition. All 41 included trials used HRT-based comparator regimens without clomiphene-containing active endocrine comparators. Treatment duration was at least three menstrual cycles or three months. Outcome data included strict menstrual return or resumption, FSH, AMH, LH, E2, AFC, exploratory PSV, supplementary overall clinical effectiveness, and adverse events. The 41 studies were classified into PK, KL, or KB categories with study-level certainty recorded as Level 1, 2, or 3. Detailed study and participant characteristics are presented in **Table 1**.

### Risk of bias assessment

**Figure 2:**
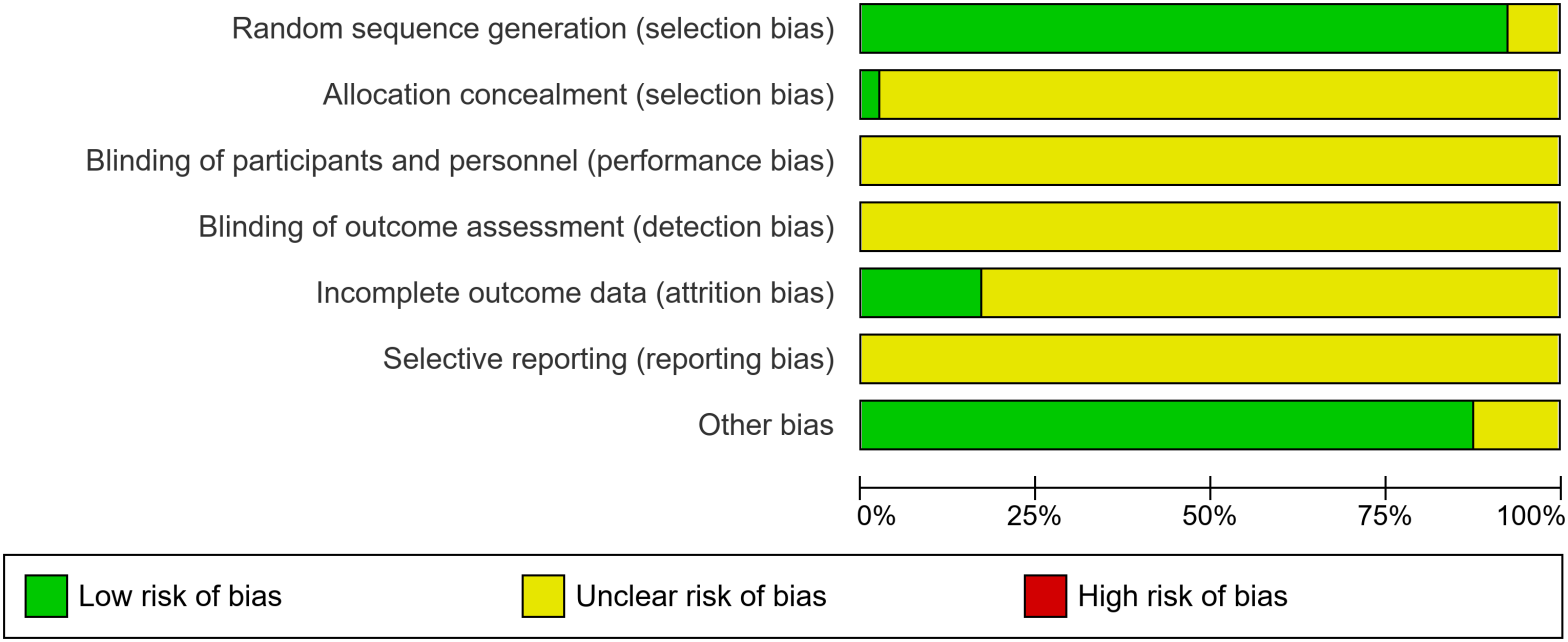
Risk of bias assessment. Risk-of-bias summary for the 41 included RCTs using the Cochrane RoB 1 tool; study-level and domain-level judgments are reported in Additional file 1: **Supplementary Tables S8** and **S9**.

The overall risk-of-bias assessment is summarized in **Figure 2**. Random sequence generation was rated as low risk in 38 of 41 studies, whereas allocation concealment was rated as low risk in only one study and unclear in 40 studies. Blinding of participants and personnel, blinding of outcome assessment, and selective outcome reporting were unclear in all 41 studies. This pattern was considered particularly relevant for menstrual-outcome reporting and the subjective, variably defined overall clinical effectiveness outcome. Incomplete outcome data were rated as low risk in 7 studies and unclear in 34 studies, whereas other bias was rated as low risk in 36 studies and unclear in 5 studies. Full study-level and domain-level judgments are provided in Additional file 1: **Supplementary Tables S8** and **S9**.

### Overall REML meta-analysis

**Figure 3:**
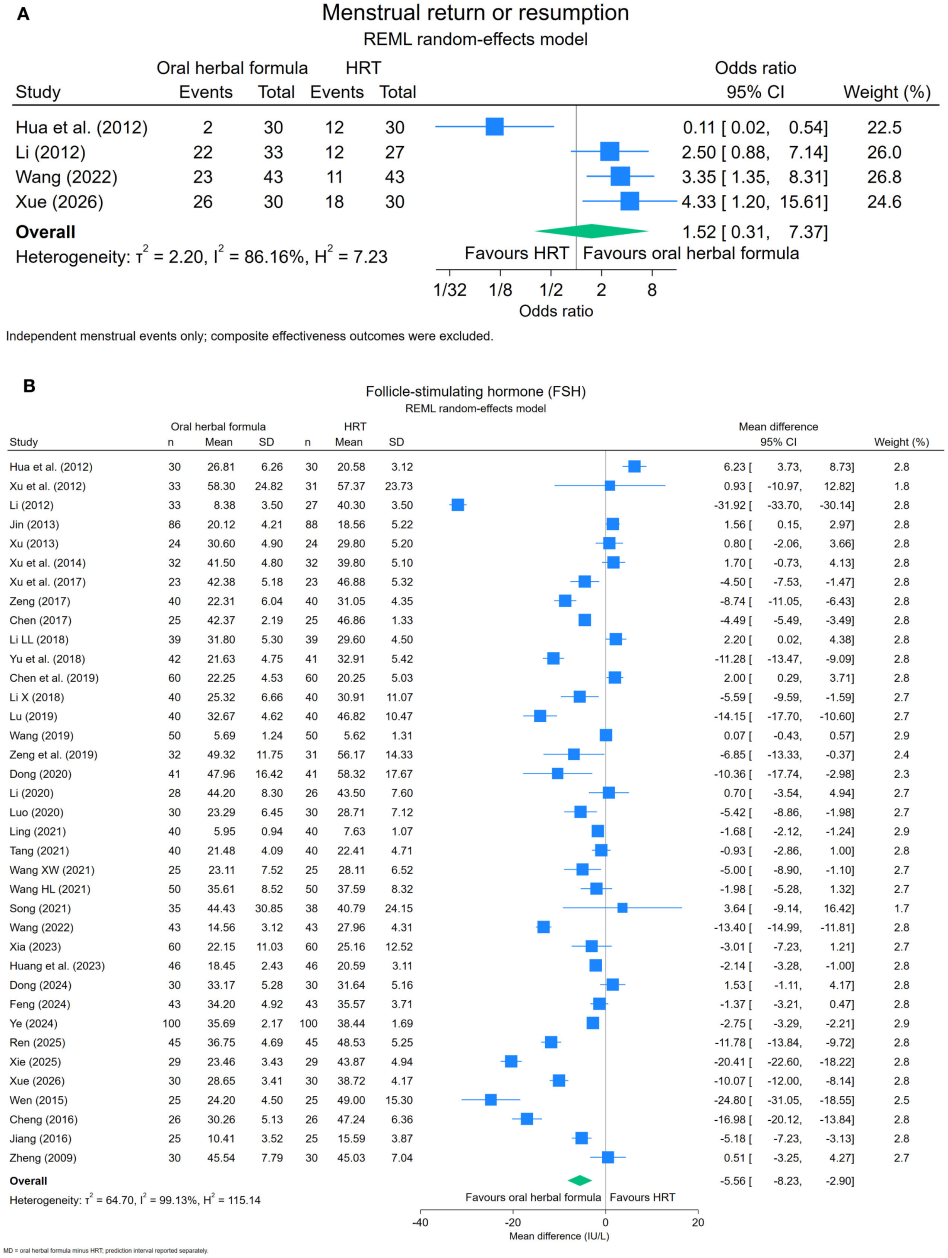
Forest plots for the patient-important menstrual outcome and the most frequently reported primary surrogate biomarker: **(A)** menstrual return or resumption, expressed as odds ratios; and **(B)** FSH, expressed as mean differences. REML random-effects estimates are shown with 95% confidence intervals; the corresponding pooled prediction intervals are reported in **Table 2** and Additional file 1: **Supplementary Table S10**. For menstrual return or resumption, an OR above 1 favors oral herbal formulas; for FSH, a negative MD favors oral herbal formulas. The vertical dashed line indicates the null effect. Complete forest plots for all outcomes, including dichotomous outcomes, are provided in Additional file 1: **Supplementary Figure S1**.

The overall REML random-effects meta-analysis results are summarized in **Table 2**. **Figure 3** presents forest plots for menstrual return or resumption and FSH, which represented the largest primary-surrogate evidence base; the AMH forest plot and complete forest plots for all other outcomes are provided in Additional file 1: **Supplementary Figure S1**. Pooled point estimates favored oral herbal formulas for several surrogate and exploratory outcomes, but the patient-important menstrual outcome showed no clear between-group difference. However, most continuous outcomes showed substantial heterogeneity with wide 95% prediction intervals, indicating uncertainty about the expected effect in future comparable studies.

**Table 2:** Main meta-analysis results, outcome hierarchy, and certainty of evidence.

Outcome	Outcome role	Studies	Participants	Effect measure	Pooled effect	95% CI	95% PI	I^2^	Certainty of evidence	Interpretation
Menstrual return or resumption	Patient/function-related outcome	4	266	OR	1.52	0.31 to 7.37	0.001 to 2166.52	86.16%	Very low	No clear between-group difference was detected; the estimate was highly imprecise and heterogeneous.
FSH	Primary surrogate biomarker	37	2,893	MD, IU/L	-5.56	-8.23 to -2.90	-22.12 to 11.00	99.13%	Very low	Point estimates favored herbal formulas, but FSH is a surrogate biomarker and certainty is very low.
AMH	Primary surrogate biomarker	12	1,198	MD, ng/mL	0.17	0.03 to 0.30	-0.35 to 0.68	98.17%	Very low	Point estimates favored herbal formulas, but AMH is a surrogate biomarker and certainty is very low.
LH	Secondary endocrine surrogate	35	2,753	MD, IU/L	-3.01	-4.66 to -1.35	-12.86 to 6.85	98.11%	Very low	Point estimates favored herbal formulas, but LH is a surrogate biomarker and certainty is very low.
E2	Secondary endocrine surrogate	35	2,693	MD, pmol/L	22.48	6.92 to 38.04	-73.12 to 118.09	99.80%	Very low	Point estimates favored herbal formulas, but E2 is a surrogate biomarker and certainty is very low.
AFC	Secondary ovarian reserve-related surrogate	8	688	MD, follicles	0.46	0.03 to 0.89	-1.08 to 1.99	94.95%	Very low	Point estimates favored herbal formulas, but AFC is a surrogate ovarian reserve-related marker and certainty is very low.
PSV	Exploratory hemodynamic outcome	5	477	MD, cm/s	0.57	0.44 to 0.70	0.37 to 0.78	0.00%	Very low	PSV is an exploratory hemodynamic outcome; evidence is very uncertain.
Adverse events	Safety outcome	14 OR; 22 RD	1,114 OR; 1,755 RD	OR; RD sensitivity	OR 0.20; RD -0.08	OR 0.11 to 0.36; RD -0.13 to -0.03	OR 0.06 to 0.66; RD -0.32 to 0.17	16.67% for OR; 77.45% for RD	Very low	Fewer adverse events were reported in the herbal-formula groups, but safety remains uncertain because ascertainment and reporting were incomplete and inconsistent.

CI, confidence interval; PI, prediction interval; MD, mean difference; OR, odds ratio; FSH, follicle-stimulating hormone; LH, luteinizing hormone; E2, estradiol; AMH, anti-Müllerian hormone; AFC, antral follicle count; PSV, ovarian peak systolic velocity; GRADE, Grading of Recommendations Assessment, Development and Evaluation. I^2^ indicates between-study heterogeneity. The 95% prediction interval reflects the expected range of effects in a future comparable study under the random-effects model. Evidence certainty was assessed using the GRADE approach. For adverse events, the primary OR analysis included 14 non-double-zero studies and 1,114 participants, and the RD sensitivity analysis included 22 studies and 1,755 participants, including eight double-zero studies.

FSH was lower in the herbal-formula groups (37 studies, 2,893 participants; MD −5.563, 95% CI −8.230 to −2.896; I^2^ = 99.13%), but the 95% prediction interval crossed the null (−22.125 to 10.999), indicating substantial between-study variability. AMH was higher in the herbal-formula groups (12 studies, 1,198 participants; MD 0.167, 95% CI 0.035 to 0.299; I^2^ = 98.17%), but its prediction interval also crossed the null (−0.347 to 0.681), and statistical significance was unstable in leave-one-out analyses. For menstrual return or resumption, no clear between-group difference was detected (4 studies, 266 participants; OR 1.523, 95% CI 0.315 to 7.371; I^2^ = 86.16%; 95% prediction interval 0.001 to 2166.521). These findings represent very low-certainty evidence and should not be interpreted as proof of ovarian recovery, fertility benefit, or sustained clinical improvement.

LH was lower (35 studies, 2,753 participants; MD −3.005, 95% CI −4.658 to −1.353; I^2^ = 98.11%) and E2 was higher (35 studies, 2,693 participants; MD 22.483, 95% CI 6.924 to 38.042; I^2^ = 99.80%) in the herbal-formula groups. The 95% prediction intervals crossed the null for both LH (−12.861 to 6.851) and E2 (−73.121 to 118.087), indicating marked uncertainty across future comparable trials.

In the unit-confirmed sensitivity analyses, exclusion of studies with suspected or unverifiable unit assumptions yielded an AMH estimate of MD 0.204 (95% CI 0.031 to 0.377; I^2^ = 98.65%; 11 studies) and an E2 estimate of MD 16.089 (95% CI 4.826 to 27.352; I^2^ = 99.61%; 31 studies). The directions of effect were preserved, but heterogeneity remained extreme, and the statistical significance of the AMH estimate was unstable in several leave-one-out analyses.

AFC was higher in the herbal-formula groups (8 studies, 688 participants; MD 0.457, 95% CI 0.028 to 0.886; I^2^ = 94.95%), and PSV was also higher (5 studies, 477 participants; MD 0.570, 95% CI 0.444 to 0.697; I^2^ = 0.00%). The AFC prediction interval crossed the null (−1.079 to 1.992), whereas the PSV prediction interval did not (0.365 to 0.776). Nevertheless, PSV remained an exploratory ovarian hemodynamic outcome with limited clinical interpretability and was not considered a standard efficacy endpoint for POI.

Overall clinical effectiveness was retained only as a supplementary, variably defined composite outcome (34 studies, 2,807 participants; OR 2.187, 95% CI 1.762 to 2.715; I^2^ = 9.26%) and is reported only in the **Supplementary Material**. The adverse-event OR analysis included 14 non-double-zero studies and 1,114 participants (OR 0.198, 95% CI 0.109 to 0.359; I^2^ = 16.67%), whereas the RD sensitivity analysis included 22 studies and 1,755 participants, including eight double-zero studies (RD −0.077, 95% CI −0.128 to −0.025; I^2^ = 77.45%). The numerical direction favored fewer reported adverse events with oral herbal formulas, but the safety profile remains unestablished because adverse-event ascertainment, severity grading, relatedness assessment, and reporting were incomplete and non-standardized.

### Therapeutic-principle subgroup analysis

Exploratory therapeutic-principle subgroup analyses were conducted for FSH, LH, and unit-confirmed E2, with classification-certainty sensitivity analyses excluding Level 3 assignments. The subgroup and meta-regression results are reported in Additional file 1: **Supplementary Table S11** and **Supplementary Figures S2–S3**; **Table 3** summarizes the key robustness analyses. Subgroup-specific point estimates differed across PK, KL, and KB groups for several outcomes, but the patterns were not interpreted as evidence of therapeutic superiority. Substantial residual heterogeneity remained in several subgroup analyses, and some subgroup estimates were based on a limited number of studies. Therefore, the therapeutic-principle subgroup findings were interpreted as exploratory, hypothesis-generating patterns rather than definitive comparative efficacy evidence among therapeutic principles.

**Table 3:** Key sensitivity and robustness analyses.

Issue addressed	Primary analysis	Sensitivity/robustness analysis	Result	Interpretation	Methodological issue addressed
AMH unit-sensitivity analysis	AMH main analysis, 12 studies.	Excluding Song (2021) because of the suspected or unverifiable AMH unit assumption.	Main AMH MD = 0.17, 95% CI 0.03 to 0.30, I^2^ = 98.17%. Unit-confirmed sensitivity: 11 studies, MD = 0.20, 95% CI 0.03 to 0.38, I^2^ = 98.65%.	The direction remained favorable, but heterogeneity remained extreme; statistical significance changed in several leave-one-out iterations.	AMH unit-conversion uncertainty and influence of individual studies.
E2 unit-sensitivity analysis	E2 main analysis, 35 studies.	Excluding Li (2012), Wen (2015), Yu et al. (2018), and Zeng (2017) because of suspected or unverifiable E2 unit assumptions.	Main E2 MD = 22.48, 95% CI 6.92 to 38.04, I^2^ = 99.80%. Unit-confirmed sensitivity: 31 studies, MD = 16.09, 95% CI 4.83 to 27.35, I^2^ = 99.61%.	The direction remained consistent, but the estimate was attenuated and heterogeneity remained extreme.	E2 unit-conversion uncertainty.
Adverse-event double-zero handling	Adverse-event OR analysis excluding double-zero studies.	Adverse-event RD sensitivity analysis including double-zero studies.	OR analysis: 14 studies and 1,114 participants, OR = 0.20, 95% CI 0.11 to 0.36, I^2^ = 16.67%. RD sensitivity: 22 studies and 1,755 participants, including eight double-zero studies, RD = -0.08, 95% CI -0.13 to -0.03, I^2^ = 77.45%. Xu (2013) was excluded because the herbal-arm total event count was not explicitly reported. Zeng et al. (2019) was included as an explicit double-zero study.	Fewer adverse events were reported in the herbal-formula groups, but safety remains uncertain because adverse-event ascertainment and reporting were incomplete and inconsistent.	Double-zero studies and incomplete adverse-event reporting.
Endpoint-value analysis and change-score feasibility	End-of-treatment values were used for the main meta-analysis.	Baseline and endpoint availability were audited to assess feasibility of change-from-baseline synthesis.	Formal change-from-baseline meta-analysis was not feasible because paired change SDs or adjusted effects were not consistently reported.	Endpoint-value analysis was retained, and this limitation should be explicitly acknowledged.	Concern that endpoint values may not fully account for baseline differences.
Therapeutic-principle subgroup and meta-regression	Therapeutic-principle subgroup analyses were conducted for FSH, LH, and the E2 unit-confirmed dataset.	Univariable meta-regression assessed therapeutic principle, treatment duration, mean age, and total sample size; AMH models were supplementary because of the small number of studies.	Subgroup-difference p values were 0.001 for FSH, 0.065 for LH, and 0.361 for E2. Therapeutic-principle meta-regression showed an exploratory association for FSH (p = 0.039), but not for LH or E2. Treatment duration, mean age, and total sample size did not explain between-study variation.	Only the FSH analyses showed an exploratory signal, while substantial residual heterogeneity remained. These analyses do not establish comparative superiority.	Subjectivity and over-interpretation of therapeutic-principle subgroup analysis.
Sensitivity analysis for therapeutic-principle classification certainty	The main analyses included six formulas assigned Level 3 classification certainty.	Overall pooled analyses were repeated after excluding Level 3 studies and using Level 1 studies only; therapeutic-principle meta-regression was also repeated after excluding Level 3 studies.	After excluding Level 3 studies, the pooled direction remained for FSH (32 studies, MD -5.07, 95% CI -7.84 to -2.30), LH (30 studies, MD -2.31, 95% CI -3.92 to -0.71), and E2 (28 studies, MD 13.95, 95% CI 2.92 to 24.97). In Level 1-only analyses, LH and AMH were no longer statistically significant. Excluding Level 3 studies, therapeutic-principle meta-regression remained significant only for FSH (p = 0.035), not LH (p = 0.374) or E2 (p = 0.498).	The overall direction was generally preserved, but classification-based subgroup signals were not consistently robust and remained exploratory.	Subjectivity and robustness of therapeutic-principle classification.
Small-study effects and trim-and-fill	Contour-enhanced funnel plots were examined for outcomes with at least 10 studies.	Egger regression and trim-and-fill analyses were performed for FSH, LH, E2 unit-confirmed, AMH unit-confirmed, overall clinical effectiveness, and the 14-study adverse-event OR dataset.	Egger p values were 0.938 for FSH, 0.231 for LH, 0.355 for E2, <0.001 for AMH, 0.003 for overall clinical effectiveness, and 0.739 for adverse events. Trim-and-fill imputed no studies for FSH, LH, E2, or AMH. For overall clinical effectiveness, 9 studies were imputed on the left and the adjusted OR was 1.84 (95% CI 1.50 to 2.27). For adverse events, 4 studies were imputed on the right and the adjusted OR was 0.29 (95% CI 0.15 to 0.56).	Possible small-study effects were detected for AMH and overall clinical effectiveness. Egger testing did not indicate small-study effects for adverse events; the trim-and-fill result remains exploratory because safety reporting was incomplete and heterogeneous.	Publication bias and reliance on small positive studies.

**Table 3** summarizes the AMH and E2 unit-confirmed analyses, adverse-event double-zero handling, endpoint-value and change-score feasibility, therapeutic-principle subgroup and classification-certainty analyses, and small-study-effect assessments. Classification levels: Level 1, explicit therapeutic function or principle stated in the original report; Level 2, ambiguous therapeutic function but a clearly specified treated TCM pattern; Level 3, classification based on the predominant actions of core (key) herbs.

### Additional heterogeneity analyses

Subgroup-difference p values were 0.001 for FSH, 0.065 for LH, and 0.361 for unit-confirmed E2. Therapeutic-principle meta-regression showed an exploratory omnibus association for FSH (p = 0.039) but not for LH or E2. Treatment duration, mean age, and total sample size did not meaningfully explain between-study heterogeneity, and residual I^2^ remained extreme. These study-level analyses were exploratory and did not establish comparative superiority among therapeutic-principle categories.

### Small-study effects and publication bias

Small-study effects and potential publication bias were assessed using contour-enhanced funnel plots, Egger regression, and trim-and-fill analyses for outcomes with at least 10 studies. The corresponding plots and numerical results are provided in Additional file 1: **Supplementary Figure S5** and **Supplementary Table S19**. Egger regression indicated possible small-study-effect signals for unit-confirmed AMH (p < 0.001) and overall clinical effectiveness (p = 0.003), but not for FSH, LH, unit-confirmed E2, or adverse events. Trim-and-fill imputed nine studies on the left for overall clinical effectiveness, attenuating the pooled OR to 1.844 (95% CI 1.498 to 2.270), and four studies on the right for adverse events, attenuating the pooled OR to 0.291 (95% CI 0.151 to 0.560). A non-significant Egger test was not interpreted as evidence that publication bias was absent, and the trim-and-fill analyses remained exploratory and were not considered sufficient to exclude publication bias.

### Certainty of evidence

Certainty ratings are summarized in **Table 2**, and the full nine-outcome GRADE evidence profile, including overall clinical effectiveness for completeness, is provided in Additional file 1: **Supplementary Table S20**. All nine outcomes were rated as very low-certainty evidence. Overall clinical effectiveness was not included in the main evidence table because it was a variably defined composite outcome and was assessed only as a supportive outcome in the **Supplementary Material**.

## Discussion

### Principal findings

This systematic review synthesized 41 HRT-controlled RCTs of pure oral Chinese herbal formulas within three prespecified therapeutic-principle categories. Interpretation is constrained because all nine outcomes were rated as very low-certainty evidence and heterogeneity was extreme for most surrogate outcomes. Prediction intervals crossed the null for menstrual return or resumption, FSH, AMH, LH, E2, AFC, and the adverse-event RD analysis, whereas the PSV interval did not; the latter nevertheless remained an indirect exploratory endpoint.

The analysis identified three main findings. First, no clear difference was found for the strict menstrual return or resumption outcome, while point estimates for FSH, AMH, LH, E2, AFC, and PSV favored herbal formulas but represented very-low-certainty surrogate or exploratory evidence. Second, safety findings suggested fewer reported adverse events in the herbal formula groups, but adverse-event reporting was incomplete and inconsistent across trials. The supportive composite outcome of overall clinical effectiveness was not treated as a robust efficacy endpoint because it was variably defined and frequently assessed in unblinded trials. Third, therapeutic-principle subgroup analyses showed non-uniform point-estimate patterns across PK, KL, and KB groups, but these analyses were exploratory and should not be interpreted as evidence that any therapeutic principle is superior. Taken together, these data do not provide an actionable basis for selecting any specific Chinese herbal formula, therapeutic-principle category, or treatment protocol for routine clinical use. Clinicians should continue to follow international guideline-based POI management, including HRT when clinically appropriate.

### Clinical relevance of the selected outcomes

The selected outcomes reflect clinically relevant physiological domains of POI, but many of them are surrogate indicators rather than patient-important outcomes. FSH reflects gonadotropin dysregulation within the hypothalamic-pituitary-ovarian axis, whereas AMH and AFC provide information related to ovarian reserve (60). E2 and LH provide complementary information on ovarian endocrine regulation, and PSV was included as an exploratory hemodynamic indicator. Menstrual return or resumption was prioritized as the most patient-relevant menstrual outcome, but only four studies were available and the estimate was highly imprecise and heterogeneous.

Nevertheless, changes in FSH, AMH, E2, LH, AFC, or PSV should not be equated with sustained recovery of ovarian function, fertility, live birth, long-term endocrine health, quality of life, or prevention of bone and cardiovascular complications. Evidence for such patient-important outcomes remains limited in the included trials. PSV is not a standard POI efficacy endpoint and remains exploratory because the original reports did not consistently specify the measured ovary or use fully comparable Doppler methods. Overall clinical effectiveness is a supplementary, variably defined composite and cannot be interpreted as menstrual recovery, ovarian recovery, or robust clinical benefit because definitions varied and blinding was generally unclear (3, 61). Accordingly, this outcome was not displayed in the main summary figure or main evidence table and is reported only in the **Supplementary Material**.

### Heterogeneity and therapeutic-principle subgroup interpretation

Extreme heterogeneity persisted for most continuous outcomes despite REML models, prediction intervals, therapeutic-principle subgroup analyses, classification-certainty sensitivity analyses, and univariable meta-regression. This residual heterogeneity likely reflects multiple sources, including differences in formula composition, dosage form, daily dose, treatment duration, diagnostic thresholds, baseline ovarian reserve, outcome measurement, and specific HRT comparator regimens. Therefore, the pooled estimates should be interpreted as average effects across heterogeneous trial conditions rather than as precise treatment effects applicable to all clinical settings.

The therapeutic-principle classification was intended to provide a structured and clinically interpretable way to organize intervention heterogeneity. Subgroup analyses suggested that point-estimate patterns differed across PK, KL, and KB groups for selected outcomes. The patterns were not uniform, residual heterogeneity remained extreme, individual meta-regression contrasts were not statistically significant, and Level 1-only analyses did not preserve statistical significance for LH or AMH. Therefore, the therapeutic-principle subgroup findings should be viewed as hypothesis-generating. They may help inform future trial stratification and formula classification, but they do not establish comparative efficacy or therapeutic superiority among PK, KL, and KB.

The primary synthesis was restricted to HRT-controlled trials; placebo-controlled, clomiphene-containing, add-on, and potentially overlapping records were excluded to improve internal comparability. Consequently, the findings apply only to comparisons of herbal monotherapy with HRT-based regimens and do not establish placebo-controlled efficacy or the benefit of adding herbal formulas to HRT. Exploratory meta-regression indicated an omnibus association with therapeutic principle only for FSH, whereas treatment duration, mean age, and total sample size did not meaningfully explain heterogeneity. Because these analyses used study-level covariates and several subgroups contained few studies, they should be interpreted as exploratory rather than confirmatory. Whether oral Chinese herbal formulas provide additional benefit when added to guideline-based HRT, or offer a clinically meaningful option for women who cannot tolerate or decline HRT, remains uncertain and requires specifically designed randomized trials.

### Small-study effects and publication bias

The assessment of small-study effects suggested additional uncertainty. Egger regression indicated possible small-study effects for unit-confirmed AMH and overall clinical effectiveness, but not for FSH, LH, unit-confirmed E2, or adverse events. Trim-and-fill imputed nine studies for overall clinical effectiveness and four for adverse events, attenuating the adjusted ORs to 1.844 and 0.291, respectively; these hypothetical imputations did not replace the main REML estimates. Although no statistical evidence of small-study effects was detected for FSH, LH, E2, or adverse events, non-significant Egger tests do not exclude publication bias. This is particularly important because the evidence base was geographically concentrated, with studies conducted in China, and small positive trials may be more likely to be published (62). These concerns were incorporated into the GRADE assessment and the interpretation of the evidence.

### Limitations and future directions

This review has several limitations. First, allocation concealment was unclear in 40 of 41 studies, blinding of participants and personnel and blinding of outcome assessment were unclear in all 41 studies, selective outcome reporting was unclear in all 41 studies, and incomplete-outcome-data handling was unclear in 34 studies. These limitations were particularly important for menstrual-outcome reporting and the variably defined overall clinical effectiveness outcome. Second, substantial heterogeneity remained for most continuous outcomes, and prediction intervals frequently crossed the null, limiting confidence in the effects expected in future comparable trials. Third, FSH, AMH, LH, E2, AFC, and PSV were surrogate or exploratory outcomes and cannot establish ovarian recovery, fertility restoration, pregnancy, live birth, sustained patient benefit, quality of life, or protection against long-term bone and cardiovascular outcomes.

Fourth, most included trials did not stratify participants according to POI etiology, such as idiopathic, autoimmune, iatrogenic, or genetic POI, and etiology-specific outcome data were unavailable. Therefore, potential variation in treatment effects across etiological subgroups could not be assessed. Fifth, overall clinical effectiveness remained a supplementary, variably defined composite outcome and should not be interpreted as menstrual recovery or primary clinical-efficacy evidence. Sixth, adverse-event evidence was limited by incomplete and non-standardized ascertainment, severity grading, relatedness assessment, follow-up, and reporting; therefore, neither the OR nor the RD analysis can determine comparative safety.

Seventh, the included formulas differed substantially in composition, dosage form, daily dose, and treatment duration. Dose-response analysis was infeasible, and endpoint-value analyses could not be supplemented by formal change-score meta-analysis because paired change SDs, baseline–endpoint correlations, and adjusted effects were unavailable. The AMH and E2 unit-confirmed analyses also retained extreme heterogeneity. Eighth, the evidence base was geographically concentrated in China, which may limit generalizability to other populations and healthcare settings and may increase the risk of publication or language-related bias. Ninth, eligibility was restricted to formulas within the three prespecified therapeutic-principle categories; therefore, the findings should not be generalized to all oral Chinese herbal formulas used for POI. Therapeutic-principle assignment was rule-based but partly interpretive, particularly for Level 3 classifications, and neither subgroup analysis nor meta-regression established comparative efficacy among PK, KL, and KB. Finally, the supplementary network pharmacology analysis was database-derived, highly sensitive to the POI disease-target definition, and lacked experimental validation; it was therefore treated solely as hypothesis-generating context and was not used to support clinical efficacy, therapeutic-principle superiority, or causal or category-specific mechanisms.

Future research should prioritize rigorously designed, prospectively registered, adequately powered, multicenter randomized trials with transparent allocation concealment, appropriate blinding where feasible, standardized diagnostic criteria, detailed formula composition and dosage reporting, and longer follow-up (63). Future trials should distinguish HRT-controlled and placebo-controlled designs clearly, prespecify clinically meaningful outcome sets, and include patient-important outcomes alongside endocrine and ovarian reserve-related markers. Mechanistic studies should be conducted separately or embedded within trials to determine whether exploratory therapeutic-principle hypotheses correspond to reproducible biological differences (3, 64).

## Conclusions

Among oral Chinese herbal formulas within the three prespecified therapeutic-principle categories, no clear advantage over HRT-based regimens was found for menstrual return or resumption. Although several surrogate and exploratory outcomes had directionally favorable point estimates, all assessed outcomes were supported by very low-certainty evidence, most biomarker analyses showed extreme heterogeneity, and prediction intervals commonly crossed the null. The evidence does not establish clinically meaningful benefit, fertility restoration, sustained ovarian recovery, or a safety advantage, and it does not support superiority of any therapeutic-principle category or routine clinical use of a specific formula. HRT should remain guideline-based care when clinically appropriate; adequately powered, prospectively registered multicenter trials using patient-important outcomes and standardized safety reporting are needed.

## Data Availability

The original contributions presented in the study are included in the article/**Supplementary Material**. Further inquiries can be directed to the corresponding author.

## Supplementary material

Additional File 1 Contains the PRISMA checklist, complete database and registry search strategies, eligibility and exclusion audits, study characteristics, therapeutic-principle classification, herb-frequency data, risk-of-bias summaries, meta-analysis and sensitivity results, the full GRADE profile, all supplementary figures, and the complete methods and results of the exploratory network pharmacology analysis in **Supplementary Table S21–S23**. The network pharmacology analysis was not used to support clinical efficacy, therapeutic-principle superiority, or a causal mechanism.

Additional File 2 Provides the structured study-level and arm-level meta-analysis datasets, comparator and overlap audits, adverse-event extraction, unit-conversion and SD-source audits, endpoint/change-score feasibility data, risk-of-bias and GRADE data, final analysis summaries, and Stata command and QC logs.
