# Supplementary material for: Oral Chinese herbal formulas for premature ovarian insufficiency: a systematic review, meta-analysis, and exploratory therapeutic-principle subgroup analysis
Source: Front Endocrinol (Lausanne). 2026 Aug 5;17:1870487. doi: 10.3389/fendo.2026.1870487 (PMC13485792; doi:10.3389/fendo.2026.1870487)
Supplement: Supplementary file 1 [file Table1.docx]

**Contents**

[Table S1. PRISMA 2020 checklist	3](#_Toc7293)

[Table S2. Search strategies for bibliographic databases and trial registries	7](#_Toc26413)

[Table S2.1 Database: PubMed	7](#_Toc24590)

[Table S2.2 Database: Web of Science	7](#_Toc8908)

[Table S2.3 Database: Embase	8](#_Toc27107)

[Table S2.4 Database: Cochrane Library	8](#_Toc24662)

[Table S2.5 Databases: Chinese databases	9](#_Toc27847)

[Table S2.6 ClinicalTrials.gov registry search	10](#_Toc16204)

[Table S2.7 WHO ICTRP registry search	10](#_Toc19109)

[Table S2.8 ChiCTR registry search	11](#_Toc8335)

[Table S3. PICOS framework, outcome hierarchy, abbreviations, and data-handling rules	13](#_Toc29918)

[Table S4. Studies excluded from the primary synthesis because of comparator mismatch or potential overlap	16](#_Toc31742)

[Table S5. Detailed characteristics of included HRT-controlled RCTs	17](#_Toc2025)

[Table S6. Therapeutic-principle classification of included formulas	22](#_Toc14328)

[Table S7. Chinese materia medica names and frequency in the final 41-study dataset	25](#_Toc17734)

[Table S8. Risk-of-bias assessments of included studies using the Cochrane RoB 1 tool	29](#_Toc14874)

[Table S9. Domain-level summary of risk-of-bias judgments	30](#_Toc26071)

[Table S10. Overall REML random-effects meta-analysis results for all quantitative outcomes	31](#_Toc25255)

[Table S11. Exploratory therapeutic-principle subgroup analyses and univariable meta-regression	32](#_Toc20495)

[Table S12. AMH and E2 unit-conversion audit	33](#_Toc28800)

[Table S13. Unit-confirmed sensitivity analyses for E2 and AMH	37](#_Toc22688)

[Table S14. Structured adverse-event reporting summary	37](#_Toc31130)

[Table S15. Supplementary adverse-event risk-difference analysis including double-zero studies	41](#_Toc10635)

[Table S16. Classification-certainty sensitivity analyses	41](#_Toc17484)

[Table S17. Leave-one-out sensitivity analyses for FSH, LH, E2, and AMH	42](#_Toc2751)

[Table S18. Endpoint-value and change-from-baseline data availability audit	43](#_Toc28656)

[Table S19. Small-study-effect assessments and trim-and-fill sensitivity analyses for outcomes with at least 10 studies	43](#_Toc24751)

[Table S20. Full GRADE evidence profile	44](#_Toc4011)

[Supplementary Methods. Exploratory network pharmacology analysis.	48](#_Toc6271)

[Table S21. Six high-frequency core herbs selected within each therapeutic-principle subgroup	49](#_Toc12377)

[Table S22. Summary of target sets and strict POI intersections	50](#_Toc14011)

[Table S23. Sensitivity analysis of target intersections according to the POI disease-target definition	50](#_Toc19182)

[Figure S1. Additional forest plots for outcomes not displayed in the main Figure 3.	51](#_Toc9405)

[Figure S2. Exploratory therapeutic-principle subgroup forest plots.	59](#_Toc28364)

[Figure S3. Unit-confirmed and classification-certainty sensitivity forest plots.	62](#_Toc31262)

[Figure S4. Leave-one-out sensitivity analyses.	67](#_Toc3037)

[Figure S5. Contour-enhanced funnel plots and trim-and-fill sensitivity analyses.	71](#_Toc10991)

# Table S1. PRISMA 2020 checklist

| **Section and Topic** | **Item #** | **Checklist item** | **Location where item is reported** |
| --- | --- | --- | --- |
| **TITLE** | | |  |
| Title | 1 | Identify the report as a systematic review. | Main manuscript: Title. |
| **ABSTRACT** | | |  |
| Abstract | 2 | See the PRISMA 2020 for Abstracts checklist. | Main manuscript: Abstract. |
| **INTRODUCTION** | | |  |
| Rationale | 3 | Describe the rationale for the review in the context of existing knowledge. | Main manuscript: Introduction (rationale). |
| Objectives | 4 | Provide an explicit statement of the objective(s) or question(s) the review addresses. | Main manuscript: Introduction (final paragraph; review objective). |
| **METHODS** | | |  |
| Eligibility criteria | 5 | Specify the inclusion and exclusion criteria for the review and how studies were grouped for the syntheses. | Main manuscript: Methods (eligibility criteria, outcome definitions, intervention classification, and study grouping); Additional file 1: Tables S3-S6. |
| Information sources | 6 | Specify all databases, registers, websites, organisations, reference lists and other sources searched or consulted to identify studies. Specify the date when each source was last searched or consulted. | Main manuscript: Methods (information sources and search strategy); Additional file 1: Table S2. |
| Search strategy | 7 | Present the full search strategies for all databases, registers and websites, including any filters and limits used. | Additional file 1: Table S2. |
| Selection process | 8 | Specify the methods used to decide whether a study met the inclusion criteria of the review, including how many reviewers screened each record and each report retrieved, whether they worked independently, and if applicable, details of automation tools used in the process. | Main manuscript: Methods (study selection). |
| Data collection process | 9 | Specify the methods used to collect data from reports, including how many reviewers collected data from each report, whether they worked independently, any processes for obtaining or confirming data from study investigators, and if applicable, details of automation tools used in the process. | Main manuscript: Methods (data collection and extraction); Additional file 2. |
| Data items | 10a | List and define all outcomes for which data were sought. Specify whether all results that were compatible with each outcome domain in each study were sought (e.g. for all measures, time points, analyses), and if not, the methods used to decide which results to collect. | Main manuscript: Methods (outcome measures); Additional file 1: Table S3. |
|  | 10b | List and define all other variables for which data were sought (e.g. participant and intervention characteristics, funding sources). Describe any assumptions made about any missing or unclear information. | Main manuscript: Methods (data collection, intervention characteristics, and handling of unclear information); Additional file 1: Tables S3 and S5-S6; Additional file 2. |
| Study risk of bias assessment | 11 | Specify the methods used to assess risk of bias in the included studies, including details of the tool(s) used, how many reviewers assessed each study and whether they worked independently, and if applicable, details of automation tools used in the process. | Main manuscript: Methods (risk-of-bias assessment). |
| Effect measures | 12 | Specify for each outcome the effect measure(s) (e.g. risk ratio, mean difference) used in the synthesis or presentation of results. | Main manuscript: Methods (effect measures and statistical analysis). |
| Synthesis methods | 13a | Describe the processes used to decide which studies were eligible for each synthesis (e.g. tabulating the study intervention characteristics and comparing against the planned groups for each synthesis (item #5)). | Main manuscript: Methods (eligibility criteria, intervention classification, and study grouping); Additional file 1: Tables S3-S6. |
|  | 13b | Describe any methods required to prepare the data for presentation or synthesis, such as handling of missing summary statistics, or data conversions. | Main manuscript: Methods (data preparation and statistical analysis); Additional file 1: Tables S12, S13, and S18; Additional file 2. |
|  | 13c | Describe any methods used to tabulate or visually display results of individual studies and syntheses. | Main manuscript: Methods (presentation and synthesis of results) and Results (study selection and synthesis results); Figure 1; Figure 3; Table 2; Additional file 1: Figures S1-S5. |
|  | 13d | Describe any methods used to synthesize results and provide a rationale for the choice(s). If meta-analysis was performed, describe the model(s), method(s) to identify the presence and extent of statistical heterogeneity, and software package(s) used. | Main manuscript: Methods (statistical synthesis and heterogeneity assessment). |
|  | 13e | Describe any methods used to explore possible causes of heterogeneity among study results (e.g. subgroup analysis, meta-regression). | Main manuscript: Methods (therapeutic-principle subgroup analysis and meta-regression); Additional file 1: Table S11 and Figure S2. |
|  | 13f | Describe any sensitivity analyses conducted to assess robustness of the synthesized results. | Main manuscript: Methods and Results (sensitivity analyses); Table 3; Additional file 1: Tables S13, S15-S17, and S19; Figures S3-S5. |
| Reporting bias assessment | 14 | Describe any methods used to assess risk of bias due to missing results in a synthesis (arising from reporting biases). | Main manuscript: Methods and Results (reporting-bias assessment); Additional file 1: Table S19 and Figure S5. |
| Certainty assessment | 15 | Describe any methods used to assess certainty (or confidence) in the body of evidence for an outcome. | Main manuscript: Methods (certainty-of-evidence assessment). |
| **RESULTS** | | |  |
| Study selection | 16a | Describe the results of the search and selection process, from the number of records identified in the search to the number of studies included in the review, ideally using a flow diagram. | Main manuscript: Results (study selection); Figure 1. |
|  | 16b | Cite studies that might appear to meet the inclusion criteria, but which were excluded, and explain why they were excluded. | Main manuscript: Results (study selection and excluded reports); Additional file 1: Table S4. |
| Study characteristics | 17 | Cite each included study and present its characteristics. | Main manuscript: Results (study characteristics); Table 1; Additional file 1: Table S5. |
| Risk of bias in studies | 18 | Present assessments of risk of bias for each included study. | Main manuscript: Results (risk of bias); Figure 2; Additional file 1: Tables S8-S9. |
| Results of individual studies | 19 | For all outcomes, present, for each study: (a) summary statistics for each group (where appropriate) and (b) an effect estimate and its precision (e.g. confidence/credible interval), ideally using structured tables or plots. | Main manuscript: Results (individual-study results); Figure 3; Additional file 1: Figures S1-S3; Additional file 2. |
| Results of syntheses | 20a | For each synthesis, briefly summarise the characteristics and risk of bias among contributing studies. | Main manuscript: Results (study characteristics and risk of bias); Table 1; Figure 2; Additional file 1: Table S5 and Tables S8-S9. |
|  | 20b | Present results of all statistical syntheses conducted. If meta-analysis was done, present for each the summary estimate and its precision (e.g. confidence/credible interval) and measures of statistical heterogeneity. If comparing groups, describe the direction of the effect. | Main manuscript: Results (overall synthesis results); Table 2; Figure 3; Additional file 1: Table S10 and Figure S1. |
|  | 20c | Present results of all investigations of possible causes of heterogeneity among study results. | Main manuscript: Results (subgroup analysis and meta-regression); Table 3; Additional file 1: Table S11 and Figure S2. |
|  | 20d | Present results of all sensitivity analyses conducted to assess the robustness of the synthesized results. | Main manuscript: Results (sensitivity analyses); Table 3; Additional file 1: Tables S13, S15-S17, and S19; Figures S3-S5. |
| Reporting biases | 21 | Present assessments of risk of bias due to missing results (arising from reporting biases) for each synthesis assessed. | Main manuscript: Results (reporting-bias assessment); Additional file 1: Table S19 and Figure S5. |
| Certainty of evidence | 22 | Present assessments of certainty (or confidence) in the body of evidence for each outcome assessed. | Main manuscript: Results (certainty of evidence); Table 2; Additional file 1: Table S20. |
| **DISCUSSION** | | |  |
| Discussion | 23a | Provide a general interpretation of the results in the context of other evidence. | Main manuscript: Discussion (interpretation of the findings in the context of previous evidence). |
|  | 23b | Discuss any limitations of the evidence included in the review. | Main manuscript: Discussion (limitations of the included evidence). |
|  | 23c | Discuss any limitations of the review processes used. | Main manuscript: Discussion (limitations of the review process). |
|  | 23d | Discuss implications of the results for practice, policy, and future research. | Main manuscript: Discussion (implications for practice and future research). |
| **OTHER INFORMATION** | | |  |
| Registration and protocol | 24a | Provide registration information for the review, including register name and registration number, or state that the review was not registered. | Main manuscript: Abstract and Methods (registration and protocol). |
|  | 24b | Indicate where the review protocol can be accessed, or state that a protocol was not prepared. | Main manuscript: Methods (registration and protocol). |
|  | 24c | Describe and explain any amendments to information provided at registration or in the protocol. | Main manuscript: Methods (protocol amendments). |
| Support | 25 | Describe sources of financial or non-financial support for the review, and the role of the funders or sponsors in the review. | Main manuscript: Funding statement. |
| Competing interests | 26 | Declare any competing interests of review authors. | Main manuscript: Conflict of interest statement. |
| Availability of data, code and other materials | 27 | Report which of the following are publicly available and where they can be found: template data collection forms; data extracted from included studies; data used for all analyses; analytic code; any other materials used in the review. | Main manuscript: Data availability statement; Additional files 1 and 2. |

# Table S2. Search strategies for bibliographic databases and trial registries

Bibliographic databases were searched from inception to May 30, 2026. Trial registries were searched or exported on the dates specified in Tables S2.6-S2.8.

## Table S2.1 Database: PubMed

| **ID** | **Search Terms** | **Results** |
| --- | --- | --- |
| #1 | ("Primary Ovarian Insufficiency"[MeSH Terms] OR "Menopause, Premature"[MeSH Terms]) | 5319 |
| #2 | ("premature ovarian insufficiency"[Title/Abstract] OR "premature ovarian insufficiencies"[Title/Abstract] OR "premature ovarian failure"[Title/Abstract] OR "primary ovarian insufficiency"[Title/Abstract] OR "primary ovarian failure"[Title/Abstract] OR "early menopause"[Title/Abstract] OR "premature menopause"[Title/Abstract] OR "hypergonadotropic hypogonadism"[Title/Abstract] OR "hypergonadotropic amenorrhea"[Title/Abstract] OR "ovarian dysgenesis"[Title/Abstract] OR "gonadal dysgenesis"[Title/Abstract] OR (POI[Title/Abstract] AND ovarian[Title/Abstract]) OR (POF[Title/Abstract] AND ovarian[Title/Abstract])) | 12282 |
| #3 | #1 OR #2 | 13516 |
| #4 | ("Medicine, Chinese Traditional"[MeSH Terms] OR "Drugs, Chinese Herbal"[MeSH Terms] OR "Phytotherapy"[MeSH Terms]) | 119569 |
| #5 | ("traditional Chinese medicine"[Title/Abstract] OR "Chinese medicine"[Title/Abstract] OR "Chinese herbal medicine"[Title/Abstract] OR "Chinese herb"[Title/Abstract] OR "Chinese herbs"[Title/Abstract] OR "Chinese herbal formula"[Title/Abstract] OR "Chinese herbal formulas"[Title/Abstract] OR "Chinese herbal formulae"[Title/Abstract] OR "herbal formula"[Title/Abstract] OR "herbal formulas"[Title/Abstract] OR "herbal prescription"[Title/Abstract] OR "herbal prescriptions"[Title/Abstract] OR decoction[Title/Abstract] OR decoctions[Title/Abstract] OR granule[Title/Abstract] OR granules[Title/Abstract] OR capsule[Title/Abstract] OR capsules[Title/Abstract] OR pill[Title/Abstract] OR pills[Title/Abstract] OR "Chinese patent medicine"[Title/Abstract] OR "Chinese patent medicines"[Title/Abstract] OR TCM[Title/Abstract] OR bushen[Title/Abstract] OR "bu shen"[Title/Abstract] OR shugan[Title/Abstract] OR "shu gan"[Title/Abstract] OR huoxue[Title/Abstract] OR "huo xue"[Title/Abstract] OR "tonifying kidney"[Title/Abstract] OR "kidney-tonifying"[Title/Abstract] OR "nourishing kidney"[Title/Abstract] OR "soothing liver"[Title/Abstract] OR "liver-soothing"[Title/Abstract] OR "activating blood"[Title/Abstract] OR "blood-activating"[Title/Abstract] OR "invigorating blood"[Title/Abstract] OR "blood-invigorating"[Title/Abstract] OR zuogui[Title/Abstract] OR "zuo gui"[Title/Abstract] OR yougui[Title/Abstract] OR "you gui"[Title/Abstract] OR erxian[Title/Abstract] OR "er xian"[Title/Abstract] OR kuntai[Title/Abstract] OR "kun tai"[Title/Abstract]) | 311831 |
| #6 | #4 OR #5 | 387364 |
| #7 | ("Randomized Controlled Trial"[Publication Type] OR "Controlled Clinical Trial"[Publication Type] OR "Clinical Trial"[Publication Type] OR "Random Allocation"[MeSH Terms] OR random*[Title/Abstract] OR randomized[Title/Abstract] OR randomised[Title/Abstract] OR randomly[Title/Abstract] OR placebo*[Title/Abstract] OR trial[Title/Abstract] OR trials[Title/Abstract] OR "clinical trial"[Title/Abstract] OR "controlled trial"[Title/Abstract] OR "double blind"[Title/Abstract] OR "single blind"[Title/Abstract]) | 3116977 |
| #8 | #3 AND #6 AND #7 | 92 |
| #9 | #8 NOT (animals[MeSH Terms] NOT humans[MeSH Terms]) | 75 |

## Table S2.2 Database: Web of Science

| **ID** | **Search Terms** | **Results** |
| --- | --- | --- |
| #1 | TS=("premature ovarian insufficiency" OR "premature ovarian insufficiencies" OR "premature ovarian failure" OR "primary ovarian insufficiency" OR "primary ovarian failure" OR "early menopause" OR "premature menopause" OR "hypergonadotropic hypogonadism" OR "hypergonadotropic amenorrhea" OR "ovarian dysgenesis" OR "gonadal dysgenesis" OR (POI AND ovarian) OR (POF AND ovarian)) | 12796 |
| #2 | TS=("traditional Chinese medicine" OR "Chinese medicine" OR "Chinese herbal medicine" OR "Chinese herb*" OR "Chinese herbal formula*" OR "Chinese herbal formulae" OR "herbal formula*" OR "herbal prescription*" OR decoction* OR granule* OR capsule* OR pill* OR "Chinese patent medicine*" OR TCM OR bushen OR "bu shen" OR shugan OR "shu gan" OR huoxue OR "huo xue" OR "tonifying kidney" OR "kidney-tonifying" OR "nourishing kidney" OR "soothing liver" OR "liver-soothing" OR "activating blood" OR "blood-activating" OR "invigorating blood" OR "blood-invigorating" OR zuogui OR "zuo gui" OR yougui OR "you gui" OR erxian OR "er xian" OR kuntai OR "kun tai") | 477365 |
| #3 | TS=("randomized controlled trial*" OR "randomised controlled trial*" OR RCT* OR "controlled clinical trial*" OR "clinical trial*" OR random* OR randomized OR randomised OR randomly OR placebo* OR trial* OR "double blind" OR "single blind") | 4464048 |
| #4 | #1 AND #2 AND #3 | 83 |

## Table S2.3 Database: Embase

| **ID** | **Search Terms** | **Results** |
| --- | --- | --- |
| #1 | 'premature ovarian insufficiency'/exp OR 'premature menopause'/exp OR 'premature ovarian insufficiency':ti,ab,kw OR 'premature ovarian failure':ti,ab,kw OR 'primary ovarian insufficiency':ti,ab,kw OR 'primary ovarian failure':ti,ab,kw OR 'early menopause':ti,ab,kw OR 'premature menopause':ti,ab,kw OR 'hypergonadotropic hypogonadism':ti,ab,kw OR 'hypergonadotropic amenorrhea':ti,ab,kw OR 'ovarian dysgenesis':ti,ab,kw OR 'gonadal dysgenesis':ti,ab,kw OR ('poi':ti,ab,kw AND ovarian:ti,ab,kw) OR ('pof':ti,ab,kw AND ovarian:ti,ab,kw) | 21748 |
| #2 | 'traditional chinese medicine'/exp OR 'chinese herbal medicine'/exp OR 'herbal medicine'/exp OR 'phytotherapy'/exp OR 'traditional chinese medicine':ti,ab,kw OR 'chinese medicine':ti,ab,kw OR 'chinese herbal medicine':ti,ab,kw OR 'chinese herb':ti,ab,kw OR 'chinese herbs':ti,ab,kw OR 'chinese herbal formula':ti,ab,kw OR 'chinese herbal formulas':ti,ab,kw OR 'chinese herbal formulae':ti,ab,kw OR 'herbal formula':ti,ab,kw OR 'herbal formulas':ti,ab,kw OR 'herbal prescription':ti,ab,kw OR decoction*:ti,ab,kw OR granule*:ti,ab,kw OR capsule*:ti,ab,kw OR pill*:ti,ab,kw OR 'chinese patent medicine':ti,ab,kw OR tcm:ti,ab,kw OR bushen:ti,ab,kw OR 'bu shen':ti,ab,kw OR shugan:ti,ab,kw OR 'shu gan':ti,ab,kw OR huoxue:ti,ab,kw OR 'huo xue':ti,ab,kw OR 'tonifying kidney':ti,ab,kw OR 'kidney-tonifying':ti,ab,kw OR 'nourishing kidney':ti,ab,kw OR 'soothing liver':ti,ab,kw OR 'liver-soothing':ti,ab,kw OR 'activating blood':ti,ab,kw OR 'blood-activating':ti,ab,kw OR 'invigorating blood':ti,ab,kw OR 'blood-invigorating':ti,ab,kw OR zuogui:ti,ab,kw OR 'zuo gui':ti,ab,kw OR yougui:ti,ab,kw OR 'you gui':ti,ab,kw OR erxian:ti,ab,kw OR 'er xian':ti,ab,kw OR kuntai:ti,ab,kw OR 'kun tai':ti,ab,kw | 692583 |
| #3 | 'randomized controlled trial'/exp OR 'controlled clinical trial'/exp OR 'clinical trial'/exp OR 'randomization'/exp OR 'random allocation'/exp OR 'placebo'/exp OR random*:ti,ab,kw OR randomized:ti,ab,kw OR randomised:ti,ab,kw OR randomly:ti,ab,kw OR placebo*:ti,ab,kw OR trial*:ti,ab,kw OR 'clinical trial':ti,ab,kw OR 'controlled trial':ti,ab,kw OR 'double blind':ti,ab,kw OR 'single blind':ti,ab,kw | 5573567 |
| #4 | #1 AND #2 AND #3 | 221 |

## Table S2.4 Database: Cochrane Library

| **ID** | **Search Terms** | **Results** |
| --- | --- | --- |
| #1 | MeSH descriptor: [Primary Ovarian Insufficiency] explode all trees | 200 |
| #2 | MeSH descriptor: [Menopause, Premature] explode all trees | 102 |
| #3 | ("premature ovarian insufficiency" OR "premature ovarian insufficiencies" OR "premature ovarian failure" OR "primary ovarian insufficiency" OR "primary ovarian failure" OR "early menopause" OR "premature menopause" OR "hypergonadotropic hypogonadism" OR "hypergonadotropic amenorrhea" OR "ovarian dysgenesis" OR "gonadal dysgenesis" OR (POI AND ovarian) OR (POF AND ovarian)):ti,ab,kw | 709 |
| #4 | #1 OR #2 OR #3 | 828 |
| #5 | MeSH descriptor: [Medicine, Chinese Traditional] explode all trees | 1932 |
| #6 | MeSH descriptor: [Drugs, Chinese Herbal] explode all trees | 4922 |
| #7 | ("traditional Chinese medicine" OR "Chinese medicine" OR "Chinese herbal medicine" OR Chinese NEXT herb* OR Chinese NEXT herbal NEXT formula* OR "Chinese herbal formulae" OR herbal NEXT formula* OR herbal NEXT prescription* OR decoction* OR granule* OR capsule* OR pill* OR Chinese NEXT patent NEXT medicine* OR TCM OR bushen OR "bu shen" OR shugan OR "shu gan" OR huoxue OR "huo xue" OR "tonifying kidney" OR "kidney-tonifying" OR "nourishing kidney" OR "soothing liver" OR "liver-soothing" OR "activating blood" OR "blood-activating" OR "invigorating blood" OR "blood-invigorating" OR zuogui OR "zuo gui" OR yougui OR "you gui" OR erxian OR "er xian" OR kuntai OR "kun tai"):ti,ab,kw | 83305 |
| #8 | #5 OR #6 OR #7 | 83743 |
| #9 | (random* OR randomized OR randomised OR randomly OR placebo* OR trial* OR "clinical trial*" OR "controlled trial*" OR "double blind" OR "single blind"):ti,ab,kw | 1771752 |
| #10 | #4 AND #8 AND #9 | 83 |

## Table S2.5 Databases: Chinese databases

| **ID** | **Search Field** | **Search Terms** | **Results** |
| --- | --- | --- | --- |
| CNKI | 主题 | (早发性卵巢功能不全 OR 原发性卵巢功能不全 OR 卵巢早衰 OR POI OR POF) AND (补肾 OR 疏肝 OR 活血 OR 滋肾 OR 调肝 OR 左归 OR 右归 OR 二仙 OR 坤泰 OR 中药) | 1485 |
| Wanfang | 主题 | (早发性卵巢功能不全 OR 原发性卵巢功能不全 OR 卵巢早衰 OR POI OR POF) AND (补肾 OR 疏肝 OR 活血 OR 滋肾 OR 调肝 OR 左归 OR 右归 OR 二仙 OR 坤泰 OR 中药) | 1499 |
| VIP | M | (早发性卵巢功能不全 OR 原发性卵巢功能不全 OR 卵巢早衰 OR POI OR POF) AND (补肾 OR 疏肝 OR 活血 OR 滋肾 OR 调肝 OR 左归 OR 右归 OR 二仙 OR 坤泰 OR 中药) | 934 |
| CBM | 常用字段 | (早发性卵巢功能不全 OR 原发性卵巢功能不全 OR 卵巢早衰 OR POI OR POF) AND (补肾 OR 疏肝 OR 活血 OR 滋肾 OR 调肝 OR 左归 OR 右归 OR 二仙 OR 坤泰 OR 中药) | 1634 |

For Chinese databases, no randomized-trial filter was applied to maximize sensitivity; randomized controlled trial eligibility was assessed during screening.

## Table S2.6 ClinicalTrials.gov registry search

| **ID** | **Search terms / filter** | **Results** | **Screening and exclusion notes** |
| --- | --- | --- | --- |
| #1 | "premature ovarian insufficiency" OR "premature ovarian failure" OR "primary ovarian insufficiency" Filter: Interventional Search date: 2026-05-30 | 133 search hits | The search retrieved 133 records. After title/condition/intervention-level screening, three potentially relevant registry records were exported for detailed PICOS assessment. All three were also captured in WHO ICTRP. After PICOS/results screening, two records were excluded and one potentially eligible record was retained only as a qualitative registry summary because no public extractable results were available. No additional RCT was added to the meta-analysis. |

## Table S2.7 WHO ICTRP registry search

| **ID** | **Search terms** | **Results** | **Screening and exclusion notes** |
| --- | --- | --- | --- |
| #1 | "premature ovarian insufficiency" | 178 trials / 183 records | ICTRP search-term counts were not summed because overlapping records were expected across search terms. |
| #2 | "primary ovarian insufficiency" | 68 trials / 68 records | ICTRP search-term counts were not summed because overlapping records were expected across search terms. |
| #3 | "premature ovarian failure" | 147 trials / 150 records | ICTRP search-term counts were not summed because overlapping records were expected across search terms. |
| #4 | "ovarian insufficiency" | 264 trials / 269 records | ICTRP search-term counts were not summed because overlapping records were expected across search terms. |
| #5 | Deduplication and screening of exported registry records Search/export dates:  2026-05-30 | 49 raw exported records → 35 unique TrialID records → 30 linked registry trial groups | Among 30 linked trial groups, 14 were retained only as qualitative registry summaries due to no public extractable results, 14 were excluded after PICOS screening, and 2 were excluded from quantitative synthesis. No additional completed RCT with extractable public outcome data was identified. |

## Table S2.8 ChiCTR registry search

| **ID** | **Search terms** | **Results** | **Screening and exclusion notes** |
| --- | --- | --- | --- |
| #1 | 早发性卵巢功能不全 Search field/filter: Disease term Search date: 2026-05-30 | 50 | These ChiCTR search results were combined for manual screening. Fourteen candidate records were screened; nine overlapped with WHO ICTRP and five were ChiCTR-only independent records. No additional RCT with public extractable data was added to the meta-analysis. |
| #2 | 早发性卵巢衰竭 Search field/filter: Disease term Search date: 2026-05-30 | 0 |  |
| #3 | 卵巢早衰 Search field/filter: Disease term Search date: 2026-05-30 | 13 |  |
| #4 | 原发性卵巢功能不全 Search field/filter: Disease term Search date: 2026-05-30 | 1 |  |
| #5 | 原发性卵巢功能衰竭 Search field/filter: Disease term Search date: 2026-05-30 | 0 |  |
| #6 | premature ovarian insufficiency Search field/filter: English disease term Search date: 2026-05-30 | 14 |  |
| #7 | premature ovarian failure Search field/filter: English disease term Search date: 2026-05-30 | 4 |  |
| #8 | primary ovarian insufficiency Search field/filter: English disease term Search date: 2026-05-30 | 1 |  |
| #9 | primary ovarian failure Search field/filter: English disease term Search date: 2026-05-30 | 0 |  |
| #10 | POI ovarian / POF ovarian / POI ovary / POF ovary Search field/filter: Abbreviation terms Search date: 2026-05-30 | 0 |  |
| #11 | 卵巢功能不全 中药 / 卵巢功能减退 中药 / 卵巢储备功能减退 中药 / 卵巢储备下降 中药 / 卵巢低反应 中药 Search field/filter: Chinese disease + CHM terms Search date: 2026-05-30 | 0 |  |
| #12 | ovarian insufficiency Chinese herbal medicine / ovarian failure Chinese herbal medicine / diminished ovarian reserve Chinese herbal medicine / poor ovarian response Chinese herbal medicine Search field/filter: English disease + CHM terms Search date: 2026-05-30 | 0 |  |
| #13 | Combined manual screening summary | 14 candidate records screened | Final screening decisions: 7 excluded after PICOS screening, 3 excluded from quantitative synthesis, and 4 retained only as qualitative registry summaries because no public extractable results were available. |

# Table S3. PICOS framework, outcome hierarchy, abbreviations, and data-handling rules

Only terms used in the HRT-controlled synthesis and associated supplementary analyses are listed.

| **Abbreviation / term** | **Full name** | **Definition / use in this review** |
| --- | --- | --- |
| A. PICOS framework and synthesis scope | A. PICOS framework and synthesis scope | A. PICOS framework and synthesis scope |
| PICOS | Population, Intervention, Comparator, Outcomes, and Study design | Framework used to define eligibility and organize the synthesis. In the primary synthesis, the comparator domain was restricted to HRT-based treatment. |
| POI | Premature ovarian insufficiency | A condition of impaired ovarian function before 40 years of age, characterized by menstrual disturbance, elevated gonadotropins, and reduced ovarian reserve-related markers. |
| POF | Premature ovarian failure | An earlier term historically used for ovarian insufficiency before 40 years of age. Trials using POF terminology were retained when participant characteristics were consistent with POI/ovarian insufficiency. |
| CHM | Chinese herbal medicine | In this review, CHM refers to orally administered Chinese herbal formulas with reported composition. |
| TCM | Traditional Chinese medicine | The traditional medicine framework used to describe herbal formulas, therapeutic principles, and treated patterns. |
| HRT | Hormone replacement therapy | Hormone-based comparator treatment used in the primary synthesis. Eligible comparators included HRT-based regimens without clomiphene-containing active endocrine comparators. |
| RCT | Randomized controlled trial | A parallel-group randomized clinical study meeting the predefined eligibility criteria. |
| HRT-controlled synthesis | Pure oral Chinese herbal formulas versus HRT-based treatment | The primary quantitative synthesis. Placebo-controlled, clomiphene-containing comparator, add-on, and overlapping records were excluded from the primary synthesis. |
| B. Therapeutic-principle subgroup classification | B. Therapeutic-principle subgroup classification | B. Therapeutic-principle subgroup classification |
| PK | Pure kidney-tonifying | Oral Chinese herbal formulas classified as Bu Shen alone, without liver-soothing or blood-activating therapeutic principles. |
| KL | Kidney-tonifying plus liver-soothing | Formulas combining kidney-tonifying (Bu Shen) and liver-soothing (Shu Gan) therapeutic principles. |
| KB | Kidney-tonifying plus blood-activating | Formulas combining kidney-tonifying (Bu Shen) and blood-activating (Huo Xue) therapeutic principles. |
| Bu Shen | Kidney tonifying | A TCM therapeutic principle aimed at tonifying the kidney system, which is traditionally associated with reproductive regulation. |
| Shu Gan | Liver soothing | A TCM therapeutic principle aimed at regulating liver qi and relieving constraint. |
| Huo Xue | Blood activating | A TCM therapeutic principle aimed at promoting blood circulation and resolving blood stasis. |
| Level 1 | High-certainty therapeutic-principle classification | Explicit therapeutic function or principle was stated in the original report. |
| Level 2 | Moderate-certainty therapeutic-principle classification | Therapeutic function was not fully explicit, but the treated TCM pattern was clearly specified. |
| Level 3 | Lower-certainty therapeutic-principle classification | Classification was based on the predominant actions of core herbs. Level 3 records were excluded in the lower-certainty classification sensitivity analysis. |
| C. Outcome hierarchy and outcome definitions | C. Outcome hierarchy and outcome definitions | C. Outcome hierarchy and outcome definitions |
| FSH | Follicle-stimulating hormone | Primary surrogate biomarker. Lower end-of-treatment values favored the herbal formula group, but FSH was interpreted as a surrogate endocrine marker rather than direct evidence of sustained ovarian recovery. |
| AMH | Anti-Müllerian hormone | Primary surrogate biomarker related to ovarian reserve. AMH was interpreted as a reserve-related biomarker and not as direct evidence of fertility restoration. |
| Menstrual return or resumption | Directly reported return or resumption of menstruation under the prespecified strict outcome definition | Patient-important outcome. Composite “menstrual improvement” definitions were not pooled as the main menstrual outcome. |
| LH | Luteinizing hormone | Secondary endocrine surrogate outcome. Lower end-of-treatment values favored the herbal formula group. |
| E2 | Estradiol | Secondary endocrine surrogate outcome. Higher end-of-treatment values favored the herbal formula group after unit standardization. |
| AFC | Antral follicle count | Secondary ovarian reserve-related ultrasound outcome, reflecting the number of antral follicles detected by ultrasound. |
| PSV | Ovarian arterial peak systolic velocity | Exploratory hemodynamic outcome referring to ovarian arterial blood-flow peak systolic velocity; not treated as a standard efficacy endpoint for POI. |
| AE / AEs | Adverse event / adverse events | Safety outcome referring to reported undesirable events during treatment. AE findings were interpreted cautiously because definitions, ascertainment, severity grading, relatedness, and laboratory monitoring were inconsistently reported. |
| Overall clinical effectiveness | Study-defined overall clinical effectiveness rate | Supplementary composite outcome commonly reported in Chinese trials. Definitions varied and often combined symptoms, menstrual changes, and laboratory indicators; it was not used to support the main efficacy conclusion. |
| D. Statistical and methodological terms | D. Statistical and methodological terms | D. Statistical and methodological terms |
| RoB 1 | Cochrane Risk of Bias 1 tool | Tool used for study-level risk-of-bias assessment across domains including random sequence generation, allocation concealment, blinding, incomplete outcome data, selective reporting, and other bias. |
| GRADE | Grading of Recommendations Assessment, Development and Evaluation | Framework used to assess certainty of evidence across risk of bias, inconsistency, indirectness, imprecision, and publication bias. |
| MD | Mean difference | Effect measure used for continuous outcomes after unit standardization, including FSH, LH, E2, AMH, AFC, and PSV. |
| OR | Odds ratio | Effect measure used for dichotomous outcomes, including menstrual return or resumption, overall clinical effectiveness, and the primary adverse-event OR analysis. |
| RD | Risk difference | Effect measure used in the supplementary adverse-event sensitivity analysis including double-zero studies. |
| CI | Confidence interval | Interval estimate used to express statistical uncertainty around pooled effect estimates. |
| PI | Prediction interval | Interval describing the expected range of effects in a future comparable study; used to support cautious interpretation when heterogeneity was substantial. |
| SD | Standard deviation | Measure of dispersion used for continuous outcome analysis. All endpoint SDs used in the quantitative synthesis were directly reported in the original trial reports. No SD was derived from other summary statistics. The source status of each endpoint SD is documented in Additional file 2. |
| I² | I-squared | Statistic quantifying the proportion of total variability attributable to between-study heterogeneity rather than chance. |
| τ² | Tau-squared | Estimated between-study variance in random-effects meta-analysis. |
| Q | Cochran's Q statistic | Test statistic used to assess heterogeneity across studies. |
| Qb | Between-subgroup Q statistic | Statistic used for subgroup-difference tests in exploratory therapeutic-principle subgroup analyses. |
| k | Number of studies | Number of studies contributing to a given outcome, subgroup, or sensitivity analysis. |
| REML | Restricted maximum likelihood | Estimation method used for all random-effects meta-analyses, subgroup analyses, sensitivity analyses, and meta-regression models. |
| Egger test | Regression-based test for small-study effects | Exploratory test used for outcomes with a sufficient number of studies; a significant result was interpreted as a potential small-study effect rather than definitive publication bias. |
| Trim-and-fill | Nonparametric trim-and-fill analysis | Exploratory small-study/publication-bias sensitivity analysis estimating potentially missing studies and recalculating the pooled effect. |
| Double-zero study | Study with zero events in both arms | For adverse events, double-zero studies were excluded from the primary OR analysis but included in the supplementary RD sensitivity analysis. |
| Endpoint value | End-of-treatment value | Primary continuous-data metric used in the revised quantitative synthesis because endpoint values were reported more consistently than change scores. |
| Change-from-baseline | Within-arm change from baseline to endpoint | Audited for feasibility but not used in formal meta-analysis because paired change SDs or adjusted effects were not consistently reported. |
| E. Network pharmacology-related abbreviations | E. Network pharmacology-related abbreviations | E. Network pharmacology-related abbreviations |
| TCMSP | Traditional Chinese Medicine Systems Pharmacology database | Database used to obtain candidate compounds and compound-target information for exploratory network pharmacology analyses. |
| OB | Oral bioavailability | Screening parameter used in TCMSP to identify candidate active compounds, with a prespecified threshold of OB ≥ 30%. |
| DL | Drug-likeness | Screening parameter used in TCMSP to identify candidate active compounds, with a prespecified threshold of DL ≥ 0.18. Here, DL refers only to drug-likeness. |
| UniProt | Universal Protein Resource | Resource used to map and standardize candidate compound-associated targets to reviewed human proteins and official human gene symbols. |
| GeneCards | GeneCards Human Gene Database | One of the three disease–gene databases used to retrieve POI/POF-associated genes. GeneCards-only targets with relevance scores at or above the dataset-specific 95th percentile were included only in the broader POI sensitivity set. |
| DisGeNET | Disease–gene association database | One of the three independent databases used to retrieve POI/POF-associated genes for construction of the disease-target sets. |
| OMIM | Online Mendelian Inheritance in Man | One of the three disease databases used to identify genes associated with POI/POF. Genes with explicit POI/POF evidence in OMIM were eligible for inclusion in the strict POI core set. |
| Raw standardized POI union | Unfiltered standardized POI/POF-associated gene union | The standardized union of 8,865 POI/POF-associated genes retrieved from GeneCards, DisGeNET, and OMIM before evidence filtering. This set was used for descriptive audit only. |
| Strict POI core set | Evidence-filtered POI disease-target set | The main disease-target set comprising 105 genes supported by at least two of GeneCards, DisGeNET, and OMIM, or by explicit POI/POF evidence in OMIM. |
| POI sensitivity set | Broader POI disease-target sensitivity set | A set of 486 genes comprising the strict POI core set plus single-database GeneCards targets with relevance scores at or above the dataset-specific 95th percentile (Q95 = 31.896). It was used to assess dependence of the results on the POI disease-target definition. |
| Jaccard similarity | Intersection-over-union similarity coefficient | Similarity measure calculated as the number of shared targets divided by the total number of unique targets in the two compared sets. It was used to compare strict-core and sensitivity-set intersections; lower values indicated greater dependence on the disease-target definition. |

Notes: Restricted maximum likelihood (REML) was used for all random-effects meta-analyses, subgroup analyses, sensitivity analyses, and meta-regression models. Drug-likeness (DL) in the network pharmacology section is unrelated to the meta-analysis estimator. The supplementary network pharmacology analysis was limited to representative-herb selection, compound–target retrieval, disease-target-set construction, target-intersection analysis, and disease-target-definition sensitivity analysis. No PPI, GO, KEGG, hub-gene, or enrichment analysis was used to support the final clinical interpretation.

# Table S4. Studies excluded from the primary synthesis because of comparator mismatch or potential overlap

| **Study** | **Year** | **Original study label / Article** | **Reason for exclusion** | **Comparator or overlap issue** | **Action in the primary synthesis** | **Notes** |
| --- | --- | --- | --- | --- | --- | --- |
| Cao et al. (2018) | 2018 | Cao et al. (2018) | Placebo-controlled trial; comparator was placebo capsule rather than HRT-based treatment. | Placebo capsule | Excluded from revised primary HRT-controlled synthesis. | Placebo-controlled comparator mismatch. |
| Cao (2018) | 2018 | Cao (2018) | Placebo-controlled trial; comparator was placebo capsule rather than HRT-based treatment. | Placebo capsule | Excluded from revised primary HRT-controlled synthesis. | Placebo-controlled comparator mismatch. |
| Cao et al. (2025) | 2025 | Cao et al. (2025) | Placebo-controlled trial; comparator was placebo capsule rather than HRT-based treatment. | Placebo capsule | Excluded from revised primary HRT-controlled synthesis. | Placebo-controlled comparator mismatch. |
| Wu (2016) | 2016 | Wu (2016) | Placebo-controlled trial; comparator was placebo rather than HRT-based treatment. | Placebo | Excluded from revised primary HRT-controlled synthesis. | Placebo-controlled comparator mismatch. |
| Ma (2015) | 2015 | Ma (2015) | Comparator included estradiol valerate tablets + clomiphene + progesterone capsules; clomiphene-containing active endocrine comparator was not considered equivalent to HRT-based comparator. | Estradiol valerate + clomiphene + progesterone | Excluded from revised primary HRT-controlled synthesis. | Clomiphene-containing comparator mismatch. |
| Ma (2019) | 2019 | Ma (2019) | Comparator included estradiol valerate tablets + clomiphene + progesterone capsules; clomiphene-containing active endocrine comparator was not considered equivalent to HRT-based comparator. | Estradiol valerate + clomiphene + progesterone | Excluded from revised primary HRT-controlled synthesis. | Clomiphene-containing comparator mismatch. |
| Lu (2018) | 2018 | Lu (2018) | Potential overlap with Lu (2019), which was retained as the larger or more complete report. | Potential duplicate/overlapping study population or report | Excluded from primary synthesis; Lu (2019) retained in the final 41-study dataset. | Potential overlap with Lu (2019); the larger or more complete report was retained. |

The primary synthesis was restricted to pure oral Chinese herbal formulas versus HRT-based comparators to improve internal comparability. Excluding placebo-controlled, clomiphene-containing comparator, and potentially overlapping records improved internal consistency but reduced comparator breadth.

# Table S5. Detailed characteristics of included HRT-controlled RCTs

Part A. Participant and study characteristics (ordered as in the final forest plots)

| **Study (final forest-plot label)** | **Year** | **Diagnosis** | **Sample size, treatment** | **Sample size, control** | **Total sample size** | **Mean age, treatment** | **Mean age, control** |
| --- | --- | --- | --- | --- | --- | --- | --- |
| Hua et al. (2012) | 2012 | POF | 30 | 30 | 60 | 32.74 ± 6.43 | 31.65 ± 5.88 |
| Xu et al. (2012) | 2012 | POF | 33 | 31 | 64 | 36.7 ± 5.2 | 36.5 ± 5.1 |
| Li (2012) | 2012 | POF | 33 | 27 | 60 | 30±5.41 | 30±5.41 |
| Jin (2013) | 2013 | POF | 86 | 88 | 174 | 33.25 ± 3.11 | 34.11 ± 3.31 |
| Xu (2013) | 2013 | POF | 24 | 24 | 48 | 31.8 ± 3.6 | 31.6 ± 3.7 |
| Xu et al. (2014) | 2014 | POF | 32 | 32 | 64 | 36.5 ± 2.8 | 36.0 ± 2.7 |
| Xu et al. (2017) | 2017 | POF | 23 | 23 | 46 | 38.04±1.11 | 37.78±1.09 |
| Zeng (2017) | 2017 | POF | 40 | 40 | 80 | 32.4 ± 2.6 | 32.4 ± 2.6 |
| Chen (2017) | 2017 | POF | 25 | 25 | 50 | 34.54±3.24 | 35.02±2.54 |
| Li LL (2018) | 2018 | POF | 39 | 39 | 78 | 31.28 ± 3.53 | 32.02 ± 3.47 |
| Yu et al. (2018) | 2018 | POI | 42 | 41 | 83 | 31.02 ± 2.34 | 30.97 ± 2.31 |
| Chen et al. (2019) | 2019 | POF | 60 | 60 | 120 | NR | NR |
| Li X (2018) | 2018 | POI | 40 | 40 | 80 | 31.81 ± 4.29 | 31.78 ± 4.26 |
| Lu (2019) | 2019 | POF | 40 | 40 | 80 | NR | NR |
| Wang (2019) | 2019 | POF | 50 | 50 | 100 | 35.11±3.99 | 34.87±4.23 |
| Yuan (2019) | 2019 | POI | 45 | 45 | 90 | 33.18 ± 4.46 | 32.46 ± 5.45 |
| Zeng et al. (2019) | 2019 | POF | 32 | 31 | 63 | 28.63 ± 6.49 | 31.06 ± 6.61 |
| Dong (2020) | 2020 | POF | 41 | 41 | 82 | 30.97 ± 4.49 | 30.97 ± 5.83 |
| Li (2020) | 2020 | POF | 28 | 26 | 54 | 32.5 ± 3.8 | 35.4 ± 5.2 |
| Luo (2020) | 2020 | POI | 30 | 30 | 60 | 35.07 ± 2.09 | 35.27 ± 3.25 |
| Ling (2021) | 2021 | POI | 40 | 40 | 80 | 32.45 ± 2.16 | 32.10 ± 2.23 |
| Tang (2021) | 2021 | POI | 40 | 40 | 80 | 31.63 ± 3.46 | 32.57 ± 3.60 |
| Wang XW (2021) | 2021 | POI | 25 | 25 | 50 | 36.4 ± 1.1 | 36.5 ± 1.0 |
| Wang HL (2021) | 2021 | POF | 50 | 50 | 100 | 33.96±2.98 | 33.57±4.01 |
| Sui (2021) | 2021 | POI | 60 | 62 | 122 | NR | NR |
| Song (2021) | 2021 | POF | 35 | 38 | 73 | 35.48±0.93 | 37.23±0.61 |
| Wang (2022) | 2022 | POF | 43 | 43 | 86 | 35.65 ± 3.75 | 35.12 ± 3.42 |
| Xia (2023) | 2023 | POI | 60 | 60 | 120 | 30.40 ± 3.74 | 30.83 ± 2.95 |
| Huang et al. (2023) | 2023 | POF | 46 | 46 | 92 | 32.56 ± 2.14 | 32.17 ± 2.33 |
| Dong (2024) | 2024 | POF | 30 | 30 | 60 | 31.26 ± 3.48 | 31.57 ± 3.66 |
| Feng (2024) | 2024 | POF | 43 | 43 | 86 | 34.09±3.54 | 33.35±3.17 |
| Ye (2024) | 2024 | POF | 100 | 100 | 200 | 29.13 ± 1.37 | 29.13 ± 1.37 |
| Wang (2024) | 2024 | POI | 61 | 62 | 123 | 32.26±0.54 | 34.45±0.50 |
| Ren (2025) | 2025 | POF | 45 | 45 | 90 | 31.13 ± 4.55 | 30.76 ± 4.65 |
| Xie (2025) | 2025 | POF | 29 | 29 | 58 | 32.3 ± 3.3 | 33.8 ± 3.5 |
| Zhou et al. (2025) | 2025 | POF | 35 | 35 | 70 | 34.09±2.59 | 34.34±2.66 |
| Xue (2026) | 2026 | POF | 30 | 30 | 60 | 32.5±4.1 | 32.5±4.1 |
| Wen (2015) | 2015 | POF | 25 | 25 | 50 | 34.20 ± 4.10 | 33.99 ± 3.70 |
| Cheng (2016) | 2016 | POF | 26 | 26 | 52 | 33.4 ± 3.7 | 34.2 ± 4.3 |
| Jiang (2016) | 2016 | POF | 25 | 25 | 50 | 34.75±3.12 | 35.01±3.22 |
| Zheng (2009) | 2009 | POF | 30 | 30 | 60 | 36.43±4.17 | 35.78±4.81 |

**Part B. Intervention, comparator, and analyzed outcomes**

| **Study (final forest-plot label)** | **Intervention formula** | **Comparator** | **Treatment duration** | **Outcomes included in the final figure set and quantitative synthesis** | **Notes** |
| --- | --- | --- | --- | --- | --- |
| Hua et al. (2012) | Bu-Shui-Rou-Mu Formula | estrogen tablets + medroxyprogesterone acetate tablets | 12 weeks | Menstrual return/resumption; FSH; LH; E2 |  |
| Xu et al. (2012) | Modified Yi-Jing Decoction | estradiol valerate tablets + medroxyprogesterone acetate | 16 weeks | Menstrual return/resumption (sensitivity only); FSH; LH; E2; Overall clinical effectiveness (supplementary composite) | The menstrual-return record was included only in the prespecified sensitivity analysis using a transparent derived definition. |
| Li (2012) | Yi-Jing Decoction | estradiol valerate tablets + medroxyprogesterone acetate | 12 weeks | Menstrual return/resumption; FSH; E2; Overall clinical effectiveness (supplementary composite) |  |
| Jin (2013) | Kidney-tonifying and Blood-activating Formula | estrogen tablets + medroxyprogesterone acetate tablets | 24 weeks | FSH; LH; E2; AFC; Adverse events (RD sensitivity only; double-zero); Overall clinical effectiveness (supplementary composite) |  |
| Xu (2013) | Kidney-tonifying Menstruation-regulating Formula | estrogen tablets + medroxyprogesterone acetate tablets | 12 weeks | FSH; LH; E2; Overall clinical effectiveness (supplementary composite) | Adverse events were reported, but the herbal-arm total event count was not explicitly available; this study was excluded from the quantitative OR and RD analyses. |
| Xu et al. (2014) | Kidney-tonifying Menstruation-regulating Formula | estrogen tablets + medroxyprogesterone acetate tablets | 24 weeks | FSH; LH; E2; Adverse events (OR; also RD sensitivity); Overall clinical effectiveness (supplementary composite) |  |
| Xu et al. (2017) | Kidney-tonifying Menstruation-regulating Ointment | estrogen + medroxyprogesterone acetate | 12 weeks | FSH; LH; E2; Adverse events (OR; also RD sensitivity); Overall clinical effectiveness (supplementary composite) |  |
| Zeng (2017) | Modified Er-Xian Decoction | estrogen tablets + medroxyprogesterone acetate tablets | 24 weeks | FSH; LH; E2 |  |
| Chen (2017) | Kidney-tonifying Menstruation-regulating Ointment Prescription | estrogen tablets + medroxyprogesterone acetate tablets | 12 weeks | FSH; LH; E2 |  |
| Li LL (2018) | Kidney-tonifying and Liver-soothing Formula | estradiol valerate tablets + progesterone capsules | 12 weeks | FSH; LH; E2; AFC; Adverse events (OR; also RD sensitivity); Overall clinical effectiveness (supplementary composite) |  |
| Yu et al. (2018) | Zuo-Gui Pill | estradiol valerate tablets + progesterone capsules | 24 weeks | FSH; LH; E2 |  |
| Chen et al. (2019) | Kidney-tonifying Menstruation-regulating Formula | estradiol valerate tablets + progesterone capsules | 24 weeks | FSH; AMH; LH; E2; Adverse events (OR; also RD sensitivity); Overall clinical effectiveness (supplementary composite) |  |
| Li X (2018) | Modified Er-Xian Decoction | estradiol valerate tablets + progesterone capsules | 12 weeks | FSH; LH; E2; Adverse events (RD sensitivity only; double-zero); Overall clinical effectiveness (supplementary composite) |  |
| Lu (2019) | Kidney-tonifying Menstruation-regulating Formula | estrogen tablets + medroxyprogesterone acetate tablets | 12 weeks | FSH; LH; E2; Overall clinical effectiveness (supplementary composite) |  |
| Wang (2019) | Kidney-tonifying Ovulation-promoting Decoction | estradiol valerate tablets + medroxyprogesterone acetate | 12 weeks | FSH; LH; Adverse events (OR; also RD sensitivity); Overall clinical effectiveness (supplementary composite) |  |
| Yuan (2019) | Er-Xian Decoction | estradiol tablets + dydrogesterone tablets | 12 weeks | Overall clinical effectiveness (supplementary composite) |  |
| Zeng et al. (2019) | Kidney-tonifying and Blood-activating Formula | estradiol–cyproterone acetate tablets | 12 weeks | FSH; AMH; LH; E2; Adverse events (RD sensitivity only; double-zero) |  |
| Dong (2020) | Kidney-tonifying and Blood-activating Formula | estradiol valerate tablets + medroxyprogesterone acetate tablets | 24 weeks | FSH; AMH; LH; E2; PSV |  |
| Li (2020) | Modified Gui-Xian Decoction | estradiol tablets + dydrogesterone tablets | 12 weeks | FSH; LH; E2; Adverse events (RD sensitivity only; double-zero); Overall clinical effectiveness (supplementary composite) |  |
| Luo (2020) | Yi-Jing Decoction | estradiol tablets + dydrogesterone tablets | 12 weeks | FSH; AMH; LH; E2; AFC; Adverse events (RD sensitivity only; double-zero); Overall clinical effectiveness (supplementary composite) |  |
| Ling (2021) | Kidney-tonifying and Blood-activating Formula | estradiol valerate tablets + dydrogesterone tablets | 24 weeks | FSH; E2; Adverse events (OR; also RD sensitivity); Overall clinical effectiveness (supplementary composite) |  |
| Tang (2021) | Kidney-tonifying and Blood-activating Formula | estradiol tablets + dydrogesterone tablets | 12 weeks | FSH; AMH; LH; E2; AFC; Adverse events (RD sensitivity only; double-zero); Overall clinical effectiveness (supplementary composite) |  |
| Wang XW (2021) | Kidney-tonifying and Liver-soothing Formula | estradiol tablets + dydrogesterone tablets | 12 weeks | FSH; LH; E2; Overall clinical effectiveness (supplementary composite) |  |
| Wang HL (2021) | Kun-Tai Capsule | estradiol valerate tablets + progesterone | 12 weeks | FSH; AMH; LH; Overall clinical effectiveness (supplementary composite) |  |
| Sui (2021) | Menstruation-regulating Formula | estradiol tablets + dydrogesterone tablets | 24 weeks | AMH; PSV; Overall clinical effectiveness (supplementary composite) |  |
| Song (2021) | Zhu's Kidney-tonifying and Blood-activating Formula | estradiol tablets + dydrogesterone tablets | 12 weeks | FSH; AMH; LH; E2; PSV; Adverse events (OR; also RD sensitivity) |  |
| Wang (2022) | Modified Er-Xian Decoction | estradiol valerate tablets + dydrogesterone tablets | 12 weeks | Menstrual return/resumption; FSH; LH; E2; Adverse events (OR; also RD sensitivity); Overall clinical effectiveness (supplementary composite) |  |
| Xia (2023) | Menstruation-regulating Formula | estradiol tablets + dydrogesterone tablets | 24 weeks | FSH; AMH; LH; E2; PSV; Overall clinical effectiveness (supplementary composite) |  |
| Huang et al. (2023) | Kidney-nourishing Fertility-supporting Formula (combination) | estradiol valerate tablets + progesterone capsules | 12 weeks | FSH; AMH; LH; E2; AFC; Adverse events (OR; also RD sensitivity); Overall clinical effectiveness (supplementary composite) |  |
| Dong (2024) | Kidney-tonifying and Blood-activating Formula | estradiol tablets + dydrogesterone tablets | 12 weeks | FSH; LH; E2; AFC; Adverse events (RD sensitivity only; double-zero); Overall clinical effectiveness (supplementary composite) |  |
| Feng (2024) | Wen-Jing Decoction | estradiol valerate tablets + progesterone | 12 weeks | FSH; AMH; LH; E2; AFC; PSV; Overall clinical effectiveness (supplementary composite) |  |
| Ye (2024) | Zuo-Gui Pill combined with adjunct formula | estradiol valerate tablets + dydrogesterone | 24 weeks | FSH; AMH; LH; E2; Overall clinical effectiveness (supplementary composite) |  |
| Wang (2024) | Kidney-tonifying and Blood-activating Formula | estradiol tablets + dydrogesterone tablets | 24 weeks | Adverse events (OR; also RD sensitivity); Overall clinical effectiveness (supplementary composite) |  |
| Ren (2025) | Kidney-tonifying and Liver-soothing Formula | estradiol tablets + dydrogesterone tablets | 12 weeks | FSH; LH; E2; Adverse events (OR; also RD sensitivity); Overall clinical effectiveness (supplementary composite) |  |
| Xie (2025) | Kidney-tonifying and Menstruation-regulating Decoction | estradiol tablets + dydrogesterone tablets | 12 weeks | FSH; LH; E2; AFC; Overall clinical effectiveness (supplementary composite) |  |
| Zhou et al. (2025) | Kidney-nourishing and Meridian-soothing Decoction | estrogen tablets + medroxyprogesterone acetate tablets | 12 weeks | Adverse events (RD sensitivity only; double-zero); Overall clinical effectiveness (supplementary composite) |  |
| Xue (2026) | Kidney-tonifying and Blood-nourishing Formula | estradiol valerate tablets + progesterone | 12 weeks | Menstrual return/resumption; FSH; LH; E2; Adverse events (OR; also RD sensitivity); Overall clinical effectiveness (supplementary composite) |  |
| Wen (2015) | Kidney-tonifying Menstruation-restoring Formula | medroxyprogesterone acetate + estradiol valerate tablets | 24 weeks | FSH; LH; E2; Adverse events (OR; also RD sensitivity); Overall clinical effectiveness (supplementary composite) |  |
| Cheng (2016) | Modified Yu-Yin-Ling Formula | estrogen tablets + medroxyprogesterone acetate | 12 weeks | FSH; LH; E2; Adverse events (OR; also RD sensitivity); Overall clinical effectiveness (supplementary composite) |  |
| Jiang (2016) | Kidney-tonifying Cycle-regulating Therapy | estradiol valerate tablets + medroxyprogesterone acetate | 12 weeks | FSH; LH; E2; Overall clinical effectiveness (supplementary composite) |  |
| Zheng (2009) | Kidney-tonifying, Liver-regulating and Blood-activating Formula | diethylstilbestrol + medroxyprogesterone acetate | 12 weeks | FSH; LH; E2; Overall clinical effectiveness (supplementary composite) |  |

T, treatment group; C, control group; HRT, hormone replacement therapy; RCT, randomized controlled trial; NR, not reported. Study labels and order follow the frozen final forest-plot naming file. Outcome availability reflects the final cleaned analysis datasets and final figure set. Menstrual return/resumption was listed only for the four-study main analysis, with Xu et al. (2012) identified as sensitivity-only. Adverse-event contributions are distinguished as OR plus RD or RD-only double-zero records. Overall clinical effectiveness remained a supplementary composite outcome.

# Table S6. Therapeutic-principle classification of included formulas

| **Study** | **Formula name** | **Therapeutic description** | **Final classification (PK/KL/KB)** | **Classification basis (Level)** | **Classification certainty** | **Action in Table S16 sensitivity analysis** | **Notes** |
| --- | --- | --- | --- | --- | --- | --- | --- |
| Hua et al. (2012) | Bu-Shui-Rou-Mu Formula | kidney-tonifying; liver-soothing | KL | Level 1 | High | Retained in Level 3 sensitivity |  |
| Xu et al. (2012) | Modified Yi-Jing Decoction | based on core herbs: kidney-tonifying; blood-activating | KB | Level 3 | Low | Excluded from Level 3 sensitivity |  |
| Jin (2013) | Kidney-tonifying and Blood-activating Formula | kidney-tonifying; blood-activating | KB | Level 1 | High | Retained in Level 3 sensitivity |  |
| Xu (2013) | Kidney-tonifying Menstruation-regulating Formula | kidney-tonifying | PK | Level 1 | High | Retained in Level 3 sensitivity |  |
| Xu et al. (2014) | Kidney-tonifying Menstruation-regulating Formula | kidney-tonifying | PK | Level 1 | High | Retained in Level 3 sensitivity |  |
| Wen (2015) | Kidney-tonifying Menstruation-restoring Formula | based on core herbs: kidney-tonifying | PK | Level 3 | Low | Excluded from Level 3 sensitivity |  |
| Cheng (2016) | Modified Yu-Yin-Ling Formula | liver–kidney yin deficiency pattern | KL | Level 2 | Moderate | Retained in Level 3 sensitivity |  |
| Zeng (2017) | Modified Er-Xian Decoction | kidney-tonifying | PK | Level 1 | High | Retained in Level 3 sensitivity |  |
| Li LL (2018) | Kidney-tonifying and Liver-soothing Formula | kidney deficiency with liver qi stagnation | KL | Level 2 | Moderate | Retained in Level 3 sensitivity |  |
| Yu et al. (2018) | Zuo-Gui Pill | kidney-tonifying | PK | Level 1 | High | Retained in Level 3 sensitivity |  |
| Chen et al. (2019) | Kidney-tonifying Menstruation-regulating Formula | kidney-tonifying; blood-activating | KB | Level 1 | High | Retained in Level 3 sensitivity |  |
| Li X (2018) | Modified Er-Xian Decoction | based on core herbs: kidney-tonifying | PK | Level 3 | Low | Excluded from Level 3 sensitivity |  |
| Lu (2019) | Kidney-tonifying Menstruation-regulating Formula | based on core herbs: kidney-tonifying | PK | Level 3 | Low | Excluded from Level 3 sensitivity |  |
| Yuan (2019) | Er-Xian Decoction | based on core herbs: kidney-tonifying | PK | Level 3 | Low | Excluded from Level 3 sensitivity |  |
| Zeng et al. (2019) | Kidney-tonifying and Blood-activating Formula | kidney-tonifying; blood-activating | KB | Level 1 | High | Retained in Level 3 sensitivity |  |
| Dong (2020) | Kidney-tonifying and Blood-activating Formula | kidney-tonifying; blood-activating | KB | Level 1 | High | Retained in Level 3 sensitivity |  |
| Li (2020) | Modified Gui-Xian Decoction | kidney deficiency with liver qi stagnation pattern | KL | Level 2 | Moderate | Retained in Level 3 sensitivity |  |
| Luo (2020) | Yi-Jing Decoction | kidney deficiency with liver qi stagnation pattern | KL | Level 2 | Moderate | Retained in Level 3 sensitivity |  |
| Sui (2021) | Menstruation-regulating Formula | kidney deficiency and blood stasis pattern | KB | Level 2 | Moderate | Retained in Level 3 sensitivity |  |
| Ling (2021) | Kidney-tonifying and Blood-activating Formula | kidney-tonifying; blood-activating | KB | Level 1 | High | Retained in Level 3 sensitivity |  |
| Tang (2021) | Kidney-tonifying and Blood-activating Formula | kidney-tonifying; blood-activating | KB | Level 1 | High | Retained in Level 3 sensitivity |  |
| Wang XW (2021) | Kidney-tonifying and Liver-soothing Formula | kidney deficiency with liver qi stagnation pattern | KL | Level 2 | Moderate | Retained in Level 3 sensitivity |  |
| Wang (2022) | Modified Er-Xian Decoction | kidney deficiency with liver qi stagnation pattern | KL | Level 2 | Moderate | Retained in Level 3 sensitivity |  |
| Huang et al. (2023) | Kidney-nourishing Fertility-supporting Formula (combination) | kidney-tonifying | PK | Level 1 | High | Retained in Level 3 sensitivity |  |
| Xia (2023) | Menstruation-regulating Formula | kidney-tonifying; blood-activating | KB | Level 1 | High | Retained in Level 3 sensitivity |  |
| Dong (2024) | Kidney-tonifying and Blood-activating Formula | kidney-tonifying; blood-activating | KB | Level 1 | High | Retained in Level 3 sensitivity |  |
| Ye (2024) | Zuo-Gui Pill combined with adjunct formula | kidney-tonifying | PK | Level 1 | High | Retained in Level 3 sensitivity |  |
| Ren (2025) | Kidney-tonifying and Liver-soothing Formula | liver–kidney yin deficiency pattern | KL | Level 2 | Moderate | Retained in Level 3 sensitivity |  |
| Xie (2025) | Kidney-tonifying and Menstruation-regulating Decoction | kidney-tonifying; liver-soothing | KL | Level 1 | High | Retained in Level 3 sensitivity |  |
| Chen (2017) | Kidney-tonifying Menstruation-regulating Ointment Prescription | kidney deficiency pattern | PK | Level 2 | Moderate | Retained in Level 3 sensitivity |  |
| Feng (2024) | Wen-Jing Decoction | kidney-tonifying; blood-activating | KB | Level 1 | High | Retained in Level 3 sensitivity |  |
| Jiang (2016) | Kidney-tonifying Cycle-regulating Therapy | kidney-tonifying | PK | Level 1 | High | Retained in Level 3 sensitivity |  |
| Li (2012) | Yi-Jing Decoction | kidney-tonifying; liver-soothing | KL | Level 1 | High | Retained in Level 3 sensitivity |  |
| Song (2021) | Zhu's Kidney-tonifying and Blood-activating Formula | kidney deficiency and blood stasis pattern | KB | Level 2 | Moderate | Retained in Level 3 sensitivity |  |
| Wang HL (2021) | Kun-Tai Capsule | kidney-tonifying | PK | Level 1 | High | Retained in Level 3 sensitivity |  |
| Wang (2019) | Kidney-tonifying Ovulation-promoting Decoction | based on core herbs: kidney-tonifying | PK | Level 3 | Low | Excluded from Level 3 sensitivity |  |
| Wang (2024) | Kidney-tonifying and Blood-activating Formula | kidney deficiency and blood stasis pattern | KB | Level 2 | Moderate | Retained in Level 3 sensitivity |  |
| Xu et al. (2017) | Kidney-tonifying Menstruation-regulating Ointment | kidney deficiency pattern | PK | Level 2 | Moderate | Retained in Level 3 sensitivity |  |
| Zheng (2009) | Kidney-tonifying, Liver-regulating and Blood-activating Formula | kidney-tonifying; blood-activating | KB | Level 1 | High | Retained in Level 3 sensitivity |  |
| Zhou et al. (2025) | Kidney-nourishing and Meridian-soothing Decoction | kidney deficiency with liver qi stagnation pattern | KL | Level 2 | Moderate | Retained in Level 3 sensitivity |  |
| Xue (2026) | Kidney-tonifying and Blood-nourishing Formula | kidney-tonifying | PK | Level 1 | High | Retained in Level 3 sensitivity |  |

Footnote: PK, kidney-tonifying alone; KL, kidney-tonifying plus liver-soothing; KB, kidney-tonifying plus blood-activating. Level 1 = High certainty (explicit therapeutic function/principle stated); Level 2 = Moderate certainty (clear treated TCM pattern); Level 3 = Low certainty (classification based on core herb actions). Table S16 excluded Level 3 classifications only.

# Table S7. Chinese materia medica names and frequency in the final 41-study dataset

Dataset: revised 41 HRT-controlled RCTs. Frequency indicates the number of studies in which each materia medica appeared at least once in the oral herbal formula.

Only final 41 HRT-controlled RCTs were counted. Placebo-controlled, clomiphene-containing comparator, and potential-overlap records were not counted.

| **No.** | **Latin name** | **Chinese name** | **Chinese pinyin** | **English name** | **Frequency (studies)** | **Frequency, %** | **Study labels** |
| --- | --- | --- | --- | --- | --- | --- | --- |
| 1 | Rehmanniae Radix Praeparata | 熟地黄 | Shu Di Huang | Prepared rehmannia root | 34 | 82.9 | Hua et al. (2012); Xu et al. (2012); Jin (2013); Xu (2013); Xu et al. (2014); Wen (2015); Cheng (2016); Li LL (2018); Yu et al. (2018); Chen et al. (2019); Li X (2018); Lu (2019); Yuan (2019); Zeng et al. (2019); Dong (2020); Luo (2020); Sui (2021); Tang (2021); Wang XW (2021); Huang et al. (2023); Xia (2023); Ye (2024); Ren (2025); Xie (2025); Chen (2017); Li (2012); Song (2021); Wang HL (2021); Wang (2019); Wang (2024); Xu et al. (2017); Zheng (2009); Zhou et al. (2025); Xue (2026) |
| 2 | Angelicae Sinensis Radix | 当归 | Dang Gui | Chinese angelica root | 30 | 73.2 | Hua et al. (2012); Xu et al. (2012); Jin (2013); Xu (2013); Xu et al. (2014); Wen (2015); Zeng (2017); Li LL (2018); Chen et al. (2019); Li X (2018); Yuan (2019); Zeng et al. (2019); Dong (2020); Li (2020); Luo (2020); Sui (2021); Ling (2021); Wang XW (2021); Wang (2022); Huang et al. (2023); Xia (2023); Dong (2024); Ren (2025); Xie (2025); Feng (2024); Song (2021); Wang (2024); Xu et al. (2017); Zheng (2009); Xue (2026) |
| 3 | Cuscutae Semen | 菟丝子 | Tu Si Zi | Dodder seed | 29 | 70.7 | Jin (2013); Xu (2013); Xu et al. (2014); Wen (2015); Cheng (2016); Li LL (2018); Yu et al. (2018); Chen et al. (2019); Lu (2019); Zeng et al. (2019); Dong (2020); Li (2020); Sui (2021); Tang (2021); Wang (2022); Huang et al. (2023); Xia (2023); Dong (2024); Ye (2024); Ren (2025); Xie (2025); Chen (2017); Li (2012); Song (2021); Wang (2019); Wang (2024); Xu et al. (2017); Zhou et al. (2025); Xue (2026) |
| 4 | Dioscoreae Rhizoma | 山药 | Shan Yao | Chinese yam rhizome | 19 | 46.3 | Hua et al. (2012); Xu et al. (2012); Xu (2013); Xu et al. (2014); Wen (2015); Cheng (2016); Zeng (2017); Yu et al. (2018); Chen et al. (2019); Luo (2020); Ling (2021); Tang (2021); Wang XW (2021); Ye (2024); Xie (2025); Chen (2017); Xu et al. (2017); Zhou et al. (2025); Xue (2026) |
| 5 | Epimedii Folium | 淫羊藿 | Yin Yang Huo | Epimedium leaf | 18 | 43.9 | Hua et al. (2012); Wen (2015); Zeng (2017); Li X (2018); Yuan (2019); Zeng et al. (2019); Dong (2020); Li (2020); Sui (2021); Wang (2022); Xia (2023); Dong (2024); Xie (2025); Song (2021); Wang (2019); Wang (2024); Xu et al. (2017); Zheng (2009) |
| 6 | Corni Fructus | 山茱萸 | Shan Zhu Yu | Cornelian cherry fruit | 17 | 41.5 | Hua et al. (2012); Jin (2013); Xu (2013); Xu et al. (2014); Wen (2015); Zeng (2017); Yu et al. (2018); Chen et al. (2019); Lu (2019); Tang (2021); Wang XW (2021); Dong (2024); Ye (2024); Chen (2017); Xu et al. (2017); Zheng (2009); Xue (2026) |
| 7 | Paeoniae Radix Alba | 白芍 | Bai Shao | White peony root | 16 | 39.0 | Hua et al. (2012); Xu et al. (2012); Jin (2013); Cheng (2016); Li LL (2018); Chen et al. (2019); Zeng et al. (2019); Li (2020); Luo (2020); Tang (2021); Wang XW (2021); Huang et al. (2023); Feng (2024); Wang HL (2021); Zhou et al. (2025); Xue (2026) |
| 8 | Morindae Officinalis Radix | 巴戟天 | Ba Ji Tian | Morinda root | 14 | 34.1 | Xu et al. (2012); Wen (2015); Chen et al. (2019); Li X (2018); Yuan (2019); Dong (2020); Li (2020); Sui (2021); Xia (2023); Xie (2025); Li (2012); Song (2021); Wang (2024); Xu et al. (2017) |
| 9 | Salviae Miltiorrhizae Radix et Rhizoma | 丹参 | Dan Shen | Red sage root and rhizome | 14 | 34.1 | Xu et al. (2012); Cheng (2016); Li LL (2018); Dong (2020); Sui (2021); Ling (2021); Tang (2021); Wang (2022); Xia (2023); Ren (2025); Li (2012); Song (2021); Wang (2024); Zheng (2009) |
| 10 | Glycyrrhizae Radix et Rhizoma | 甘草 | Gan Cao | Licorice root and rhizome | 13 | 31.7 | Hua et al. (2012); Zeng (2017); Li LL (2018); Chen et al. (2019); Li X (2018); Yuan (2019); Zeng et al. (2019); Li (2020); Ling (2021); Wang XW (2021); Wang (2022); Huang et al. (2023); Feng (2024) |
| 11 | Poria | 茯苓 | Fu Ling | Poria | 10 | 24.4 | Hua et al. (2012); Xu (2013); Xu et al. (2014); Lu (2019); Ling (2021); Huang et al. (2023); Chen (2017); Wang HL (2021); Xu et al. (2017); Zhou et al. (2025) |
| 12 | Lycii Fructus | 枸杞子 | Gou Qi Zi | Goji berry | 10 | 24.4 | Xu (2013); Xu et al. (2014); Yu et al. (2018); Chen et al. (2019); Ling (2021); Ye (2024); Chen (2017); Li (2012); Xu et al. (2017); Xue (2026) |
| 13 | Moutan Cortex | 牡丹皮 | Mu Dan Pi | Tree peony root bark | 10 | 24.4 | Xu (2013); Xu et al. (2014); Li X (2018); Yuan (2019); Luo (2020); Ling (2021); Wang XW (2021); Chen (2017); Feng (2024); Xu et al. (2017) |
| 14 | Ligustri Lucidi Fructus | 女贞子 | Nu Zhen Zi | Glossy privet fruit | 10 | 24.4 | Jin (2013); Li X (2018); Yuan (2019); Zeng et al. (2019); Li (2020); Wang XW (2021); Ren (2025); Xie (2025); Xu et al. (2017); Zheng (2009) |
| 15 | Chuanxiong Rhizoma | 川芎 | Chuan Xiong | Szechuan lovage rhizome | 9 | 22.0 | Jin (2013); Wen (2015); Chen et al. (2019); Li X (2018); Yuan (2019); Ling (2021); Huang et al. (2023); Xie (2025); Feng (2024) |
| 16 | Hominis Placenta | 紫河车 | Zi He Che | Human placenta | 9 | 22.0 | Cheng (2016); Chen et al. (2019); Dong (2020); Sui (2021); Ling (2021); Xia (2023); Xie (2025); Song (2021); Zheng (2009) |
| 17 | Atractylodis Macrocephalae Rhizoma | 白术 | Bai Zhu | White atractylodes rhizome | 8 | 19.5 | Hua et al. (2012); Xu et al. (2012); Li LL (2018); Luo (2020); Ling (2021); Tang (2021); Huang et al. (2023); Xu et al. (2017) |
| 18 | Cyathulae Radix | 川牛膝 | Chuan Niu Xi | Cyathula root | 8 | 19.5 | Xu (2013); Xu et al. (2014); Lu (2019); Tang (2021); Ye (2024); Chen (2017); Xu et al. (2017); Zhou et al. (2025) |
| 19 | Codonopsis Radix | 党参 | Dang Shen | Codonopsis root | 8 | 19.5 | Xu et al. (2012); Zeng et al. (2019); Dong (2020); Sui (2021); Xia (2023); Song (2021); Wang (2019); Wang (2024) |
| 20 | Rubi Fructus | 覆盆子 | Fu Pen Zi | Raspberry fruit | 8 | 19.5 | Wen (2015); Dong (2020); Sui (2021); Xia (2023); Dong (2024); Song (2021); Wang (2024); Xu et al. (2017) |
| 21 | Curculiginis Rhizoma | 仙茅 | Xian Mao | Curculigo rhizome | 8 | 19.5 | Hua et al. (2012); Zeng (2017); Li X (2018); Yuan (2019); Li (2020); Wang (2022); Xu et al. (2017); Zheng (2009) |
| 22 | Cyperi Rhizoma | 香附 | Xiang Fu | Nutgrass galingale rhizome | 8 | 19.5 | Hua et al. (2012); Cheng (2016); Li (2020); Wang XW (2021); Xie (2025); Li (2012); Zheng (2009); Zhou et al. (2025) |
| 23 | Bupleuri Radix | 柴胡 | Chai Hu | Bupleurum root | 7 | 17.1 | Hua et al. (2012); Xu et al. (2012); Li LL (2018); Li (2020); Luo (2020); Wang (2022); Zhou et al. (2025) |
| 24 | Astragali Radix | 黄芪 | Huang Qi | Astragalus root | 7 | 17.1 | Dong (2020); Sui (2021); Xia (2023); Song (2021); Wang (2019); Wang (2024); Xu et al. (2017) |
| 25 | Dipsaci Radix | 续断 | Xu Duan | Dipsacus root | 7 | 17.1 | Xu (2013); Xu et al. (2014); Ling (2021); Chen (2017); Wang (2019); Xu et al. (2017); Zhou et al. (2025) |
| 26 | Leonuri Herba | 益母草 | Yi Mu Cao | Motherwort herb | 7 | 17.1 | Xu (2013); Xu et al. (2014); Li LL (2018); Li X (2018); Yuan (2019); Chen (2017); Xu et al. (2017) |
| 27 | Eucommiae Cortex | 杜仲 | Du Zhong | Eucommia bark | 6 | 14.6 | Xu et al. (2012); Cheng (2016); Luo (2020); Huang et al. (2023); Ren (2025); Wang (2019) |
| 28 | Asini Corii Colla | 阿胶 | E Jiao | Donkey-hide gelatin | 5 | 12.2 | Feng (2024); Wang HL (2021); Wang (2019); Xu et al. (2017); Xue (2026) |
| 29 | Cornu Cervi Degelatinum | 鹿角霜 | Lu Jiao Shuang | Degelatinated deer antler | 4 | 9.8 | Li X (2018); Yuan (2019); Huang et al. (2023); Xie (2025) |
| 30 | Anemarrhenae Rhizoma | 知母 | Zhi Mu | Anemarrhena rhizome | 4 | 9.8 | Li X (2018); Yuan (2019); Ren (2025); Wang (2019) |
| 31 | Testudinis Plastrum | 龟板 | Gui Ban | Tortoise plastron | 3 | 7.3 | Cheng (2016); Tang (2021); Ren (2025) |
| 32 | Polygonati Rhizoma | 黄精 | Huang Jing | Solomonseal rhizome | 3 | 7.3 | Wen (2015); Xie (2025); Zheng (2009) |
| 33 | Spatholobi Caulis | 鸡血藤 | Ji Xue Teng | Spatholobus stem | 3 | 7.3 | Xu et al. (2012); Zeng et al. (2019); Xu et al. (2017) |
| 34 | Ecliptae Herba | 墨旱莲 | Mo Han Lian | Eclipta herb | 3 | 7.3 | Zeng et al. (2019); Ren (2025); Xu et al. (2017) |
| 35 | Ginseng Radix et Rhizoma | 人参 | Ren Shen | Ginseng root and rhizome | 3 | 7.3 | Luo (2020); Huang et al. (2023); Feng (2024) |
| 36 | Cistanches Herba | 肉苁蓉 | Rou Cong Rong | Cistanche stem | 3 | 7.3 | Zeng et al. (2019); Dong (2024); Xie (2025) |
| 37 | Cinnamomi Cortex | 肉桂 | Rou Gui | Cinnamon bark | 3 | 7.3 | Zeng (2017); Li (2020); Xu et al. (2017) |
| 38 | Taxilli Herba | 桑寄生 | Sang Ji Sheng | Taxillus twig | 3 | 7.3 | Ling (2021); Ren (2025); Wang (2019) |
| 39 | Ziziphi Spinosae Semen | 酸枣仁 | Suan Zao Ren | Spiny jujube seed | 3 | 7.3 | Xu et al. (2012); Luo (2020); Xu et al. (2017) |
| 40 | Psoraleae Fructus | 补骨脂 | Bu Gu Zhi | Psoralea fruit | 2 | 4.9 | Jin (2013); Xu et al. (2017) |
| 41 | Paeoniae Radix Rubra | 赤芍 | Chi Shao | Red peony root | 2 | 4.9 | Ling (2021); Li (2012) |
| 42 | Leonuri Fructus | 茺蔚子 | Chong Wei Zi | Motherwort fruit | 2 | 4.9 | Dong (2024); Li (2012) |
| 43 | Albiziae Cortex | 合欢皮 | He Huan Pi | Albizia bark | 2 | 4.9 | Wang XW (2021); Xu et al. (2017) |
| 44 | Achyranthis Bidentatae Radix | 怀牛膝 | Huai Niu Xi | Achyranthes root | 2 | 4.9 | Cheng (2016); Ren (2025) |
| 45 | Phellodendri Chinensis Cortex | 黄柏 | Huang Bai | Phellodendron bark | 2 | 4.9 | Li X (2018); Yuan (2019) |
| 46 | Colla Cornus Cervi | 鹿角胶 | Lu Jiao Jiao | Deer antler gelatin | 2 | 4.9 | Jin (2013); Ye (2024) |
| 47 | Achyranthis Bidentatae Radix | 牛膝 | Niu Xi | Achyranthes root | 2 | 4.9 | Li X (2018); Yuan (2019) |
| 48 | Adenophorae Radix | 沙参 | Sha Shen | Adenophora root | 2 | 4.9 | Xu et al. (2012); Luo (2020) |
| 49 | Zingiberis Rhizoma Recens | 生姜 | Sheng Jiang | Fresh ginger | 2 | 4.9 | Li LL (2018); Feng (2024) |
| 50 | Dendrobii Caulis | 石斛 | Shi Hu | Dendrobium stem | 2 | 4.9 | Ren (2025); Xie (2025) |
| 51 | Akebiae Fructus | 八月札 | Ba Yue Zha | Akebia fruit | 1 | 2.4 | Zheng (2009) |
| 52 | Platycladi Semen | 柏子仁 | Bai Zi Ren | Chinese arborvitae kernel | 1 | 2.4 | Xu et al. (2017) |
| 53 | Pinelliae Rhizoma | 半夏 | Ban Xia | Pinellia rhizome | 1 | 2.4 | Feng (2024) |
| 54 | Glehniae Radix | 北沙参 | Bei Sha Shen | Glehnia root | 1 | 2.4 | Ren (2025) |
| 55 | Trionycis Carapax | 鳖甲 | Bie Jia | Soft-shelled turtle carapace | 1 | 2.4 | Ren (2025) |
| 56 | Citri Reticulatae Pericarpium | 陈皮 | Chen Pi | Tangerine peel | 1 | 2.4 | Zhou et al. (2025) |
| 57 | Zanthoxyli Pericarpium | 川椒 | Chuan Jiao | Sichuan pepper pericarp | 1 | 2.4 | Huang et al. (2023) |
| 58 | Citri Sarcodactylis Fructus | 佛手 | Fo Shou | Finger citron fruit | 1 | 2.4 | Wang XW (2021) |
| 59 | Tritici Levis Fructus | 浮小麦 | Fu Xiao Mai | Blighted wheat | 1 | 2.4 | Xu et al. (2017) |
| 60 | Rehmanniae Radix | 干地黄 | Gan Di Huang | Dried rehmannia root | 1 | 2.4 | Dong (2024) |
| 61 | Colla Plastri Testudinis | 龟板胶 | Gui Ban Jiao | Tortoise plastron gelatin | 1 | 2.4 | Ye (2024) |
| 62 | Cinnamomi Ramulus | 桂枝 | Gui Zhi | Cinnamon twig | 1 | 2.4 | Feng (2024) |
| 63 | Polygoni Multiflori Radix | 何首乌 | He Shou Wu | Fo-ti root | 1 | 2.4 | Zeng et al. (2019) |
| 64 | Polygoni Multiflori Caulis | 何首乌藤 | He Shou Wu Teng | Fo-ti stem | 1 | 2.4 | Xu et al. (2017) |
| 65 | Carthami Flos | 红花 | Hong Hua | Safflower | 1 | 2.4 | Jin (2013) |
| 66 | Sargentodoxae Caulis | 红藤 | Hong Teng | Sargentodoxa stem | 1 | 2.4 | Ling (2021) |
| 67 | Coptidis Rhizoma | 黄连 | Huang Lian | Coptis rhizome | 1 | 2.4 | Wang HL (2021) |
| 68 | Scutellariae Radix | 黄芩 | Huang Qin | Baikal skullcap root | 1 | 2.4 | Wang HL (2021) |
| 69 | Bombyx Batryticatus | 僵蚕 | Jiang Can | Stiff silkworm | 1 | 2.4 | Li (2012) |
| 70 | Citri Reticulatae Folium | 橘叶 | Ju Ye | Tangerine leaf | 1 | 2.4 | Hua et al. (2012) |
| 71 | Mume Flos | 绿萼梅 | Lu E Mei | Japanese apricot flower | 1 | 2.4 | Ren (2025) |
| 72 | Ophiopogonis Radix | 麦冬 | Mai Dong | Ophiopogon root | 1 | 2.4 | Feng (2024) |
| 73 | Rehmanniae Radix | 生地 | Sheng Di | Fresh rehmannia root | 1 | 2.4 | Ling (2021) |
| 74 | Persicae Semen | 桃仁 | Tao Ren | Peach kernel | 1 | 2.4 | Jin (2013) |
| 75 | Asparagi Radix | 天冬 | Tian Dong | Asparagus tuber | 1 | 2.4 | Ren (2025) |
| 76 | Tribuli Fructus | 潼蒺藜 | Tong Ji Li | Tribulus fruit | 1 | 2.4 | Li (2020) |
| 77 | Schisandrae Chinensis Fructus | 五味子 | Wu Wei Zi | Schisandra fruit | 1 | 2.4 | Xu et al. (2017) |
| 78 | Linderae Radix | 乌药 | Wu Yao | Lindera root | 1 | 2.4 | Dong (2024) |
| 79 | Euodiae Fructus | 吴茱萸 | Wu Zhu Yu | Evodia fruit | 1 | 2.4 | Feng (2024) |
| 80 | Scrophulariae Radix | 玄参 | Xuan Shen | Figwort root | 1 | 2.4 | Wang (2019) |
| 81 | Coicis Semen | 薏苡仁 | Yi Yi Ren | Coix seed | 1 | 2.4 | Ling (2021) |
| 82 | Margaritifera Concha | 珍珠母 | Zhen Zhu Mu | Mother-of-pearl | 1 | 2.4 | Wang (2019) |
| 83 | Polygalae Radix Praeparata | 远志 | Yuan Zhi | Prepared polygala root | 1 | 2.4 | Wang XW (2021) |
| 84 | Fluoritum | 紫石英 | Zi Shi Ying | Purple fluorite | 1 | 2.4 | Li (2020) |

# Table S8. Risk-of-bias assessments of included studies using the Cochrane RoB 1 tool

| **Study ID** | **Article / Study label** | **Random sequence generation** | **Allocation concealment** | **Blinding of participants and personnel** | **Blinding of outcome assessment** | **Incomplete outcome data** | **Selective outcome reporting** | **Other bias** | **Overall risk-of-bias interpretation** |
| --- | --- | --- | --- | --- | --- | --- | --- | --- | --- |
| Zeng_2019 | Zeng et al. (2019) | Low | Unclear | Unclear | Unclear | Unclear | Unclear | Unclear | Unclear overall risk |
| Zeng_2017 | Zeng (2017) | Low | Unclear | Unclear | Unclear | Unclear | Unclear | Low | Unclear overall risk |
| Chen_2019 | Chen et al. (2019) | Low | Unclear | Unclear | Unclear | Unclear | Unclear | Low | Unclear overall risk |
| Cheng_2016 | Cheng (2016) | Low | Unclear | Unclear | Unclear | Unclear | Unclear | Low | Unclear overall risk |
| Dong_2020 | Dong (2020) | Low | Unclear | Unclear | Unclear | Low | Unclear | Low | Unclear overall risk |
| Dong_2024 | Dong (2024) | Low | Unclear | Unclear | Unclear | Unclear | Unclear | Low | Unclear overall risk |
| Hua_2012 | Hua et al. (2012) | Low | Unclear | Unclear | Unclear | Unclear | Unclear | Unclear | Unclear overall risk |
| Huang_2023 | Huang et al. (2023) | Low | Unclear | Unclear | Unclear | Unclear | Unclear | Low | Unclear overall risk |
| Jin_2013 | Jin (2013) | Unclear | Unclear | Unclear | Unclear | Unclear | Unclear | Unclear | Unclear overall risk |
| Li_2020 | Li (2020) | Low | Unclear | Unclear | Unclear | Unclear | Unclear | Low | Unclear overall risk |
| Li_LL_2018 | Li LL (2018) | Low | Unclear | Unclear | Unclear | Unclear | Unclear | Low | Unclear overall risk |
| Li_X_2018 | Li X (2018) | Low | Unclear | Unclear | Unclear | Unclear | Unclear | Low | Unclear overall risk |
| Ling_2021 | Ling (2021) | Low | Unclear | Unclear | Unclear | Unclear | Unclear | Low | Unclear overall risk |
| Lu_2019 | Lu (2019) | Low | Unclear | Unclear | Unclear | Unclear | Unclear | Low | Unclear overall risk |
| Luo_2020 | Luo (2020) | Low | Unclear | Unclear | Unclear | Unclear | Unclear | Low | Unclear overall risk |
| Ren_2025 | Ren (2025) | Low | Unclear | Unclear | Unclear | Low | Unclear | Low | Unclear overall risk |
| Sui_2021 | Sui (2021) | Low | Unclear | Unclear | Unclear | Low | Unclear | Low | Unclear overall risk |
| Tang_2021 | Tang (2021) | Low | Unclear | Unclear | Unclear | Unclear | Unclear | Low | Unclear overall risk |
| Wang_XW_2021 | Wang XW (2021) | Low | Unclear | Unclear | Unclear | Unclear | Unclear | Low | Unclear overall risk |
| Wang_2022 | Wang (2022) | Low | Unclear | Unclear | Unclear | Unclear | Unclear | Low | Unclear overall risk |
| Wen_2015 | Wen (2015) | Low | Unclear | Unclear | Unclear | Unclear | Unclear | Low | Unclear overall risk |
| Xia_2023 | Xia (2023) | Low | Low | Unclear | Unclear | Low | Unclear | Low | Unclear overall risk |
| Xie_2025 | Xie (2025) | Low | Unclear | Unclear | Unclear | Unclear | Unclear | Low | Unclear overall risk |
| Xu_2013 | Xu (2013) | Low | Unclear | Unclear | Unclear | Unclear | Unclear | Low | Unclear overall risk |
| Xu_2014 | Xu et al. (2014) | Low | Unclear | Unclear | Unclear | Low | Unclear | Low | Unclear overall risk |
| Xu_2012 | Xu et al. (2012) | Unclear | Unclear | Unclear | Unclear | Unclear | Unclear | Low | Unclear overall risk |
| Ye_2024 | Ye (2024) | Low | Unclear | Unclear | Unclear | Unclear | Unclear | Low | Unclear overall risk |
| Yu_2018 | Yu et al. (2018) | Low | Unclear | Unclear | Unclear | Unclear | Unclear | Low | Unclear overall risk |
| Yuan_2019 | Yuan (2019) | Low | Unclear | Unclear | Unclear | Unclear | Unclear | Low | Unclear overall risk |
| Feng_2024 | Feng (2024) | Low | Unclear | Unclear | Unclear | Unclear | Unclear | Low | Unclear overall risk |
| Zhou_2025 | Zhou et al. (2025) | Low | Unclear | Unclear | Unclear | Unclear | Unclear | Low | Unclear overall risk |
| Song_2021 | Song (2021) | Unclear | Unclear | Unclear | Unclear | Low | Unclear | Low | Unclear overall risk |
| Xu_2017 | Xu et al. (2017) | Low | Unclear | Unclear | Unclear | Low | Unclear | Low | Unclear overall risk |
| Li_2012 | Li (2012) | Low | Unclear | Unclear | Unclear | Unclear | Unclear | Unclear | Unclear overall risk |
| Jiang_2016 | Jiang (2016) | Low | Unclear | Unclear | Unclear | Unclear | Unclear | Unclear | Unclear overall risk |
| Wang_2024 | Wang (2024) | Low | Unclear | Unclear | Unclear | Unclear | Unclear | Low | Unclear overall risk |
| Wang_HL_2021 | Wang HL (2021) | Low | Unclear | Unclear | Unclear | Unclear | Unclear | Low | Unclear overall risk |
| Wang_2019 | Wang (2019) | Low | Unclear | Unclear | Unclear | Unclear | Unclear | Low | Unclear overall risk |
| Xue_2026 | Xue (2026) | Low | Unclear | Unclear | Unclear | Unclear | Unclear | Low | Unclear overall risk |
| Zheng_2009 | Zheng (2009) | Low | Unclear | Unclear | Unclear | Unclear | Unclear | Low | Unclear overall risk |
| Chen_2017 | Chen (2017) | Low | Unclear | Unclear | Unclear | Unclear | Unclear | Low | Unclear overall risk |

RoB1 judgments were based on the Cochrane Risk of Bias 1 tool. Unclear indicates insufficient information in the trial report to support a low- or high-risk judgment.

# Table S9. Domain-level summary of risk-of-bias judgments

| **RoB1 domain** | **Low, n** | **Low, %** | **Unclear, n** | **Unclear, %** | **High, n** | **High, %** | **Main interpretation** |
| --- | --- | --- | --- | --- | --- | --- | --- |
| Random sequence generation | 38 | 92.7 | 3 | 7.3 | 0 | 0 | Random sequence generation was generally adequate in most studies. |
| Allocation concealment | 1 | 2.4 | 40 | 97.6 | 0 | 0 | Allocation concealment was mostly unclear. |
| Blinding of participants and personnel | 0 | 0 | 41 | 100 | 0 | 0 | Blinding of participants and personnel was unclear in all studies. |
| Blinding of outcome assessment | 0 | 0 | 41 | 100 | 0 | 0 | Blinding of outcome assessment was unclear in all studies. |
| Incomplete outcome data | 7 | 17.1 | 34 | 82.9 | 0 | 0 | Incomplete outcome data handling was mostly unclear. |
| Selective outcome reporting | 0 | 0 | 41 | 100 | 0 | 0 | Selective outcome reporting was unclear in all studies. |
| Other bias | 36 | 87.8 | 5 | 12.2 | 0 | 0 | Other bias was generally low. |

Percentages were calculated using 41 included HRT-controlled RCTs as the denominator. These domain-level findings supported the GRADE risk-of-bias downgrade.

# Table S10. Overall REML random-effects meta-analysis results for all quantitative outcomes

| **Outcome** | **Studies** | **Participants** | **Effect measure** | **Pooled effect** | **95% CI** | **95% prediction interval** | **P value** | **I²** | **H²** | **Tau²** | **Notes** |
| --- | --- | --- | --- | --- | --- | --- | --- | --- | --- | --- | --- |
| FSH | 37 | 2,893 | MD | −5.563 | −8.230 to −2.896 | −22.125 to 10.999 | p = 0.001 | 99.13% | 115.14 | 64.7025 | Post-treatment endpoint values; negative MD favours oral Chinese herbal formula. |
| LH | 35 | 2,753 | MD | −3.005 | −4.658 to −1.353 | −12.861 to 6.851 | p = 0.001 | 98.11% | 52.97 | 22.7572 | Post-treatment endpoint values; negative MD favours oral Chinese herbal formula. |
| E2 | 35 | 2,693 | MD | 22.483 | 6.924 to 38.042 | −73.121 to 118.087 | p = 0.005 | 99.80% | 510.02 | 2145.1394 | Post-treatment endpoint values; positive MD favours oral Chinese herbal formula. |
| AMH | 12 | 1,198 | MD | 0.167 | 0.035 to 0.299 | −0.347 to 0.681 | p = 0.013 | 98.17% | 54.50 | 0.0487 | Post-treatment endpoint values; positive MD favours oral Chinese herbal formula. |
| AFC | 8 | 688 | MD | 0.457 | 0.028 to 0.886 | −1.079 to 1.992 | p = 0.037 | 94.95% | 19.80 | 0.3458 | Exploratory surrogate outcome. |
| PSV | 5 | 477 | MD | 0.570 | 0.444 to 0.697 | 0.365 to 0.776 | p = 0.001 | 0.00% | 1.00 | 0.0000003 | Exploratory hemodynamic outcome. |
| Menstrual return or resumption | 4 | 266 | OR | 1.523 | 0.315 to 7.371 | 0.001 to 2166.521 | p = 0.601 | 86.16% | 7.23 | 2.2000 | Strict directly reported menstrual-return/resumption outcome; definitions were not broadened to composite improvement. |
| Overall clinical effectiveness | 34 | 2,807 | OR | 2.187 | 1.762 to 2.715 | 1.388 to 3.447 | p = 0.001 | 9.26% | 1.10 | 0.0377 | Supplementary variably defined composite outcome only; not menstrual improvement. |
| Adverse events OR | 14 | 1,114 | OR | 0.198 | 0.109 to 0.359 | 0.060 to 0.658 | p = 0.001 | 16.67% | 1.20 | 0.2110 | Non-double-zero studies; Xu (2013) excluded because arm-level AE counts were uncertain. A 0.5 continuity correction was applied only to studies with a zero cell. |
| Adverse events RD | 22 | 1,755 | RD | −0.077 | −0.128 to −0.025 | −0.321 to 0.167 | p = 0.003 | 77.45% | 4.43 | 0.0130 | Sensitivity analysis including eight double-zero studies; Xu (2013) excluded as uncertain. |

Note: MD units were IU/L for FSH and LH, pmol/L for E2, ng/mL for AMH, follicle count for AFC, and cm/s for PSV. Tau² for OR outcomes is on the log-OR model scale. Prediction intervals were calculated separately and were not drawn in the forest plots. All models used REML.

# Table S11. Exploratory therapeutic-principle subgroup analyses and univariable meta-regression

**Part A. Therapeutic-principle subgroup analyses and subgroup-difference tests**

| **Outcome** | **Dataset** | **Group code** | **Therapeutic-principle group** | **Studies** | **Participants** | **Pooled MD (95% CI)** | **I²** | **Subgroup-difference p** | **Interpretation** |
| --- | --- | --- | --- | --- | --- | --- | --- | --- | --- |
| FSH | Main | PK | Kidney-tonifying alone | 15 | 1,183 | -5.95 (-9.13 to -2.76) | 98.81% | <0.001 | Exploratory subgroup-difference signal; substantial residual heterogeneity remained. |
| FSH | Main | KB | Kidney-tonifying plus blood-activating | 12 | 1,062 | -0.58 (-1.97 to 0.82) | 76.61% | <0.001 | Exploratory subgroup-difference signal; substantial residual heterogeneity remained. |
| FSH | Main | KL | Kidney-tonifying plus liver-soothing | 10 | 648 | -9.62 (-16.88 to -2.37) | 98.92% | <0.001 | Exploratory subgroup-difference signal; substantial residual heterogeneity remained. |
| LH | Main | PK | Kidney-tonifying alone | 15 | 1,183 | -4.13 (-6.71 to -1.55) | 98.65% | 0.065 | No statistically clear subgroup difference; exploratory only. |
| LH | Main | KB | Kidney-tonifying plus blood-activating | 11 | 982 | -0.62 (-2.38 to 1.14) | 91.37% | 0.065 | No statistically clear subgroup difference; exploratory only. |
| LH | Main | KL | Kidney-tonifying plus liver-soothing | 9 | 588 | -3.37 (-7.28 to 0.54) | 97.49% | 0.065 | No statistically clear subgroup difference; exploratory only. |
| E2 | Unit-confirmed sensitivity | PK | Kidney-tonifying alone | 10 | 770 | 23.52 (5.55 to 41.48) | 99.57% | 0.361 | No statistically clear subgroup difference; exploratory only. |
| E2 | Unit-confirmed sensitivity | KB | Kidney-tonifying plus blood-activating | 12 | 1,062 | 5.34 (-14.26 to 24.95) | 99.19% | 0.361 | No statistically clear subgroup difference; exploratory only. |
| E2 | Unit-confirmed sensitivity | KL | Kidney-tonifying plus liver-soothing | 9 | 588 | 21.41 (0.70 to 42.11) | 99.27% | 0.361 | No statistically clear subgroup difference; exploratory only. |

**Part B. Univariable REML meta-regression summary**

| **Outcome** | **Dataset** | **Moderator** | **Studies** | **Result** | **R²** | **Residual I²** | **Interpretation** |
| --- | --- | --- | --- | --- | --- | --- | --- |
| FSH | Main | Therapeutic principle | 37 | Omnibus p = 0.039; KB versus PK β = 4.831 (95% CI -1.154 to 10.817; p = 0.114); KL versus PK β = -3.660 (95% CI -9.813 to 2.493; p = 0.244) | 11.79% | 98.78% | Exploratory omnibus association signal; individual contrasts were not statistically significant and residual heterogeneity remained extreme. |
| FSH | Main | Treatment duration | 37 | β = 0.765 (95% CI -5.179 to 6.709); p = 0.801 | 0.00% | 99.01% | No statistically clear study-level moderator association; exploratory only. |
| FSH | Main; age available | Mean age | 35 | β = 0.778 (95% CI -0.497 to 2.054); p = 0.231 | 0.86% | 99.00% | No statistically clear study-level moderator association; exploratory only. |
| FSH | Main | Total sample size | 37 | β = 0.558 (95% CI -0.244 to 1.360); p = 0.173 | 2.53% | 98.96% | No statistically clear study-level moderator association; exploratory only. |
| LH | Main | Therapeutic principle | 35 | Omnibus p = 0.275; KB versus PK β = 3.139 (95% CI -0.753 to 7.032; p = 0.114); KL versus PK β = 0.702 (95% CI -3.317 to 4.721; p = 0.732) | 2.34% | 97.90% | No statistically clear study-level moderator association; exploratory only. |
| LH | Main | Treatment duration | 35 | β = -0.714 (95% CI -4.511 to 3.084); p = 0.713 | 0.00% | 98.05% | No statistically clear study-level moderator association; exploratory only. |
| LH | Main; age available | Mean age | 33 | β = 0.159 (95% CI -0.659 to 0.978); p = 0.703 | 0.00% | 97.94% | No statistically clear study-level moderator association; exploratory only. |
| LH | Main | Total sample size | 35 | β = 0.136 (95% CI -0.358 to 0.630); p = 0.589 | 0.00% | 97.95% | No statistically clear study-level moderator association; exploratory only. |
| E2 | Unit-confirmed sensitivity | Therapeutic principle | 31 | Omnibus p = 0.341; KB versus PK β = -18.296 (95% CI -45.076 to 8.484; p = 0.181); KL versus PK β = -2.181 (95% CI -30.689 to 26.327; p = 0.881) | 0.38% | 99.41% | No statistically clear study-level moderator association; exploratory only. |
| E2 | Unit-confirmed sensitivity | Treatment duration | 31 | β = -7.560 (95% CI -33.978 to 18.857); p = 0.575 | 0.00% | 99.40% | No statistically clear study-level moderator association; exploratory only. |
| E2 | Unit-confirmed; age available | Mean age | 29 | β = -1.113 (95% CI -5.828 to 3.602); p = 0.643 | 0.00% | 99.25% | No statistically clear study-level moderator association; exploratory only. |
| E2 | Unit-confirmed sensitivity | Total sample size | 31 | β = -1.709 (95% CI -4.937 to 1.518); p = 0.299 | 0.24% | 99.34% | No statistically clear study-level moderator association; exploratory only. |
| AMH | Unit-confirmed sensitivity | Treatment duration | 11 | β = -0.141 (95% CI -0.660 to 0.378); p = 0.594 | 0.00% | 99.19% | Supplementary exploratory only; limited study count and extreme residual heterogeneity. |
| AMH | Unit-confirmed; age available | Mean age | 9 | β = -0.014 (95% CI -0.215 to 0.186); p = 0.888 | 0.00% | 99.43% | Supplementary exploratory only; limited study count and extreme residual heterogeneity. |
| AMH | Unit-confirmed sensitivity | Total sample size | 11 | REML did not converge after centering; no coefficient was interpreted. | Not estimable | Not estimable | Supplementary exploratory model; reported transparently as nonconvergent. |
| FSH | Main; Levels 1-2 only | Therapeutic principle | 32 | Omnibus p = 0.035; KB versus PK β = 3.119 (95% CI -3.119 to 9.357; p = 0.327); KL versus PK β = -5.261 (95% CI -11.550 to 1.029; p = 0.101) | 14.13% | 98.43% | Exploratory omnibus association signal; individual contrasts were not statistically significant and residual heterogeneity remained extreme. |
| LH | Main; Levels 1-2 only | Therapeutic principle | 30 | Omnibus p = 0.374; KB versus PK β = 2.109 (95% CI -1.720 to 5.938; p = 0.280); KL versus PK β = -0.627 (95% CI -4.519 to 3.265; p = 0.752) | 1.09% | 97.56% | No statistically clear study-level moderator association; exploratory only. |
| E2 | Unit-confirmed; Levels 1-2 | Therapeutic principle | 28 | Omnibus p = 0.498; KB versus PK β = -10.770 (95% CI -38.124 to 16.583; p = 0.440); KL versus PK β = 4.822 (95% CI -23.645 to 33.288; p = 0.740) | 0.00% | 99.39% | No statistically clear study-level moderator association; exploratory only. |

Footnote: All subgroup and meta-regression analyses were exploratory and hypothesis-generating. PK, kidney-tonifying alone; KB, kidney-tonifying plus blood-activating; KL, kidney-tonifying plus liver-soothing. Meta-regression used study-level covariates and cannot support causal or patient-level inference. No multivariable meta-regression was performed.

# Table S12. AMH and E2 unit-conversion audit

| **Study** | **Outcome** | **Original unit** | **Original value source** | **Standardized unit** | **Conversion rule** | **Included in main analysis** | **Flagged for unit-sensitivity exclusion** | **Reason for flag** | **Notes** |
| --- | --- | --- | --- | --- | --- | --- | --- | --- | --- |
| Chen et al. (2019) | AMH | ng/mL | Verified standardized extraction record | ng/mL | No separate unit-conversion concern identified; standardized value retained. | Yes | No |  | Verified in the final standardized arm-level dataset. |
| Dong (2020) | AMH | ng/mL | Verified standardized extraction record | ng/mL | No separate unit-conversion concern identified; standardized value retained. | Yes | No |  | Verified in the final standardized arm-level dataset. |
| Feng (2024) | AMH | ng/mL | Verified standardized extraction record | ng/mL | No separate unit-conversion concern identified; standardized value retained. | Yes | No |  | Verified in the final standardized arm-level dataset. |
| Sui (2021) | AMH | ng/mL | Verified standardized extraction record | ng/mL | No separate unit-conversion concern identified; standardized value retained. | Yes | No |  | Verified in the final standardized arm-level dataset. |
| Huang et al. (2023) | AMH | ng/mL | Verified standardized extraction record | ng/mL | No separate unit-conversion concern identified; standardized value retained. | Yes | No |  | Verified in the final standardized arm-level dataset. |
| Luo (2020) | AMH | μg/L | Original trial report and verified unit-conversion audit | ng/mL | μg/L = ng/mL; numeric value unchanged | Yes | No |  | Not excluded: μg/L is equivalent to ng/mL. |
| Song (2021) | AMH | mg/L | Original trial report and verified unit-conversion audit | ng/mL | suspected/unverifiable unit assumption | Yes | Yes | original AMH unit was reported as mg/L, which is clinically implausible if interpreted literally and could not be verified. | Retained in main endpoint analysis; excluded only from AMH unit-sensitivity analysis. |
| Tang (2021) | AMH | ng/mL | Verified standardized extraction record | ng/mL | No separate unit-conversion concern identified; standardized value retained. | Yes | No |  | Verified in the final standardized arm-level dataset. |
| Wang HL (2021) | AMH | pmol/L | Original trial report and verified unit-conversion audit | ng/mL | pmol/L ÷ 7.14 = ng/mL | Yes | No |  | Not excluded: pmol/L to ng/mL is a standard AMH conversion. |
| Xia (2023) | AMH | ng/mL | Verified standardized extraction record | ng/mL | No separate unit-conversion concern identified; standardized value retained. | Yes | No |  | Verified in the final standardized arm-level dataset. |
| Ye (2024) | AMH | ng/mL | Verified standardized extraction record | ng/mL | No separate unit-conversion concern identified; standardized value retained. | Yes | No |  | Verified in the final standardized arm-level dataset. |
| Zeng et al. (2019) | AMH | ng/mL | Verified standardized extraction record | ng/mL | No separate unit-conversion concern identified; standardized value retained. | Yes | No |  | Verified in the final standardized arm-level dataset. |
| Chen (2017) | E2 | pmol/L | Verified standardized extraction record | pmol/L | No separate unit-conversion concern identified; standardized value retained. | Yes | No |  | Verified in the final standardized arm-level dataset. |
| Chen et al. (2019) | E2 | pmol/L | Verified standardized extraction record | pmol/L | No separate unit-conversion concern identified; standardized value retained. | Yes | No |  | Verified in the final standardized arm-level dataset. |
| Cheng (2016) | E2 | pmol/L | Verified standardized extraction record | pmol/L | No separate unit-conversion concern identified; standardized value retained. | Yes | No |  | Verified in the final standardized arm-level dataset. |
| Dong (2020) | E2 | pmol/L | Verified standardized extraction record | pmol/L | No separate unit-conversion concern identified; standardized value retained. | Yes | No |  | Verified in the final standardized arm-level dataset. |
| Dong (2024) | E2 | pmol/L | Verified standardized extraction record | pmol/L | No separate unit-conversion concern identified; standardized value retained. | Yes | No |  | Verified in the final standardized arm-level dataset. |
| Feng (2024) | E2 | pmol/L | Verified standardized extraction record | pmol/L | No separate unit-conversion concern identified; standardized value retained. | Yes | No |  | Verified in the final standardized arm-level dataset. |
| Hua et al. (2012) | E2 | pmol/L | Verified standardized extraction record | pmol/L | No separate unit-conversion concern identified; standardized value retained. | Yes | No |  | Verified in the final standardized arm-level dataset. |
| Huang et al. (2023) | E2 | pmol/L | Verified standardized extraction record | pmol/L | No separate unit-conversion concern identified; standardized value retained. | Yes | No |  | Verified in the final standardized arm-level dataset. |
| Jiang (2016) | E2 | pmol/L | Verified standardized extraction record | pmol/L | No separate unit-conversion concern identified; standardized value retained. | Yes | No |  | Verified in the final standardized arm-level dataset. |
| Jin (2013) | E2 | pmol/L | Verified standardized extraction record | pmol/L | No separate unit-conversion concern identified; standardized value retained. | Yes | No |  | Verified in the final standardized arm-level dataset. |
| Li (2012) | E2 | pq/mL | Original trial report and verified unit-conversion audit | pmol/L | suspected/unverifiable unit assumption | Yes | Yes | original E2 unit was reported as pq/mL, likely typographical or unverifiable. | Retained in main endpoint analysis; excluded only from E2 unit-sensitivity analysis. |
| Li LL (2018) | E2 | pmol/L | Verified standardized extraction record | pmol/L | No separate unit-conversion concern identified; standardized value retained. | Yes | No |  | Verified in the final standardized arm-level dataset. |
| Li X (2018) | E2 | pmol/L | Verified standardized extraction record | pmol/L | No separate unit-conversion concern identified; standardized value retained. | Yes | No |  | Verified in the final standardized arm-level dataset. |
| Li (2020) | E2 | pmol/L | Verified standardized extraction record | pmol/L | No separate unit-conversion concern identified; standardized value retained. | Yes | No |  | Verified in the final standardized arm-level dataset. |
| Ling (2021) | E2 | pmol/L | Verified standardized extraction record | pmol/L | No separate unit-conversion concern identified; standardized value retained. | Yes | No |  | Verified in the final standardized arm-level dataset. |
| Lu (2019) | E2 | pmol/L | Verified standardized extraction record | pmol/L | No separate unit-conversion concern identified; standardized value retained. | Yes | No |  | Verified in the final standardized arm-level dataset. |
| Luo (2020) | E2 | pmol/L | Verified standardized extraction record | pmol/L | No separate unit-conversion concern identified; standardized value retained. | Yes | No |  | Verified in the final standardized arm-level dataset. |
| Ren (2025) | E2 | pmol/L | Verified standardized extraction record | pmol/L | No separate unit-conversion concern identified; standardized value retained. | Yes | No |  | Verified in the final standardized arm-level dataset. |
| Song (2021) | E2 | ng/L | Original trial report and verified unit-conversion audit | pmol/L | ng/L treated as pg/mL; converted using × 3.671 | Yes | No |  | Not excluded: Song_2021 E2 unit is ng/L and was standard-convertible. |
| Tang (2021) | E2 | pmol/L | Verified standardized extraction record | pmol/L | No separate unit-conversion concern identified; standardized value retained. | Yes | No |  | Verified in the final standardized arm-level dataset. |
| Wang XW (2021) | E2 | pmol/L | Verified standardized extraction record | pmol/L | No separate unit-conversion concern identified; standardized value retained. | Yes | No |  | Verified in the final standardized arm-level dataset. |
| Wang (2022) | E2 | pmol/L | Verified standardized extraction record | pmol/L | No separate unit-conversion concern identified; standardized value retained. | Yes | No |  | Verified in the final standardized arm-level dataset. |
| Wen (2015) | E2 | ng/mL | Original trial report and verified unit-conversion audit | pmol/L | suspected/unverifiable unit assumption | Yes | Yes | original E2 unit was reported as ng/mL, clinically implausible for serum E2 if interpreted literally and suspected to be a unit-reporting error. | Retained in main endpoint analysis; excluded only from E2 unit-sensitivity analysis. |
| Xia (2023) | E2 | pmol/L | Verified standardized extraction record | pmol/L | No separate unit-conversion concern identified; standardized value retained. | Yes | No |  | Verified in the final standardized arm-level dataset. |
| Xie (2025) | E2 | pmol/L | Verified standardized extraction record | pmol/L | No separate unit-conversion concern identified; standardized value retained. | Yes | No |  | Verified in the final standardized arm-level dataset. |
| Xu (2013) | E2 | pmol/L | Verified standardized extraction record | pmol/L | No separate unit-conversion concern identified; standardized value retained. | Yes | No |  | Verified in the final standardized arm-level dataset. |
| Xu et al. (2012) | E2 | pmol/L | Verified standardized extraction record | pmol/L | No separate unit-conversion concern identified; standardized value retained. | Yes | No |  | Verified in the final standardized arm-level dataset. |
| Xu et al. (2014) | E2 | pmol/L | Verified standardized extraction record | pmol/L | No separate unit-conversion concern identified; standardized value retained. | Yes | No |  | Verified in the final standardized arm-level dataset. |
| Xu et al. (2017) | E2 | pmol/L | Verified standardized extraction record | pmol/L | No separate unit-conversion concern identified; standardized value retained. | Yes | No |  | Verified in the final standardized arm-level dataset. |
| Xue (2026) | E2 | pmol/L | Verified standardized extraction record | pmol/L | No separate unit-conversion concern identified; standardized value retained. | Yes | No |  | Verified in the final standardized arm-level dataset. |
| Ye (2024) | E2 | pmol/L | Verified standardized extraction record | pmol/L | No separate unit-conversion concern identified; standardized value retained. | Yes | No |  | Verified in the final standardized arm-level dataset. |
| Yu et al. (2018) | E2 | IU/L | Original trial report and verified unit-conversion audit | pmol/L | suspected/unverifiable unit assumption | Yes | Yes | original E2 unit was reported as IU/L, which is not a conventional serum E2 unit and could not be verified. | Retained in main endpoint analysis; excluded only from E2 unit-sensitivity analysis. |
| Zeng (2017) | E2 | μmol/L | Original trial report and verified unit-conversion audit | pmol/L | suspected/unverifiable unit assumption | Yes | Yes | original E2 unit was reported as μmol/L, clinically implausible if interpreted literally and unverifiable. | Retained in main endpoint analysis; excluded only from E2 unit-sensitivity analysis. |
| Zeng et al. (2019) | E2 | pmol/L | Verified standardized extraction record | pmol/L | No separate unit-conversion concern identified; standardized value retained. | Yes | No |  | Verified in the final standardized arm-level dataset. |
| Zheng (2009) | E2 | pmol/L | Verified standardized extraction record | pmol/L | No separate unit-conversion concern identified; standardized value retained. | Yes | No |  | Verified in the final standardized arm-level dataset. |

Note: This expanded table lists all AMH/E2 main-analysis study-outcome records from the cleaned dataset. For records without a specific unit-conversion flag, no separate unit-conversion concern was identified; full arm-level standardized values are provided in the cleaned dataset.

# Table S13. Unit-confirmed sensitivity analyses for E2 and AMH

| **Outcome** | **Analysis** | **Excluded studies** | **Studies** | **Participants** | **Pooled MD** | **95% CI** | **95% prediction interval** | **P value** | **I²** | **Interpretation** |
| --- | --- | --- | --- | --- | --- | --- | --- | --- | --- | --- |
| AMH | Main endpoint analysis | None | 12 | 1,198 | 0.167 | 0.035 to 0.299 | −0.347 to 0.681 | p = 0.013 | 98.17% | Main endpoint analysis retained all extractable endpoint data. |
| AMH | Unit-confirmed sensitivity | Song (2021) | 11 | 1,125 | 0.204 | 0.031 to 0.377 | −0.459 to 0.866 | p = 0.021 | 98.65% | Direction remained positive; heterogeneity and prediction-interval uncertainty remained substantial. |
| E2 | Main endpoint analysis | None | 35 | 2,693 | 22.483 | 6.924 to 38.042 | −73.121 to 118.087 | p = 0.005 | 99.80% | Main endpoint analysis retained all extractable endpoint data. |
| E2 | Unit-confirmed sensitivity | Li (2012); Wen (2015); Yu et al. (2018); Zeng (2017) | 31 | 2,420 | 16.089 | 4.826 to 27.352 | −48.831 to 81.010 | p = 0.005 | 99.61% | Direction remained positive; heterogeneity and prediction-interval uncertainty remained substantial. |

# Table S14. Structured adverse-event reporting summary

| **Study (final forest-plot label)** | **AE reporting status** | **Treatment events** | **Treatment total** | **Control events** | **Control total** | **Quantifiable for OR** | **Quantifiable for RD** | **Double-zero study** | **Uncertain excluded** | **Source-based reason or notes** |
| --- | --- | --- | --- | --- | --- | --- | --- | --- | --- | --- |
| Hua et al. (2012) | No analyzable adverse-event result reported | NR | NR | NR | NR | No | No | No | No | No analyzable adverse-event or safety result was reported; unreported events were not recoded as zero. |
| Xu et al. (2012) | Qualitative safety information only | NR | NR | NR | NR | No | No | No | No | Safety monitoring or a qualitative safety statement was reported, but no analyzable arm-level event counts were available; excluded from both OR and RD analyses. |
| Li (2012) | No analyzable adverse-event result reported | NR | NR | NR | NR | No | No | No | No | No analyzable adverse-event or safety result was reported; unreported events were not recoded as zero. |
| Jin (2013) | Explicit double-zero adverse-event data | 0 | 86 | 0 | 88 | No | Yes | Yes | No | Source p.3; 结果5; 3组均未见明显不良反应，肝肾功能正常。; 合格I/II组双零。 |
| Xu (2013) | Reported but incomplete arm-level adverse-event data | NR | 24 | 5 | 24 | No | No | No | Yes | Source p.3; only the HRT-arm total (5/24) was explicit; the herbal-arm total number with adverse events was not reported. The unreported value was not recoded as zero. |
| Xu et al. (2014) | Reported adverse-event data with arm-level event counts | 1 | 32 | 5 | 32 | Yes | Yes | No | No | Source p.4; 结果2.5; 中药组1例腹胀不适；西药组2例恶心呕吐、3例头痛。最终作者锁定分析采用32/32分母，事件数保持原文报告的1例和5例。 |
| Xu et al. (2017) | Reported adverse-event data with arm-level event counts | 1 | 23 | 3 | 23 | Yes | Yes | No | No | Source p.4; 结果2.7; 膏方组1例便秘、口干；西药组总3。; 完成者分母23/23。 |
| Zeng (2017) | No analyzable adverse-event result reported | NR | NR | NR | NR | No | No | No | No | No analyzable adverse-event or safety result was reported; unreported events were not recoded as zero. |
| Chen (2017) | No analyzable adverse-event result reported | NR | NR | NR | NR | No | No | No | No | No analyzable adverse-event or safety result was reported; unreported events were not recoded as zero. |
| Li LL (2018) | Reported adverse-event data with arm-level event counts | 0 | 39 | 2 | 39 | Yes | Yes | No | No | Source p.4; 结果2.5; 合格中药组0/39，HRT组恶心呕吐2/39。; 排除联合治疗臂。 |
| Yu et al. (2018) | No analyzable adverse-event result reported | NR | NR | NR | NR | No | No | No | No | No analyzable adverse-event or safety result was reported; unreported events were not recoded as zero. |
| Chen et al. (2019) | Reported adverse-event data with arm-level event counts | 0 | 60 | 2 | 60 | Yes | Yes | No | No | Source p.2; 结果2.4; 西药组1例胃部不适、1例乳房胀痛；中药组无事件。; 以详细结果为准。 |
| Li X (2018) | Explicit double-zero adverse-event data | 0 | 40 | 0 | 40 | No | Yes | Yes | No | Source p.4; 结果2.5; 两组肝肾功能无异常，无明显不良反应。; 明确双零。 |
| Lu (2019) | No analyzable adverse-event result reported | NR | NR | NR | NR | No | No | No | No | No analyzable adverse-event or safety result was reported; unreported events were not recoded as zero. |
| Wang (2019) | Reported adverse-event data with arm-level event counts | 2 | 50 | 11 | 50 | Yes | Yes | No | No | Source p.2; 不良反应段; 观察组2例，对照组11例出现不良反应。; 明确总受影响人数。 |
| Yuan (2019) | No analyzable adverse-event result reported | NR | NR | NR | NR | No | No | No | No | No analyzable adverse-event or safety result was reported; unreported events were not recoded as zero. |
| Zeng et al. (2019) | Explicit double-zero adverse-event data | 0 | 32 | 0 | 31 | No | Yes | Yes | No | Source p.4; 结果4; 两组患者治疗期间均未发生不良反应，未出现肝、肾功能异常。; V2漏提；新增后RD为22项。 |
| Dong (2020) | No analyzable adverse-event result reported | NR | NR | NR | NR | No | No | No | No | No analyzable adverse-event or safety result was reported; unreported events were not recoded as zero. |
| Li (2020) | Explicit double-zero adverse-event data | 0 | 28 | 0 | 26 | No | Yes | Yes | No | Source p.3; 结果4.5; 治疗过程中未发现明显不良反应。; 完成者分母28/26。 |
| Luo (2020) | Explicit double-zero adverse-event data | 0 | 30 | 0 | 30 | No | Yes | Yes | No | Source p.4; 结果2.4; 2组治疗过程中均未出现明显不良反应。; 明确双零。 |
| Ling (2021) | Reported adverse-event data with arm-level event counts | 3 | 40 | 10 | 40 | Yes | Yes | No | No | Source p.3; 表3; 中药组呕吐1、恶心2，总3；对照组总10。; 明确总事件人数。 |
| Tang (2021) | Explicit double-zero adverse-event data | 0 | 40 | 0 | 40 | No | Yes | Yes | No | Source p.4; 结果2.8; 三组未见严重不良反应，安全检查未见异常。; 合格中药/HRT臂双零。 |
| Wang XW (2021) | No analyzable adverse-event result reported | NR | NR | NR | NR | No | No | No | No | No analyzable adverse-event or safety result was reported; unreported events were not recoded as zero. |
| Wang HL (2021) | No analyzable adverse-event result reported | NR | NR | NR | NR | No | No | No | No | No analyzable adverse-event or safety result was reported; unreported events were not recoded as zero. |
| Sui (2021) | No analyzable adverse-event result reported | NR | NR | NR | NR | No | No | No | No | No analyzable adverse-event or safety result was reported; unreported events were not recoded as zero. |
| Song (2021) | Reported adverse-event data with arm-level event counts | 0 | 35 | 3 | 38 | Yes | Yes | No | No | Source p.4; 结果2.6; 西药组3例乳房胀痛、头晕等；中药组无事件。; 完成者分母35/38。 |
| Wang (2022) | Reported adverse-event data with arm-level event counts | 0 | 43 | 5 | 43 | Yes | Yes | No | No | Source p.3; 表4; 观察组0；参照组头晕3、恶心1、呕吐1，总5。; 单零研究。 |
| Xia (2023) | No analyzable adverse-event result reported | NR | NR | NR | NR | No | No | No | No | No analyzable adverse-event or safety result was reported; unreported events were not recoded as zero. |
| Huang et al. (2023) | Reported adverse-event data with arm-level event counts | 3 | 46 | 4 | 46 | Yes | Yes | No | No | Source p.3; 表7; 中药组总3；对照组总4。; 明确臂水平总数。 |
| Dong (2024) | Explicit double-zero adverse-event data | 0 | 30 | 0 | 30 | No | Yes | Yes | No | Source p.5; 结果2.6; 三组患者服药期间均未发生明显不良反应。; 明确双零。 |
| Feng (2024) | No analyzable adverse-event result reported | NR | NR | NR | NR | No | No | No | No | No analyzable adverse-event or safety result was reported; unreported events were not recoded as zero. |
| Ye (2024) | No analyzable adverse-event result reported | NR | NR | NR | NR | No | No | No | No | No analyzable adverse-event or safety result was reported; unreported events were not recoded as zero. |
| Wang (2024) | Reported adverse-event data with arm-level event counts | 2 | 61 | 1 | 62 | Yes | Yes | No | No | Source p.4; 结果2.7; 中药组大便溏薄1、腹胀1；西药组恶心1。; 明确总数。 |
| Ren (2025) | Reported adverse-event data with arm-level event counts | 2 | 45 | 8 | 45 | Yes | Yes | No | No | Source p.4; 结果2.3; 中药组便秘1、恶心1；对照组恶心2、呕吐2、头痛1、头晕3。; 明确总数2/8。 |
| Xie (2025) | No analyzable adverse-event result reported | NR | NR | NR | NR | No | No | No | No | No analyzable adverse-event or safety result was reported; unreported events were not recoded as zero. |
| Zhou et al. (2025) | Explicit double-zero adverse-event data | 0 | 35 | 0 | 35 | No | Yes | Yes | No | Source p.3; 结果2.2; 两组安全检查正常，均未出现相关不良反应。; 明确双零。 |
| Xue (2026) | Reported adverse-event data with arm-level event counts | 2 | 30 | 7 | 30 | Yes | Yes | No | No | Source p.3; 结果; 实验组轻度恶心2；对照组总体不良反应率23.3%=7/30。; 分项合计存在重叠，使用明确总人数7。 |
| Wen (2015) | Reported adverse-event data with arm-level event counts | 2 | 25 | 20 | 25 | Yes | Yes | No | No | Source p.2; 表4; 总不良反应发生率8.0%=2/25与80.0%=20/25。; 按明确总发生人数。 |
| Cheng (2016) | Reported adverse-event data with arm-level event counts | 0 | 26 | 7 | 26 | Yes | Yes | No | No | Source p.3; 结果2.5; 观察组0；对照组恶心/腹胀4、乳房胀痛3，总7。; 单零研究。 |
| Jiang (2016) | Qualitative safety information only | NR | NR | NR | NR | No | No | No | No | Safety monitoring or a qualitative safety statement was reported, but no analyzable arm-level event counts were available; excluded from both OR and RD analyses. |
| Zheng (2009) | No analyzable adverse-event result reported | NR | NR | NR | NR | No | No | No | No | No analyzable adverse-event or safety result was reported; unreported events were not recoded as zero. |

AE, adverse event; OR, odds ratio; RD, risk difference; NR, not reported. Across 41 included RCTs, 22 had quantifiable arm-level AE data: 14 non-double-zero studies entered the OR analysis and all 22 entered the RD sensitivity analysis, including 8 explicit double-zero studies. Xu (2013) was excluded because the herbal-arm total number with adverse events could not be confirmed. Two studies provided qualitative safety information only, and 16 did not report analyzable AE/safety results. Unreported events were not recoded as zero.

# Table S15. Supplementary adverse-event risk-difference analysis including double-zero studies

| **Analysis** | **Included studies** | **Participants** | **Double-zero studies included** | **Uncertain study handling** | **Effect measure** | **Pooled effect** | **95% CI** | **95% prediction interval** | **P value** | **I²** | **Model and interpretation** |
| --- | --- | --- | --- | --- | --- | --- | --- | --- | --- | --- | --- |
| RD sensitivity analysis including double-zero studies | 22 | 1,755 | 8 | Xu (2013) excluded because the herbal-arm total AE count was not explicitly reported. | Risk difference | −0.077 | −0.128 to −0.025 | −0.321 to 0.167 | p = 0.003 | 77.45% | REML random-effects. Negative RD favoured fewer reported AEs with herbal formulas, but the prediction interval crossed 0 and AE reporting remained incomplete. |

Negative RD values indicate fewer reported adverse events in the oral Chinese herbal formula group. The analysis was supplementary because event ascertainment and reporting varied across studies.

# Table S16. Classification-certainty sensitivity analyses

**Part A. Level 3 therapeutic-principle classifications excluded in the main certainty sensitivity analysis**

| **Study** | **Formula name** | **Final classification** | **Therapeutic description** | **Classification basis** | **Classification certainty** | **Sensitivity action** | **Reason** |
| --- | --- | --- | --- | --- | --- | --- | --- |
| Xu et al. (2012) | Modified Yi-Jing Decoction | KB | based on core herbs: kidney-tonifying; blood-activating | Level 3 | Low | Excluded from sensitivity analysis | Classification was based on predominant actions of core herbs rather than explicit therapeutic principle or treated TCM pattern. |
| Wen (2015) | Kidney-tonifying Menstruation-restoring Formula | PK | based on core herbs: kidney-tonifying | Level 3 | Low | Excluded from sensitivity analysis | Classification was based on predominant actions of core herbs rather than explicit therapeutic principle or treated TCM pattern. |
| Li X (2018) | Modified Er-Xian Decoction | PK | based on core herbs: kidney-tonifying | Level 3 | Low | Excluded from sensitivity analysis | Classification was based on predominant actions of core herbs rather than explicit therapeutic principle or treated TCM pattern. |
| Lu (2019) | Kidney-tonifying Menstruation-regulating Formula | PK | based on core herbs: kidney-tonifying | Level 3 | Low | Excluded from sensitivity analysis | Classification was based on predominant actions of core herbs rather than explicit therapeutic principle or treated TCM pattern. |
| Yuan (2019) | Er-Xian Decoction | PK | based on core herbs: kidney-tonifying | Level 3 | Low | Excluded from sensitivity analysis | Classification was based on predominant actions of core herbs rather than explicit therapeutic principle or treated TCM pattern. |
| Wang (2019) | Kidney-tonifying Ovulation-promoting Decoction | PK | based on core herbs: kidney-tonifying | Level 3 | Low | Excluded from sensitivity analysis | Classification was based on predominant actions of core herbs rather than explicit therapeutic principle or treated TCM pattern. |

Level 3 records excluded in the sensitivity analysis were Xu et al. (2012), Wen (2015), Li X (2018), Lu (2019), Yuan (2019), and Wang (2019).

**Part B. REML pooled estimates across classification-certainty scenarios**

| **Outcome** | **Scenario** | **Studies** | **Participants** | **Pooled MD** | **95% CI** | **95% prediction interval** | **P value** | **I²** | **Direction preserved** | **Statistical conclusion preserved** |
| --- | --- | --- | --- | --- | --- | --- | --- | --- | --- | --- |
| FSH | All studies | 37 | 2,893 | -5.563 | -8.230 to -2.896 | -22.125 to 10.999 | p = 0.001 | 99.13% | Yes | Yes |
| FSH | Excluding Level 3 | 32 | 2,519 | -5.073 | -7.842 to -2.304 | -21.262 to 11.116 | p = 0.001 | 98.96% | Yes | Yes |
| FSH | Level 1 only | 22 | 1,880 | -4.711 | -8.300 to -1.123 | -22.753 to 13.330 | p = 0.010 | 99.24% | Yes | Yes |
| LH | All studies | 35 | 2,753 | -3.005 | -4.658 to -1.353 | -12.861 to 6.851 | p = 0.001 | 98.11% | Yes | Yes |
| LH | Excluding Level 3 | 30 | 2,379 | -2.314 | -3.917 to -0.710 | -11.291 to 6.664 | p = 0.005 | 97.82% | Yes | Yes |
| LH | Level 1 only | 20 | 1,740 | -1.777 | -3.895 to 0.341 | -11.915 to 8.360 | p = 0.100 | 98.01% | Yes | No |
| E2 | All studies | 31 | 2,420 | 16.089 | 4.826 to 27.352 | -48.831 to 81.010 | p = 0.005 | 99.61% | Yes | Yes |
| E2 | Excluding Level 3 | 28 | 2,196 | 13.945 | 2.925 to 24.965 | -46.835 to 74.725 | p = 0.013 | 99.59% | Yes | Yes |
| E2 | Level 1 only | 18 | 1,557 | 14.368 | 0.653 to 28.084 | -48.648 to 77.385 | p = 0.040 | 99.58% | Yes | Yes |
| AMH | All studies | 11 | 1,125 | 0.204 | 0.031 to 0.377 | -0.459 to 0.866 | p = 0.021 | 98.65% | Yes | Yes |
| AMH | Excluding Level 3 | 11 | 1,125 | 0.204 | 0.031 to 0.377 | -0.459 to 0.866 | p = 0.021 | 98.65% | Yes | Yes |
| AMH | Level 1 only | 9 | 943 | 0.297 | -0.036 to 0.631 | -0.943 to 1.538 | p = 0.081 | 99.30% | Yes | No |

**Part C. Sensitivity interpretation summary**

| **Outcome** | **Full analysis** | **Excluding Level 3** | **Level 1 only** | **Main certainty-sensitivity conclusion** | **Strict-sensitivity conclusion** |
| --- | --- | --- | --- | --- | --- |
| FSH | MD −5.563 (−8.230 to −2.896) | MD −5.073 (−7.842 to −2.304) | MD −4.711 (−8.300 to −1.123) | Direction and significance preserved. | Direction and significance preserved. |
| LH | MD −3.005 (−4.658 to −1.353) | MD −2.314 (−3.917 to −0.710) | MD −1.777 (−3.895 to 0.341) | Direction and significance preserved. | Direction preserved, but statistical significance was not preserved. |
| E2 unit-confirmed | MD 16.089 (4.826 to 27.352) | MD 13.945 (2.925 to 24.965) | MD 14.368 (0.653 to 28.084) | Direction and significance preserved. | Direction and significance preserved, although the estimate approached the null. |
| AMH unit-confirmed | MD 0.204 (0.031 to 0.377) | Unchanged; no Level 3 studies | MD 0.297 (−0.036 to 0.631) | No Level 3 study was present; result unchanged. | Direction preserved, but statistical significance was not preserved. |

Footnote: The main classification-certainty sensitivity analysis excluded Level 3 classifications and retained Levels 1–2. The Level 1-only analysis was stricter and secondary. Classification categories were assigned at study level; all findings remain exploratory.

# Table S17. Leave-one-out sensitivity analyses for FSH, LH, E2, and AMH

| **Outcome** | **Dataset** | **Full pooled effect (95% CI)** | **Leave-one-out estimate range** | **Leave-one-out I² range** | **Most influential omitted study** | **Largest absolute change** | **Direction reversals** | **Significance changes** | **Interpretation** |
| --- | --- | --- | --- | --- | --- | --- | --- | --- | --- |
| FSH | Main | -5.563 (-8.230 to -2.896) | -5.903 to -4.795 | 98.74% to 99.18% | Li (2012) | 0.768 | 0 | 0 | Direction and statistical significance were preserved in every leave-one-out analysis. |
| LH | Main | -3.005 (-4.658 to -1.353) | -3.337 to -2.599 | 97.64% to 98.22% | Wen (2015) | 0.406 | 0 | 0 | Direction and statistical significance were preserved in every leave-one-out analysis. |
| E2 | Unit-confirmed sensitivity | 16.089 (4.826 to 27.352) | 13.493 to 18.598 | 99.32% to 99.64% | Lu (2019) | 2.596 | 0 | 0 | Direction and statistical significance were preserved in every leave-one-out analysis. |
| AMH | Unit-confirmed sensitivity | 0.204 (0.031 to 0.377) | 0.106 to 0.261 | 87.30% to 99.43% | Ye (2024) | 0.097 | 0 | 6 | Direction was preserved in every analysis, but statistical significance changed in six leave-one-out analyses. |

Note: Each REML model was refitted after omitting one study. E2 and AMH used the unit-confirmed sensitivity datasets. The analyses assess dependence on individual studies and do not resolve the substantial between-study heterogeneity.

# Table S18. Endpoint-value and change-from-baseline data availability audit

| **Outcome** | **Final endpoint studies** | **Baseline mean/SD available** | **Endpoint mean/SD available** | **Descriptive change calculable** | **Paired change SD or adjusted effect reported** | **Formal change-score meta-analysis feasible** |
| --- | --- | --- | --- | --- | --- | --- |
| FSH | 37 | 34 | 37 | 33 | No | No |
| LH | 35 | 33 | 35 | 31 | No | No |
| E2 | 35 | 32 | 35 | 29 | No | No |
| AMH | 12 | 11 | 12 | 10 | No | No |
| AFC | 8 | 7 | 8 | 7 | No | No |
| PSV | 5 | 5 | 5 | 5 | No | No |

Baseline and endpoint means and SDs were directly reported in the original trial reports. A descriptive endpoint-minus-baseline change was considered calculable only when complete arm-specific baseline and endpoint data were available and the reported units were compatible or could be standardized using a verified conversion. Paired change SDs, baseline–endpoint correlations, and adjusted between-group effects were not available in the supplied source-level extraction; therefore, a formal change-score meta-analysis was not feasible. No change-score SD was derived, and no assumption of independence between baseline and endpoint measurements was applied. The endpoint-value meta-analysis was therefore retained.

# Table S19. Small-study-effect assessments and trim-and-fill sensitivity analyses for outcomes with at least 10 studies

Egger regression tests and trim-and-fill sensitivity analyses were performed for outcomes with at least 10 studies, including the final adverse-event odds-ratio dataset. A two-sided p value <0.10 was treated as a possible small-study-effect signal. Funnel asymmetry and trim-and-fill results were interpreted as exploratory and were not considered definitive evidence for or against publication bias.

| **Outcome** | **Studies** | **Funnel plot** | **Egger p value** | **Small-study-effect signal** | **Trim-and-fill imputed studies** | **Side** | **Observed pooled effect (95% CI)** | **Adjusted effect (95% CI)** | **Interpretation** | **Notes** |
| --- | --- | --- | --- | --- | --- | --- | --- | --- | --- | --- |
| FSH | 37 | Yes | 0.938 | No statistical signal | 0 | Right | MD -5.563 (-8.230 to -2.896) | MD -5.563 (-8.230 to -2.896) | Egger test did not indicate a signal and trim-and-fill did not alter the pooled estimate. | Exploratory; heterogeneity can generate funnel asymmetry. |
| LH | 35 | Yes | 0.231 | No statistical signal | 0 | Right | MD -3.005 (-4.658 to -1.353) | MD -3.005 (-4.658 to -1.353) | Egger test did not indicate a signal and trim-and-fill did not alter the pooled estimate. | Exploratory; heterogeneity can generate funnel asymmetry. |
| E2 | 31 | Yes | 0.355 | No statistical signal | 0 | Left | MD 16.089 (4.826 to 27.352) | MD 16.089 (4.826 to 27.352) | Egger test did not indicate a signal and trim-and-fill did not alter the pooled estimate. | Exploratory; heterogeneity can generate funnel asymmetry. |
| AMH | 11 | Yes | <0.001 | Possible signal | 0 | Left | MD 0.204 (0.031 to 0.377) | MD 0.204 (0.031 to 0.377) | Egger test indicated a signal, but only 11 studies were available, heterogeneity was extreme, and trim-and-fill imputed no studies. | Exploratory; heterogeneity can generate funnel asymmetry. |
| Overall clinical effectiveness | 34 | Yes | 0.003 | Possible signal | 9 | Left | OR 2.187 (1.762 to 2.715) | OR 1.844 (1.498 to 2.270) | Nine studies were imputed on the left; the adjusted OR was attenuated but remained above the null. | Supplementary composite outcome only; not menstrual improvement. |
| Adverse events OR | 14 | Yes | 0.739 | No statistical signal | 4 | Right | OR 0.198 (0.109 to 0.359) | OR 0.291 (0.151 to 0.560) | Egger testing did not indicate a statistically detectable small-study-effect signal. Trim-and-fill imputed four studies on the right and attenuated the pooled association toward the null, although the adjusted estimate remained below 1. | Final 14-study OR dataset; 1,114 participants; double-zero studies excluded; 0.5 continuity correction applied only to studies with a zero cell. |

**Notes**

MD, mean difference; OR, odds ratio; CI, confidence interval. E2 and AMH analyses used the unit-confirmed datasets.

AFC and PSV were not assessed because fewer than 10 studies were available. Menstrual return/resumption had four studies.

Trim-and-fill was exploratory; imputed studies are hypothetical and the adjusted estimates do not replace the main REML results.

Overall clinical effectiveness was retained only as a supplementary variably defined composite outcome.

# Table S20. Full GRADE evidence profile

**Part A. Outcome, role, and pooled-effect estimates**

| **Outcome** | **Outcome role** | **Studies** | **Participants** | **Effect measure** | **Pooled effect** | **95% CI** | **I²** |
| --- | --- | --- | --- | --- | --- | --- | --- |
| Menstrual return or resumption | Patient-important menstrual outcome | 4 | 266 | Odds ratio | 1.523 | 0.315 to 7.371 | 86.16% |
| Overall clinical effectiveness | Supplementary variably defined composite outcome | 34 | 2,807 | Odds ratio | 2.187 | 1.762 to 2.715 | 9.26% |
| FSH | Primary surrogate biomarker | 37 | 2,893 | Mean difference | −5.563 | −8.230 to −2.896 | 99.13% |
| AMH | Primary surrogate biomarker | 12 | 1,198 | Mean difference | 0.167 | 0.035 to 0.299 | 98.17% |
| LH | Secondary endocrine surrogate | 35 | 2,753 | Mean difference | −3.005 | −4.658 to −1.353 | 98.11% |
| E2 | Secondary endocrine surrogate | 35 | 2,693 | Mean difference | 22.483 | 6.924 to 38.042 | 99.80% |
| AFC | Secondary ovarian reserve-related surrogate | 8 | 688 | Mean difference | 0.457 | 0.028 to 0.886 | 94.95% |
| PSV | Exploratory hemodynamic outcome | 5 | 477 | Mean difference | 0.570 | 0.444 to 0.697 | 0.00% |
| Adverse events | Safety outcome | 14 OR; 22 RD | 1,114 OR; 1,755 RD | Odds ratio (main); risk difference (sensitivity) | OR 0.198; RD −0.077 | OR 0.109 to 0.359; RD −0.128 to −0.025 | OR 16.67%; RD 77.45% |

**Part B. GRADE domain judgments and downgrading decisions**

| **Outcome** | **Risk of bias judgment** | **Risk of bias downgrade** | **Inconsistency judgment** | **Inconsistency downgrade** | **Indirectness judgment** | **Indirectness downgrade** | **Imprecision judgment** | **Imprecision downgrade** | **Publication bias judgment** | **Publication bias downgrade** |
| --- | --- | --- | --- | --- | --- | --- | --- | --- | --- | --- |
| Menstrual return or resumption | Serious concern: allocation concealment, blinding, outcome-assessor blinding, incomplete-outcome-data handling, and selective reporting were mostly unclear. Menstrual outcomes were also vulnerable to detection and reporting bias. | -1 | Very serious concern: I² = 86.16%, effects varied materially, and the 95% prediction interval was 0.001 to 2166.521; heterogeneity remained unexplained. | -2 | Serious concern: the outcome was patient-important, but definitions ranged from normal or natural menstruation to menstrual resumption and periodic vaginal bleeding; one study used a broader definition. | -1 | Very serious concern: only 4 studies (266 participants) were available; the 95% CI (0.315 to 7.371) crossed the null and included substantial benefit, no effect, and possible harm. | -2 | Not formally assessable because k = 4. No automatic downgrade was applied solely because funnel-plot testing was infeasible; selective outcome reporting cannot be excluded. | 0 |
| Overall clinical effectiveness | Serious concern: allocation concealment and blinding were frequently unclear, and this subjective composite outcome was vulnerable to performance, detection, and selective-reporting bias. | -1 | No serious concern: statistical heterogeneity was low (I² = 9.26%) and the 95% prediction interval (OR 1.388 to 3.447) did not cross the null. | 0 | Very serious concern: definitions varied substantially and could combine symptoms, menstrual changes, laboratory indicators, and investigator-defined response. It is not a standardized patient-important endpoint. | -2 | No serious concern: 34 studies and 2,807 participants were available, and the 95% CI did not cross the null. No established MCID was available. | 0 | Serious concern: Egger testing suggested small-study effects (p = 0.0027). Trim-and-fill imputed 9 studies on the left and attenuated the pooled OR from 2.187 to 1.844 (95% CI 1.498 to 2.270), although the adjusted CI remained above 1. | -1 |
| FSH | Serious concern: random sequence generation was generally adequate, but allocation concealment, blinding, outcome-assessor blinding, incomplete-outcome-data handling, and selective reporting were mostly unclear. | -1 | Very serious concern: I² = 99.13%, residual heterogeneity remained extreme after subgroup and meta-regression analyses, and the 95% prediction interval (−22.125 to 10.999) crossed the null. | -2 | Serious concern: FSH is a surrogate endocrine biomarker and does not directly establish menstrual recovery, fertility, live birth, or sustained ovarian function. | -1 | No additional downgrade: the pooled 95% CI excluded the null and the information size was large; prediction-interval uncertainty was already counted under inconsistency. | 0 | No serious concern based on formal exploratory analyses: Egger p = 0.938 and trim-and-fill imputed 0 studies. Extreme heterogeneity still limits funnel-plot interpretation. | 0 |
| AMH | Serious concern: random sequence generation was generally adequate, but allocation concealment, blinding, outcome-assessor blinding, incomplete-outcome-data handling, and selective reporting were mostly unclear. | -1 | Very serious concern: I² = 98.17%, the 95% prediction interval (−0.347 to 0.681) crossed the null, and heterogeneity remained extreme in the unit-confirmed analysis. | -2 | Serious concern: AMH is a surrogate ovarian-reserve biomarker and does not directly establish recovery of ovarian function, menstruation, pregnancy, or live birth. | -1 | Serious concern: the pooled CI was close to the null, only 12 studies were available, statistical significance changed in 6 leave-one-out analyses, and the Level-1-only analysis crossed the null. | -1 | Serious concern: Egger testing indicated a possible small-study-effect signal (p = 0.001). Trim-and-fill imputed 0 studies, but only 11 unit-confirmed studies were available and heterogeneity was extreme. | -1 |
| LH | Serious concern: random sequence generation was generally adequate, but allocation concealment, blinding, outcome-assessor blinding, incomplete-outcome-data handling, and selective reporting were mostly unclear. | -1 | Very serious concern: I² = 98.11%, prespecified moderators did not explain heterogeneity, and the 95% prediction interval (−12.861 to 6.851) crossed the null. | -2 | Serious concern: LH is a surrogate endocrine biomarker rather than a direct patient-important outcome. | -1 | No additional downgrade: the pooled 95% CI excluded the null and the information size was large; prediction-interval uncertainty was already counted under inconsistency. | 0 | No serious concern based on formal exploratory analyses: Egger p = 0.231 and trim-and-fill imputed 0 studies. Extreme heterogeneity still limits funnel-plot interpretation. | 0 |
| E2 | Serious concern: random sequence generation was generally adequate, but allocation concealment, blinding, outcome-assessor blinding, incomplete-outcome-data handling, and selective reporting were mostly unclear. | -1 | Very serious concern: I² = 99.80%, prespecified moderators did not explain heterogeneity, and the 95% prediction interval (−73.121 to 118.087) crossed the null. | -2 | Serious concern: E2 is a surrogate endocrine biomarker rather than a direct patient-important outcome. | -1 | No additional downgrade: the pooled 95% CI excluded the null and the information size was large; prediction-interval uncertainty was already counted under inconsistency. Unit-confirmed sensitivity preserved direction. | 0 | No serious concern in the unit-confirmed dataset: Egger p = 0.355 and trim-and-fill imputed 0 studies. Extreme heterogeneity still limits funnel-plot interpretation. | 0 |
| AFC | Serious concern: random sequence generation was generally adequate, but allocation concealment, blinding, outcome-assessor blinding, incomplete-outcome-data handling, and selective reporting were mostly unclear. | -1 | Very serious concern: I² = 94.95% and the 95% prediction interval (−1.079 to 1.992) crossed the null; heterogeneity remained unexplained. | -2 | Serious concern: AFC is a surrogate ovarian-reserve measure and does not directly establish patient-important recovery or fertility benefit. | -1 | Serious concern: only 8 studies (688 participants) were available, the pooled CI was close to the null, and no established MCID was available. | -1 | Not formally assessable because k = 8. No automatic downgrade was applied solely because funnel-plot testing was infeasible. | 0 |
| PSV | Serious concern: random sequence generation was generally adequate, but allocation concealment, blinding, outcome-assessor blinding, incomplete-outcome-data handling, and selective reporting were mostly unclear. | -1 | No serious concern: I² = 0.00% and the 95% prediction interval (0.365 to 0.776) did not cross the null. | 0 | Very serious concern: PSV is an exploratory ovarian hemodynamic measure with an uncertain and indirect link to menstrual recovery, fertility, or long-term patient benefit. | -2 | No serious concern based on statistical precision: the 95% CI and prediction interval did not cross the null. The absence of an established MCID was addressed under indirectness rather than counted twice. | 0 | Not formally assessable because k = 5. No automatic downgrade was applied solely because funnel-plot testing was infeasible. | 0 |
| Adverse events | Serious concern: adverse-event ascertainment, severity, causality, follow-up, and handling of unreported events were incompletely described; absence of reporting was not treated as zero. | -1 | Serious concern: heterogeneity was low for the 14-study OR analysis (I² = 16.67%) but high for the 22-study RD sensitivity analysis (I² = 77.45%), whose prediction interval crossed the null. | -1 | Serious concern: adverse-event definitions, monitoring intensity, severity grading, relatedness assessment, and analysis populations were not standardized across trials. | -1 | No additional downgrade: the main OR analysis included 1,114 participants and 106 events, and its 95% CI and prediction interval did not cross the null. RD uncertainty was counted under inconsistency. | 0 | No serious concern based on formal exploratory testing: the REML Egger test did not indicate a statistically detectable small-study-effect signal (p = 0.739). Trim-and-fill imputed four studies on the right and attenuated the pooled OR from 0.198 to 0.291 (95% CI 0.151 to 0.560), although the adjusted confidence interval remained below 1. Because adverse-event ascertainment and reporting were incomplete and non-standardized, publication bias could not be excluded. | 0 |

**Part C. Overall certainty, reasons, and manuscript comments**

| **Outcome** | **Overall certainty** | **Main reasons for downgrading** | **Interpretive note** |
| --- | --- | --- | --- |
| Menstrual return or resumption | Very low | Risk of bias; very serious inconsistency; indirectness from heterogeneous menstrual definitions; very serious imprecision. | This is the most patient-relevant efficacy outcome, but only 4 studies were available and the CI and prediction interval were extremely wide. The evidence does not support a conclusion of menstrual-restoration efficacy. |
| Overall clinical effectiveness | Very low | Risk of bias; very serious indirectness from a variably defined composite outcome; suspected small-study effects/publication bias. | Supplementary outcome only. Trim-and-fill attenuated the OR from 2.187 to 1.844; This variably defined composite outcome should not be interpreted as menstrual improvement or as primary evidence of clinical efficacy. |
| FSH | Very low | Risk of bias; very serious inconsistency; indirectness because FSH is a surrogate biomarker. | The average MD favored oral Chinese herbal formulas, but the prediction interval crossed the null and heterogeneity remained unexplained. |
| AMH | Very low | Risk of bias; very serious inconsistency; indirectness; imprecision; possible small-study effects. | Interpret alongside the unit-confirmed and leave-one-out analyses. Direction was generally preserved, but statistical significance was unstable. |
| LH | Very low | Risk of bias; very serious inconsistency; indirectness because LH is a surrogate biomarker. | The average MD favored oral Chinese herbal formulas, but the prediction interval crossed the null and prespecified moderators did not explain heterogeneity. |
| E2 | Very low | Risk of bias; very serious inconsistency; indirectness because E2 is a surrogate biomarker. | Unit-confirmed sensitivity preserved the direction, but the prediction interval crossed the null and heterogeneity remained extreme. |
| AFC | Very low | Risk of bias; very serious inconsistency; indirectness; imprecision. | AFC is a surrogate ovarian-reserve outcome. Only 8 studies were available and the prediction interval crossed the null. |
| PSV | Very low | Risk of bias; very serious indirectness. | The estimate was statistically precise, but PSV is an exploratory hemodynamic endpoint and should not support menstrual, fertility, restorative, or patient-benefit claims. |
| Adverse events | Very low | Risk of bias; inconsistency between OR and RD analyses; indirectness from incomplete and non-standardized safety ascertainment. | The numerical direction favored oral Chinese herbal formulas in both the OR and RD analyses. Formal small-study-effect testing did not identify a statistically detectable Egger signal, while trim-and-fill attenuated but did not reverse the OR estimate. Safety certainty nevertheless remains very low because adverse-event ascertainment, definitions, severity grading, and reporting were incomplete and non-standardized. |

GRADE certainty was re-rated using the final REML results. Randomized evidence started at high certainty and was assessed for risk of bias, inconsistency, indirectness, imprecision, and publication bias; no upgrading factors were applied. Because no established or prespecified MCIDs were available, imprecision was judged conservatively from the null threshold, confidence-interval width, information size or event count, and stability across sensitivity analyses. Prediction intervals primarily informed inconsistency, and the same uncertainty was not downgraded twice. Publication-bias judgments were informed by Table S19 where formal assessment was available; inability to test outcomes with fewer than 10 studies did not alone trigger downgrading. Overall clinical effectiveness was graded for completeness but remained a supplementary, variably defined composite outcome.

All nine outcomes were rated as very-low-certainty evidence. This reflects uncertainty in the evidence, not proof that the interventions have no effect.

**Supplementary Methods. Exploratory network pharmacology analysis**
This supplementary analysis was conducted solely as hypothesis-generating context and was not used to support clinical efficacy, therapeutic-principle superiority, or causal mechanisms. Herb frequency was calculated separately within the PK, KB, and KL therapeutic-principle subgroups, with each herb counted once per study regardless of dose or repeated mention. Six high-frequency core herbs were selected within each subgroup. Studies without an extractable fixed prescription were excluded from the subgroup-specific frequency denominator, and ties at the sixth-position boundary were resolved using the rule reported in Table S21.

Candidate compounds and compound-associated targets were obtained from the Traditional Chinese Medicine Systems Pharmacology database using the prespecified screening thresholds of oral bioavailability ≥30% and drug-likeness ≥0.18. Target names were standardized to human gene symbols using UniProt mapping. POI-associated targets were assembled from GeneCards, DisGeNET, and OMIM. The raw standardized union contained 8,865 genes. The strict POI core set comprised 105 genes supported by at least two of the three disease databases or by explicit POI/POF evidence in OMIM. A broader sensitivity set of 486 genes additionally included single-database GeneCards targets with relevance scores at or above the dataset-specific 95th percentile (Q95 = 31.896).

Drug–disease target intersections were calculated separately for PK, KB, and KL. Dependence on the POI disease-target definition was examined by comparing strict-core and sensitivity-set intersections and calculating the corresponding Jaccard similarities. All results were interpreted descriptively. Predicted intersections were not regarded as validated therapeutic targets, and between-category differences were not interpreted as evidence of differential clinical efficacy.

**Table S21. Six high-frequency core herbs selected within each therapeutic-principle subgroup**

| **Subgroup** | **Analyzable studies, n** | **Selection order** | **Herb** | **Frequency within subgroup** | **Prescription coverage within subgroup** |
| --- | --- | --- | --- | --- | --- |
| PK | 15 | 1 | Rehmanniae Radix Praeparata (Shu Di Huang) | 14 | 93.3% |
| PK | 15 | 2 | Cuscutae Semen (Tu Si Zi) | 11 | 73.3% |
| PK | 15 | 3 | Corni Fructus (Shan Zhu Yu) | 10 | 66.7% |
| PK | 15 | 4 | Angelicae Sinensis Radix (Dang Gui) | 9 | 60.0% |
| PK | 15 | 5 | Dioscoreae Rhizoma (Shan Yao) | 9 | 60.0% |
| PK | 15 | 6 | Poria (Fu Ling) | 7 | 46.7% |
| KB | 14 | 1 | Angelicae Sinensis Radix (Dang Gui) | 13 | 92.9% |
| KB | 14 | 2 | Rehmanniae Radix Praeparata (Shu Di Huang) | 11 | 78.6% |
| KB | 14 | 3 | Cuscutae Semen (Tu Si Zi) | 10 | 71.4% |
| KB | 14 | 4 | Salviae Miltiorrhizae Radix et Rhizoma (Dan Shen) | 9 | 64.3% |
| KB | 14 | 5 | Epimedii Folium (Yin Yang Huo) | 8 | 57.1% |
| KB | 14 | 6 | Morindae Officinalis Radix (Ba Ji Tian) | 7 | 50.0% |
| KL | 11 | 1 | Rehmanniae Radix Praeparata (Shu Di Huang) | 9 | 81.8% |
| KL | 11 | 2 | Angelicae Sinensis Radix (Dang Gui) | 8 | 72.7% |
| KL | 11 | 3 | Cuscutae Semen (Tu Si Zi) | 8 | 72.7% |
| KL | 11 | 4 | Paeoniae Radix Alba (Bai Shao) | 7 | 63.6% |
| KL | 11 | 5 | Cyperi Rhizoma (Xiang Fu) | 7 | 63.6% |
| KL | 11 | 6 | Bupleuri Radix (Chai Hu) | 6 | 54.5% |

Note. Frequency was defined as the number of studies within each therapeutic-principle subgroup in which an herb appeared at least once in the fixed oral formula. Each herb was counted once per study, regardless of dose or repeated mention. Prescription coverage was calculated using the number of studies with an extractable fixed herbal composition within each subgroup as the denominator (PK, n=15; KB, n=14; KL, n=11). Individualized adjunct herbs were excluded. Jiang (2016) was excluded from the PK frequency calculation because no single fixed prescription could be extracted. Ties at the sixth-position boundary were resolved by ascending alphabetical order of standardized pinyin names without tone marks. Boundary ties were Poria and Lycii Fructus in PK; Morindae Officinalis Radix, Codonopsis Radix, and Hominis Placenta in KB; and Bupleuri Radix and Dioscoreae Rhizoma in KL.

Abbreviations: PK, pure kidney-tonifying; KB, kidney-tonifying and blood-activating; KL, kidney-tonifying and liver-soothing.

**Table S22. Summary of target sets and strict POI intersections**

**Part A. Target-set summary**

| **Target set** | **Group or tier** | **Target count** | **Definition** | **Role in analysis** |
| --- | --- | --- | --- | --- |
| Drug-target sets | PK drug-target set | 228 | Verified human gene targets derived from the six selected core herbs in the subgroup. | Main exploratory intersection |
| Drug-target sets | KB drug-target set | 258 | Verified human gene targets derived from the six selected core herbs in the subgroup. | Main exploratory intersection |
| Drug-target sets | KL drug-target set | 247 | Verified human gene targets derived from the six selected core herbs in the subgroup. | Main exploratory intersection |
| POI target sets | Raw standardized POI union | 8865 | The standardized union of POI/POF-associated genes retrieved from GeneCards, DisGeNET, and OMIM before evidence filtering. | Descriptive audit only |
| POI target sets | Strict POI core set | 105 | Genes supported by at least two of the three independent disease databases (GeneCards, DisGeNET, and OMIM), or by explicit POI/POF evidence in OMIM. | Main exploratory intersection |
| POI target sets | POI sensitivity set | 486 | The strict core set plus single-database GeneCards targets with relevance scores at or above the dataset-specific 95th percentile (Q95 = 31.896). | Sensitivity assessment |

**Part B. Strict POI intersections**

| **Subgroup** | **Drug-target count** | **Strict POI core count** | **Intersection count** | **Intersecting targets** | **Subgroup-specific strict targets** |
| --- | --- | --- | --- | --- | --- |
| PK | 228 | 105 | 3 | CHEK2; ESR2; PTEN | — |
| KB | 258 | 105 | 3 | CHEK2; ESR2; PTEN | — |
| KL | 247 | 105 | 3 | CHEK2; ESR2; PTEN | — |

Note. The same three predicted intersections (CHEK2, ESR2, and PTEN) were identified in all three subgroups, and no subgroup-specific strict intersection was observed.

Abbreviations: PK, pure kidney-tonifying; KB, kidney-tonifying and blood-activating; KL, kidney-tonifying and liver-soothing; POI, premature ovarian insufficiency; POF, premature ovarian failure; OMIM, Online Mendelian Inheritance in Man.

# Table S23. Sensitivity analysis of target intersections according to the POI disease-target definition

| **Subgroup** | **Strict intersection count** | **Sensitivity intersection count** | **Shared strict targets** | **Core-sensitivity Jaccard similarity** | **Stability assessment** |
| --- | --- | --- | --- | --- | --- |
| PK | 3 | 52 | CHEK2; ESR2; PTEN | 0.058 | Highly sensitive to disease-target definition |
| KB | 3 | 57 | CHEK2; ESR2; PTEN | 0.053 | Highly sensitive to disease-target definition |
| KL | 3 | 55 | CHEK2; ESR2; PTEN | 0.055 | Highly sensitive to disease-target definition |

Note. The broader sensitivity target set produced substantially more intersections in all three subgroups, indicating strong dependence on the POI disease-target definition.

Abbreviations: PK, pure kidney-tonifying; KB, kidney-tonifying and blood-activating; KL, kidney-tonifying and liver-soothing; POI, premature ovarian insufficiency.

**Figure S1. Additional forest plots for outcomes not displayed in the main Figure 3.**

All pooled estimates were calculated using REML random-effects models. Forest plots display study-specific effects and 95% confidence intervals; 95% prediction intervals are reported in Table S10 and were not drawn. The null value was 0 for mean differences and risk differences and 1 for odds ratios. Overall clinical effectiveness is a supplementary composite outcome and is not menstrual improvement.

Figure S1A. Luteinizing hormone (LH) overall forest plot.

Figure S1B. Estradiol (E2) overall forest plot.

Figure S1C. Anti-Müllerian hormone (AMH) overall forest plot.

Figure S1D. Antral follicle count (AFC) overall forest plot.

Figure S1E. Ovarian arterial peak systolic velocity (PSV) overall forest plot.

Figure S1F. Overall clinical effectiveness forest plot (supplementary composite outcome).

Figure S1G. Adverse-event odds-ratio forest plot.

Figure S1H. Adverse-event risk-difference forest plot including double-zero studies.

**Figure S2. Exploratory therapeutic-principle subgroup forest plots.**

Therapeutic-principle categories were kidney-tonifying alone (PK), kidney-tonifying plus blood-activating (KB), and kidney-tonifying plus liver-soothing (KL). The PK/KL/KB subgroup framework was prespecified, whereas the Level 1–3 classification-certainty scheme was added after registration to improve transparency and support sensitivity analysis. All subgroup analyses were exploratory and hypothesis-generating; residual heterogeneity remained substantial in several subgroups. E2 was analyzed using the unit-confirmed dataset.

Figure S2A. FSH therapeutic-principle subgroup forest plot.

Figure S2B. LH therapeutic-principle subgroup forest plot.

Figure S2C. Unit-confirmed E2 therapeutic-principle subgroup forest plot.

**Figure S3. Unit-confirmed and classification-certainty sensitivity forest plots.**

Unit-confirmed analyses excluded studies with unverifiable E2 or AMH unit assumptions. Classification-certainty analyses excluded Level 3 therapeutic-principle classifications and retained Levels 1 and 2. These analyses assessed robustness and were not used to establish differential efficacy.

Figure S3A. E2 unit-confirmed sensitivity-analysis forest plot.

Figure S3B. AMH unit-confirmed sensitivity-analysis forest plot.

Figure S3C. FSH classification-certainty sensitivity forest plot excluding Level 3 studies.

Figure S3D. LH classification-certainty sensitivity forest plot excluding Level 3 studies.

Figure S3E. Unit-confirmed E2 classification-certainty sensitivity forest plot excluding Level 3 studies.

**Figure S4. Leave-one-out sensitivity analyses.**

Each REML model was refitted after omitting one study at a time. The vertical effect-size reference line represents the pooled estimate from the full dataset. FSH, LH, and unit-confirmed E2 retained their direction and statistical significance in every analysis; AMH retained its direction, but statistical significance changed in six analyses.

Figure S4A. FSH leave-one-out sensitivity analysis.

Figure S4B. LH leave-one-out sensitivity analysis.

Figure S4C. Unit-confirmed E2 leave-one-out sensitivity analysis.

Figure S4D. Unit-confirmed AMH leave-one-out sensitivity analysis.

**Figure S5. Contour-enhanced funnel plots and trim-and-fill sensitivity analyses.**

Contour-enhanced funnel plots and trim-and-fill analyses are shown for FSH, LH, unit-confirmed E2, unit-confirmed AMH, overall clinical effectiveness, and the adverse-event odds-ratio dataset. The adverse-event plots use the 14 studies contributing to the OR analysis; double-zero studies are excluded. Funnel asymmetry may reflect heterogeneity, sparse events, clinical diversity, selective reporting, or other small-study effects and does not establish publication bias. Trim-and-fill imputations are hypothetical. Numerical results for all assessed outcomes are reported in Table S19.

Figure S5A. FSH contour-enhanced funnel plot.

Figure S5B. FSH trim-and-fill funnel plot.

Figure S5C. LH contour-enhanced funnel plot.

Figure S5D. LH trim-and-fill funnel plot.

Figure S5E. Unit-confirmed E2 contour-enhanced funnel plot.

Figure S5F. Unit-confirmed E2 trim-and-fill funnel plot.

Figure S5G. Unit-confirmed AMH contour-enhanced funnel plot.

Figure S5H. Unit-confirmed AMH trim-and-fill funnel plot.

Figure S5I. Overall clinical effectiveness contour-enhanced funnel plot.

Figure S5J. Overall clinical effectiveness trim-and-fill funnel plot.

Figure S5K. Adverse events: contour-enhanced funnel plot based on the odds-ratio dataset.

Figure S5L. Adverse events: trim-and-fill sensitivity analysis based on the odds-ratio dataset.
